# Molecular Recognition by Halogen Bonding With Atomic Precision in a Covalent Organic Cage

**DOI:** 10.1002/anie.5640364

**Published:** 2026-09-27

**Authors:** Pierre‐Antoine Dupin, Jian Yang, Elise Antonetti, Bastien Chatelet, Xavier Bugaut, Jean‐Pierre Dutasta, Paola Nava, Anne‐Doriane Manick, David Bardelang, Alexandre Martinez

**Affiliations:** ^1^ Aix Marseille Univ, CNRS, Centrale Med, iSm2 Marseille France; ^2^ State Key Laboratory of Structural Chemistry Fujian Institute of Research on the Structure of Matter, Chinese Academy of Sciences Fuzhou China; ^3^ IPCM, UMR 8232 Sorbonne Univ, CNRS Paris France; ^4^ LIMA, UMR 7042 Univ Strasbourg, Univ Haute Alsace, CNRS Strasbourg France; ^5^ LCH UMR 5182 CNRS, ENS de Lyon, Lyon 1 Université Lyon France; ^6^ Aix Marseille Univ, CNRS, ICR Marseille France

**Keywords:** covalent organic cages, haloazaphosphatranes, halogen bonding, molecular recognition, organophosphorus

## Abstract

Halogen bonding (XB) is a recognition motif of increasing importance but it remains underused in solution. Embedding a single XB motif within a molecular capsule improves both its stability and interaction strength by finely tuning inward position and orientation. In this context, a new Covalent Organic Cage (COC) was prepared sequestering a polarized P‐Cl bond. This hemicryptophane carrying a haloazaphosphatrane moiety was found to bind dimethyl methylphosphonate (DMMP), a Sarin surrogate, by halogen bonding with atomic precision. Indeed, the same cage having the chlorine atom substituted by a hydrogen atom showed no detectable recognition. Such level of discrimination definitely establishes halogen bonding COC as a new generation of efficient receptors for molecular recognition.

## Graphical abstract

A covalent organic cage embedding a single inward‐facing halogen‐bond (XB) donor enables recognition of DMMP, a Sarin surrogate, with atomic precision through halogen bonding, while both the hydrogen bonding donor encaged analogue and the XB donor open model ‐lacking cavity‐ show no affinity. Confinement within the cage enhances XB strength and selectivity, highlighting a new strategy for efficient molecular recognition.

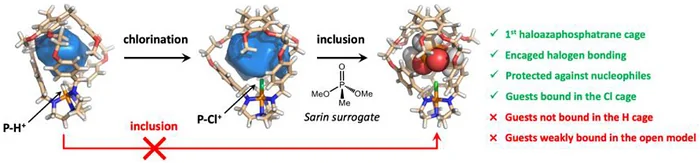

Self‐assembled or covalent molecular containers can accommodate guest molecules within their cavity [1, 2, 3, 4, 5, 6, 7, 8], leading to numerous and various applications ranging from drug delivery to sensing or extraction [9, 10, 11, 12, 13, 14, 15, 16, 17, 18, 19, 20, 21]. Once encapsulated in the confined space of a container and isolated from the bulk solution by sufficiently large energy barriers, guest molecules may exhibit new behaviors including stabilization of highly reactive species, reaction intermediates or transition states [22, 23, 24, 25, 26, 27, 28, 29, 30, 31, 32, 33, 34, 35, 36, 37, 38, 39]. A variety of interactions contribute to the formation and stability of host‐guest complexes such as hydrogen bonding, dispersion interactions, cation‐π or anion‐π interactions [40, 41, 42]. In this context, halogen bonding (XB) has been used to stabilize host‐guest complexes [43, 44, 45, 46, 47, 48, 49, 50, 51, 52, 53, 54], or to form container assemblies [55, 56, 57]. Receptors like macrocycles and rotaxanes bearing halogen bond donors have been described [58, 59, 60, 61, 62, 63, 64, 65, 66]. Moreover, J. Rebek previously reported that isolating XB donors and acceptors as guests co‐encapsulated within a single capsule allows direct observation of halogen bonding [67, 68]. In this case, the cavity imposes a strict alignment of the two partners, leading to a favorable XB arrangement. However, to our knowledge XB donor motifs have never been embedded within a Covalent Organic Cage (COC) for anion or neutral molecule recognition despite expected improved properties caused by confinement effects. Thus, we decided to determine if immobilization of an XB motif within a molecular cavity impacts its recognition properties. We have previously shown that chloroazaphosphatranes, the chlorinated derivatives of Verkade's superbases [69, 70, 71, 72], represent a new class of XB donor motifs capable of recognizing anions and acting as organocatalysts through halogen bonding [73]. Therefore, we herein describe our results related to the covalent integration of a chloroazaphosphatrane XB donor inside a hemicryptophane cage and how this confinement impacts XB molecular recognition (Figure 1).

**FIGURE 1:**
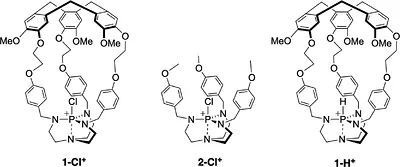
Structures of the receptors used in this study. Counter‐ion (BarF^−^) is omitted for clarity.

The encaged Verkade's superbase **1** was obtained according to our previous reported procedure [74]. Pro‐azaphosphatrane **1** was allowed to react with hexachloroethane for two hours at room temperature to afford the confined chloroazaphosphatrane **1‐Cl^+^·Cl^–^** in 60% yield (Scheme 1). Subsequent anion metathesis provided the expected cage **1‐Cl^+^·BarF^–^** (BarF^−^: tetrakis[3,5‐bis(trifluoromethyl)phényl]borate).

**SCHEME 1:**
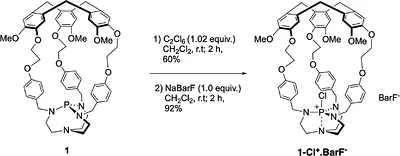
Synthesis of 1‐Cl^+^.BarF^–^.

The ^1^H and ^13^C NMR spectra of **1‐Cl^+^^.^Cl^–^** are indicative of a time‐average *C_3_* symmetry in CD_2_Cl_2_ solution (Figure S20). Single‐crystal X‐ray diffraction analysis confirms the proposed structure and the average *C_3_* symmetry is retained in the solid. The molecular cage displays endohedral functionalization of its cavity by an XB motif, with the P‐Cl bond clearly oriented inwards (Figure 2a). Furthermore, a chloroform molecule is trapped inside the well‐defined cavity and is involved in the XB formation between the chloroazaphosphatrane unit and the chloroform guest (Figure S16). The near‐linearity of the P‐Cl···Cl angle (158°) and the P‐Cl···Cl distance (around 3.48 Å), shorter than the sum of their respective van der Waals radii (3.6 Å), are consistent with the formation of the halogen bond, thus supporting the formation of XB in the solid state.

**FIGURE 2:**
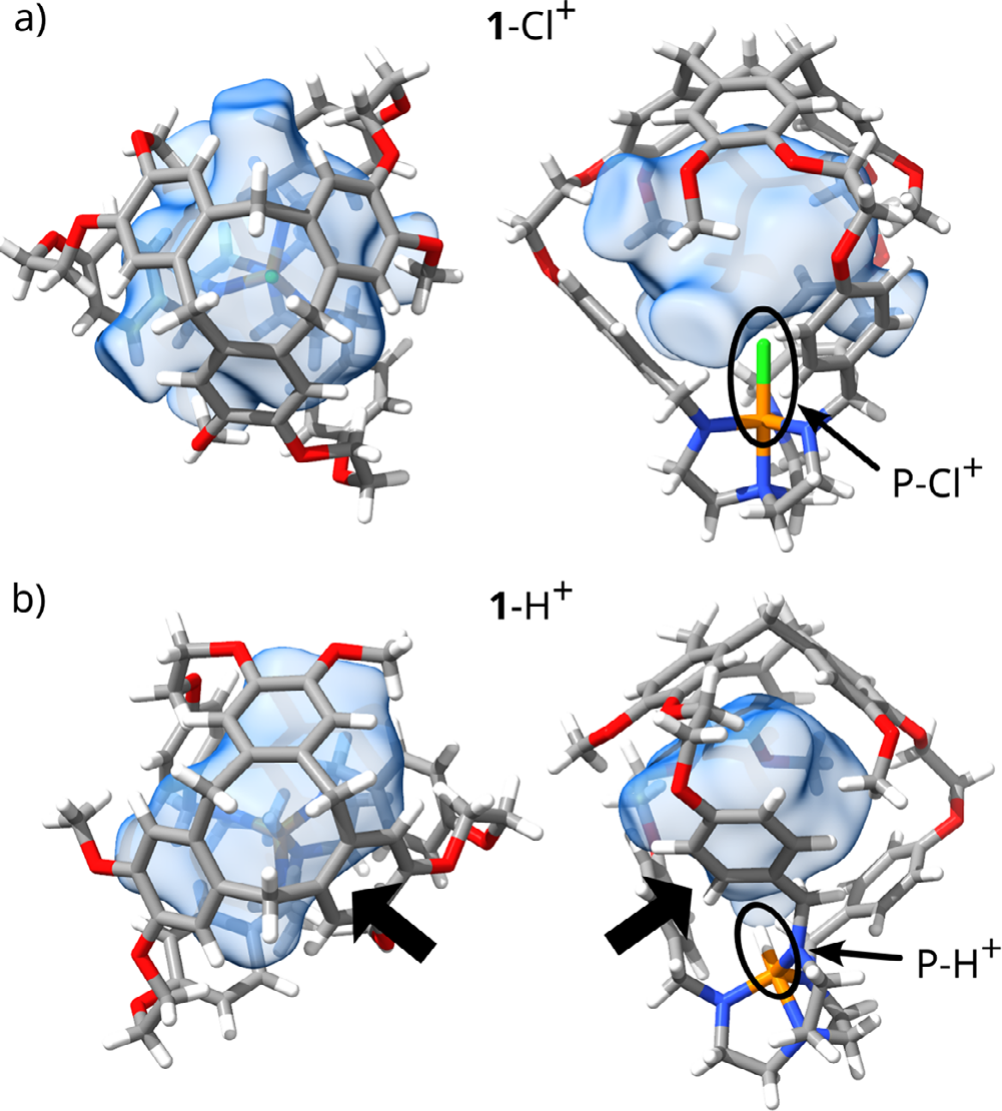
X‐ray structure of (a) **1−Cl^+^** and (b) **1−H^+^** (from [74] for this latter). Counter ions (Cl^−^) are omitted for clarity. Cavity shapes in blue calculated using MoloVol [76].

The structure of encaged chloroazaphosphatrane **1‐Cl^+^** was then compared to that of its parent azaphosphatrane **1‐H^+^** (Figure 2) [74]. In **1‐Cl^+^** the molecular cavity is occupied by a CHCl_3_ guest (72 Å^3^) and the chlorine atom of the P‐Cl motif. In **1‐H^+^** the cavity is occupied by a CH_2_Cl_2_ guest (58 Å^3^) and the hydrogen of the P‐H motif. Consequently, these two parent hemicryptophane complexes differ in both cavity size and shape (Figure 2): **1‐Cl^+^** presents a larger cavity (171 Å^3^) than **1‐H^+^** (117 Å^3^). Hemicryptophanes are known to be highly flexible [75], particularly through the OCH_2_CH_2_O conformations of the linkers. The presence of the P‐Cl motif and the CHCl_3_ guest molecule in the cavity of **1‐Cl^+^** leads to a more inflated structure than for the parent complex **1‐H^+^** with the P‐H motif and the smaller CH_2_Cl_2_ guest. Such a cavity modulation optimizes host‐guest interactions and is particularly evidenced by a slight increase of the axial P···CTV distance (measured between phosphorus atom and the plane defined by the three CH_2_ bridges of the CTV cap) in **1‐Cl^+^** vs **1‐H^+^** (10.06 Å vs 9.84 Å) and the width of the cavity estimated from the O···O averaged distances between the –PhOCH_2_– groups of the linkers (10.41 Å vs 9.37 Å). Consequently, the geometry of the inner space is strongly affected by this atom substitution: the **1‐Cl^+^** cage displays three windows connected to the cage interior, the **1‐H^+^** cage presenting only one accessible window. The switch from an endohedral P‐H^+^ hydrogen‐bond donor to a P‐Cl^+^ halogen‐bond donor is reflected in geometric and electronic differences that explain the distinct recognition properties of **1‐Cl^+^** and **1‐H^+^** in solution as described below.

First, we investigated the stability of the chloroazaphosphatrane confined in the well‐defined cavity of the hemicryptophane. Haloazaphosphatranes are indeed sensitive to weak nucleophiles such as water. Accordingly, to a solution of the cage **1‐Cl^+^·Cl^−^** or of the model compound **2‐Cl^+^·Cl^−^** in CD_2_Cl_2_ (5 mM, 0.5 mL), 100 µL of D_2_O were added. The quantity of chloroazaphosphatrane was then monitored by ^3^ ^1^P NMR spectroscopy over time (Figure S50). Figure 3 clearly shows that the confined chloroazaphosphatrane **1‐Cl^+^·Cl^−^** is significantly more stable than its model counterpart **2‐Cl^+^·Cl^−^**, as no degradation was observed over 15 h for the former, whereas a decomposition rate of 0.22 µmol.L^−^^1^.s^−^^1^ was observed for the latter. After 8 days, no detectable degradation of **1‐Cl^+^** was observed by NMR (Figure S53), allowing us to estimate an upper limit for its decomposition rate of 7 10^−4^ µmol.L^−1^.s^−1^. Hence, the encapsulation of the halogen‐bond donor unit protects it from degradation, likely due to the steric hindrance imposed by the cage, hampering nucleophilic attack by water.

**FIGURE 3:**
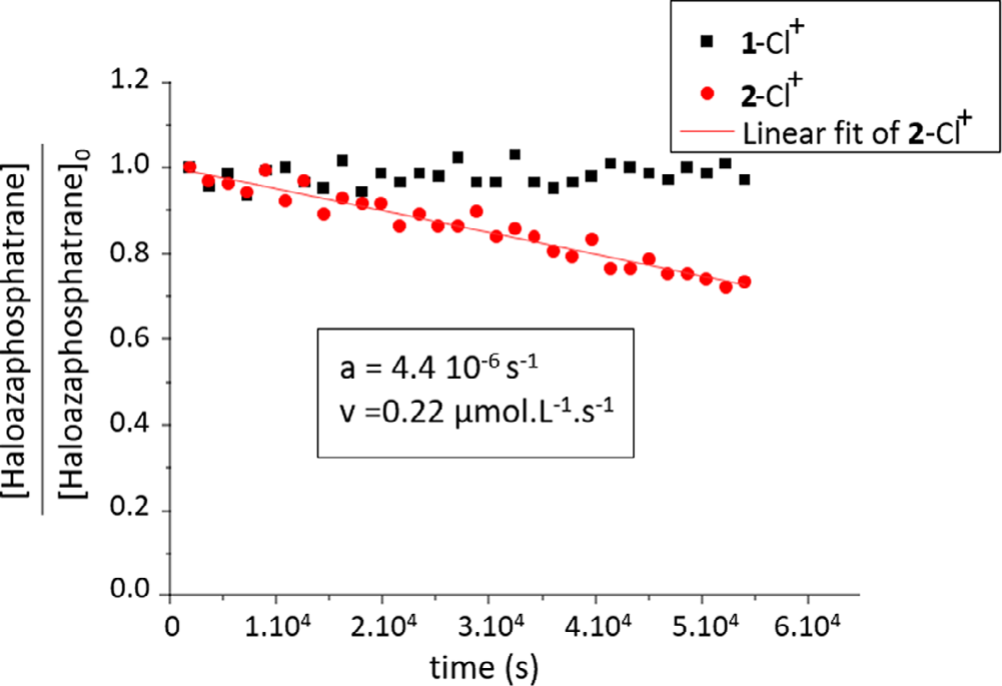
Comparison of the stability of the chloroazaphosphatranes **1‐Cl^+^** and **2‐Cl^+^** in the presence of water.

We next investigated the XB properties of the confined chloroazaphosphatrane in solution towards neutral Lewis bases. ^1^H NMR titrations were performed, and binding constants were determined by fitting the titration curves using Bindfit software (Figures S18–S44). Two sets of comparisons were carried out. Firstly, the binding properties of **1‐Cl^+^·BarF^–^** were compared with those of the model chloroazaphosphatrane **2‐Cl^+^·BarF^–^**, lacking the cavity, to assess the effect of confinement. Secondly, the analogous cavity containing an HB donor instead of an XB donor (**1‐H^+^·BarF^−^**) was examined to evaluate the specific contribution of the XB motif.

The confined space around the XB motif was found to strongly affect its binding properties toward various guests (Figure 4). Although small in magnitude, chemical shift changes follow very clear monotonous trends with the addition of host and are fully consistent with recognition phenomena involving 1:1 host:guest binding. For pyridine, the association constant is four times higher with the cage **1‐Cl^+^·BarF^–^** than with its open counterpart **2‐Cl^+^·BarF^–^** (*K_a_* of 204 M^−1^ and 51 M^−1^ respectively, Figure 4). The tertiary urea (tetramethylurea) is complexed with an association constant of 1310 M^−1^ by **1‐Cl^+^·BarF^–^**, whereas no complexation is observed with either **2‐Cl^+^·BarF^–^** or **1‐H^+^·BarF^–^**. Switching from urea to thiourea results in a decrease of binding for the cage but allows measurable complexation with the open model **2‐Cl^+^·BarF^–^**. However, the binding constant remains eleven times higher with **1‐Cl^+^·BarF^–^** than with the model (*K_a_* = 221 M^−1^ and 20 M^−1^ respectively, Figure 4). The improvement of the binding constant, when using thiourea instead of urea, observed with the model **2‐Cl^+^·BarF^–^** is in agreement with what is usually achieved with other XB motifs. The lower affinity of the cage **1‐Cl^+^·BarF^–^** for thiourea guest (compared to urea guest) highlights the specific behavior of this receptor. The bigger size of the sulfur atom could induce some electronic repulsions of its lone pairs with the electron‐rich aromatic rings of the walls. Using TEPO as a guest further highlights the effect of confinement: the association of the **1‐Cl^+^·BarF^–^** is around one hundred times higher than that of **2‐Cl^+^·BarF^–^** (*K_a_* = 4321 M^−1^ and 46 M^−1^ respectively). In contrast, the cage **1‐H^+^·BarF^–^**, devoid of XB donor motif, displays no recognition properties of these neutral guests. Thus, both the endohedral XB motif and the geometric control imposed by the cavity are important factors for improving complexation by halogen bonding. Finally, **1‐Cl^+^·BarF^–^** binds TPPO with an association constant approximately ten times lower than that measured for TEPO, further supporting an endohedral recognition process. The greater steric demand of TPPO likely hinders its access to the encapsulated XB donor site within the cavity.

**FIGURE 4:**
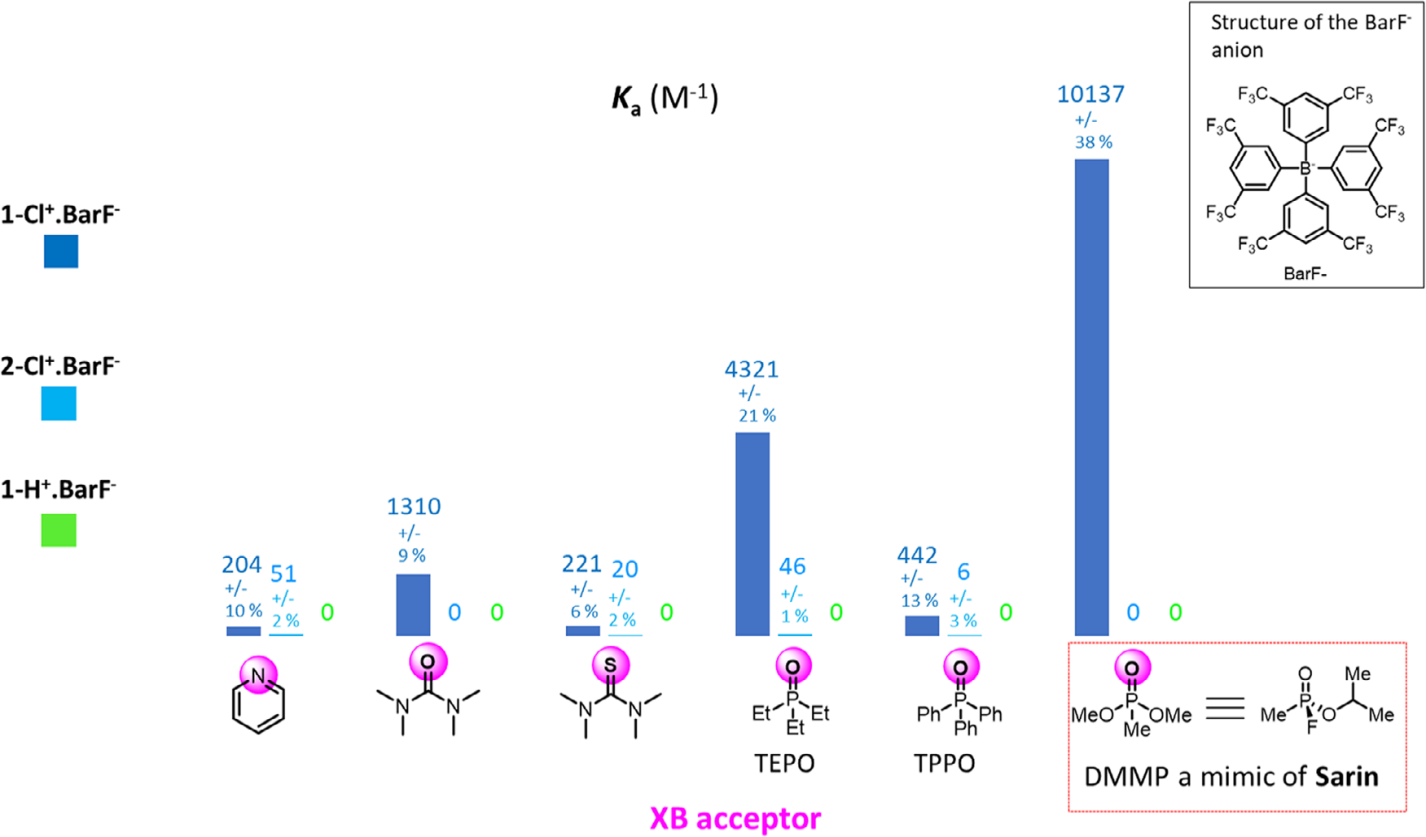
Association constants of **1‐Cl^+^^.^BarF^–^**, **2‐Cl^+^^.^BarF^–^, 1‐H^+^^.^BarF^–^** with different neutral Lewis bases. *Ka* (M^−1^) were obtained by NMR titration experiments (CD_2_Cl_2_, 500 MHz, 298K).

The ability of cage **1‐Cl^+^·BarF^–^** to encapsulate TEPO prompted us to examine its potential for binding dimethyl methylphosphonate (DMMP), a surrogate of the extremely neurotoxic compound so‐called Sarin. Indeed, concave supramolecular hosts appear as a promising class of selective receptors for toxic tetrahedral organophosphorus compounds and could lead to an easy detection or removal of nerve agents from biological systems. For instance, Badjić et al. reported that the shape of their *C_3_* symmetric molecular baskets was nicely complementary to tetrahedral organophosphorus guest leading to the efficient and selective complexation of nerve agents [77, 78, 79]. When DMMP was used as the guest, a high binding constant of 10^4^ M^−1^ was obtained with **1‐Cl^+^·BarF^–^** whereas no recognition was observed for both **2‐Cl^+^·BarF^–^** and **1‐H^+^·BarF^–^**. This result shows that a simple atom substitution on the phosphorus of the cage enables the occurrence of an efficient recognition by halogen bonding with atomic precision. The shielding of the P–OCH_3_ protons relative to the P–CH_3_ protons upon complexation suggests that one methoxy group occupies the aromatic cavity of the CTV unit (Figure S42). Variable‐temperature NMR titrations were performed to investigate the thermodynamic origin of DMMP binding (Figure S47 and Table S1). A ΔH value of +9 kJ mol^−^^1^ and a ΔS value of +107 J mol^−^^1^ K^−^^1^ were determined, indicating that the complexation process is entropically driven.

DOSY NMR spectroscopy experiments were performed in CD_2_Cl_2_ and support the formation of a DMMP@**1‐Cl^+^·BarF^–^** complex (Figures S45 and S46). Under fast exchange conditions on the NMR timescale, the measured self‐diffusion coefficient of DMMP decreases from 2.52 × 10^−^^9^ m^2^ s^−^^1^ to 1.89 × 10^−^^9^ m^2^s^−^^1^ in the presence of one equivalent of host **1‐Cl^+^·BarF^–^**. This decrease reflects the partial encapsulation of the guest within the host cavity. Notably, the diffusion coefficient does not reach that of the host (*D* = 0.85 × 10^−^^9^ m^2^s^−^^1^), which is consistent with a fast exchange regime.

Furthermore, the structure of the DMMP@**1‐Cl^+^·BarF^–^** complex was investigated by DFT calculations. The optimized geometry reveals that DMMP is fully encapsulated within the hemicryptophane cavity (Figure 5). Notably, a halogen bond is observed between the chlorine atom of the haloazaphosphatrane moiety and one oxygen atom of a methoxy group of DMMP, with a P–Cl···O distance of 2.88 Å and a P–Cl···O angle of 161°. Additional CH···π interactions are also identified: the methyl group and one methoxy unit interact with the CTV unit of the cage (H···C distances ranging from 2.42 to 2.67 Å), and one aromatic ring of the linkers (H···C distance of 2.68 Å) respectively. These observations are consistent with the deshielding of the corresponding guest protons observed during titration experiments; at low guest concentrations, the shielding effect of the host's aromatic rings is more pronounced. Moreover, a hydrogen bond is detected between a hydrogen atom of the OCH_2_CH_2_O linker fragment and the oxygen atom of the P = O group, with a very short CH···O distance of 2.2 Å. Overall, the high binding constant measured for DMMP with host **1‐Cl^+^·BarF^–^** arises from multiple cooperative interactions. The halogen bond likely increases the effective electronegativity of the coordinated oxygen atom. As a consequence, the partial positive charge (δ^+^) on the hydrogen atoms of the methyl groups attached to phosphorus and to this oxygen atom would be enhanced, potentially leading to stronger CH–π interactions with the CTV units and the aromatic walls of the cage. In addition, a more favorable arrangement of the halogen‐bond donor and acceptor, promoted by additional host–guest interactions within the cavity, probably also contribute to the enhanced binding.

**FIGURE 5:**
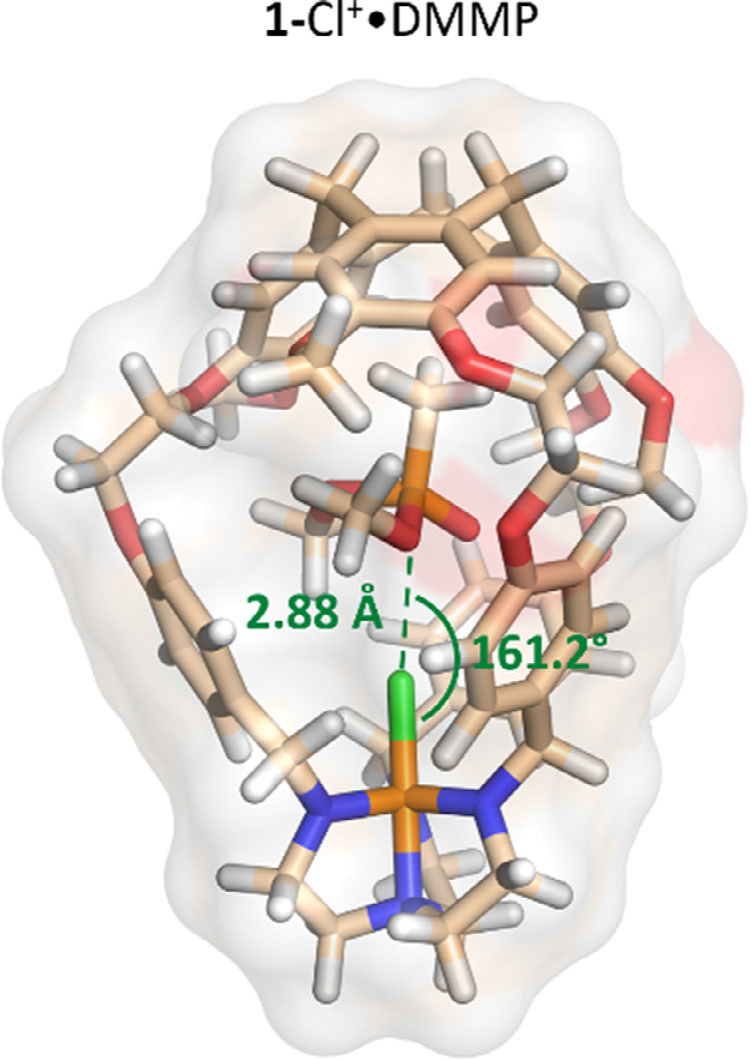
DFT optimized structure showing the encapsulation of DMMP in hemicryptophane **1‐Cl^+^·BarF^–^**.

Consistently with these observations, a cavity volume of 206 Å^3^ was calculated for the cage extracted from the DFT‐optimized geometry of the DMMP@**1‐Cl^+^** complex, after removal of the guest. This corresponds to a packing coefficient (PC) of 53%, which falls within the optimal range defined by Rebek's criterion for host–guest complementarity [80]. Notably, this value is significantly larger than that determined from the X‐ray crystal structure obtained with chloroform as a guest molecule, suggesting that the cavity undergoes adaptive conformational changes to accommodate the size and shape of the encapsulated guest. Such flexibility enables the establishment of favorable interactions with both the northern and southern regions of the cavity. A PC value of 50% was also calculated for the hypothetical DMMP@**1‐H^+^** complex, using the same procedure. The two PC values are very similar and both are in agreement with the Rebek criterion [80], suggesting that the significant difference in binding constants is most likely due to the halogen‐bond donor unit present in **1‐Cl^+^**.

In conclusion, this work describes a strategy to enhance halogen bonding interactions through confinement. Endohedral functionalization of a molecular cage with an XB motif allows control over the conformation and orientation of the interacting partners. As a result, binding constants are significantly higher than those observed for either the model XB donor lacking a cavity or the analogous cavity containing a hydrogen‐bond donor instead. These latter receptors show negligible or no recognition for the neutral guests studied, whereas the confined XB motif efficiently complexes various XB acceptors. Cooperative interactions between the XB donor motif and the other functions of the cage can also account for this result. Finally, the receptor was also shown to bind DMMP, a Sarin surrogate, by halogen bonding and DFT calculations show that the cavity geometry enforces favorable alignment for halogen bonding, thereby promoting this interaction. These results both show the relevance of the approach toward finely tuning and improving halogen bonding, and its amenability toward inwardly positioned multi‐XB donor cages.

## Author Contributions

**Pierre‐Antoine Dupin**: investigation, methodology, validation, formal analysis, writing – review and editing. **Jian Yang**: investigation, validation, methodology, formal analysis, writing – review and editing. **Elise Antonetti**: investigation, methodology, formal analysis, writing – review and editing, validation. **Bastien Chatelet**: validation, formal analysis, supervision, writing – review and editing. **Xavier Bugaut**: validation, formal analysis, supervision. **Jean‐Pierre Dutasta**: writing – review and editing. **Paola Nava**: formal analysis, validation, investigation, writing – review and editing, funding acquisition. **Anne‐Doriane Manick**: investigation, validation, formal analysis, supervision, writing – review and editing, methodology. **David Bardelang**: validation, formal analysis, supervision, writing – review and editing. **Alexandre Martinez**: conceptualization, funding acquisition, writing – original draft, writing – review and editing, project administration, supervision, validation.

## Conflicts of Interest

The authors declare no conflicts of interest.

## Supporting information



Additional experimental details, materials and methods, procedures, NMR spectra of prepared compounds, compound characterization data, host‐guest supramolecular complexes information, X‐ray data and computational details. The authors have cited additional references within the Supporting Information [81, 82, 83, 84, 85, 86, 87, 88, 89].**Supporting File**: anie74981‐sup‐0001‐SuppMat.pdf.

## Data Availability

The data that support the findings of this study are available from the corresponding author upon reasonable request.
