# Supplementary material for: Molecular Recognition by Halogen Bonding With Atomic Precision in a Covalent Organic Cage
Source: Angew Chem Int Ed Engl. 2026 Sep 27;65(45):e5640364. doi: 10.1002/anie.5640364 (PMC13725614; doi:10.1002/anie.5640364)
Supplement: Supplementary file 1 [file ANIE-65-e5640364-s001.pdf]

Supporting information

Molecular Recognition by Halogen Bonding with Atomic Precision in a Covalent Organic Cage

Pierre-Antoine Dupin,[a] Jian Yang,*[b] Elise Antonetti,[a] Bastien Chatelet,[c] Xavier Bugaut,[d] Jean-Pierre Dutasta,[e] Paola Nava,[a] Anne-Doriane Manick,*[f] David Bardelang,*[a] and Alexandre Martinez*[a]

I/ General Information 2
II/ Experimental section 3
III/ NMR spectra 5
IV/ Mass spectra 12
V/ Crystallographic data 13
VI/ ^{1}H NMR titrations 15
VII/ Variable-temperature ^{1}H NMR titrations 32
VIII/ DOSY experiments 33
IX/ Stability studies 34
X/ Computational approach 37

I/ General Information

Commercial reagents were used directly as received without further purification. Dichloromethane was dried prior to use through standard procedures or obtained from a solvent drying system (MB-SPS-800). ^{1}H and ^{13}C NMR spectra were recorded on a Brucker AC 300, Brucker AC 400, Bruker 500 HD spectrometers. Chemical shifts were reported in ppm on the δ scale relative to residual $CDCl_3$ (δ = 7.26 for ^{1}H NMR and δ = 77.16 for ^{13}C NMR), CD_2Cl_2 (δ = 5.32 for ^{1}H NMR and 53.84 for ^{13}C NMR) as the internal references. Coupling constant (*J*) are reported in Hertz unit (Hz). Multiplicities are described with standard following abbreviations: s = singlet, br = broad, d = doublet, t = triplet, q = quadruplet, m = multiplet. Column chromatographies were performed with gel 60 (Macherey-Nagel® Si 60, 0.040-0.063 mm). Analytical thin layer chromatography (TLCs) was carried out on Merck®Kieselgel 60 F254 plates and achieved under a 254 nm UV light. High-resolution mass spectra (HRMS) were performed on a SYNAPT G2 HDMS (Waters) spectrometer equipped with atmospheric pressure ionization source (API) pneumatically assisted.

II/ Experimental section

Compounds **1-H$^+$.Cl$^-$** and **2-Cl$^+$.BArF$^-$** were obtained according to our previous work.[1,2][3]

Azaphosphatrane 1-H$^+$.BArF$^-$

In a flame-dried Schlenk flask were added dried dichloromethane (2.2 mL), and **1-H$^+$.Cl$^-$** (50 mg, 0.048 mmol). NaBArF (42 mg, 0.048 mmol) was added dropwise, and the mixture was stirred overnight at room temperature. The solvent was evaporated under vacuum and dried acetone (2 mL) was added. A white precipitate was observed. The supernatant was evaporated under vacuum to give pure **1-H$^+$. BArF$^-$** (88 mg, 98%). T_m : 156-157 °C . IR: cm^{-1}
3387, 2864, 2143, 1931, 1608, 1514, 1452, 1355, 1316, 1206, 1204, 1108, 1038, 998, 737, 621, 544, 536. HRMS (ESI): m/z [M]$^+$ calcd for $C_{57}H_{64}N_4O_9P^+$, 979.4405; found at 979.4403; ^{1}H NMR (500 MHz, CD_2Cl_2) δ = 7.72 (brs, 8H), 7.56 (s, 4H), 7.01 (s, 3H), 6.86 (s, 3H), 6.48 (d, *J* = 8.8 Hz, 6H), 6.44 (d, *J* = 8.8 Hz, 6H), 4.86 (d, *J* = 410 Hz, 1H), 4.77 (d, *J* = 13.75 Hz, 3H), 4.56-4.50 (m, 3H), 4.38-4.31 (m, 3H), 4.29-4.21 (m, 6H), 3.65 (s, 9H), 3.57 (dd, *J* = 13.0 Hz, 15.5 Hz, 3H), 3.55 (d, *J* = 13.8 Hz, 3H), 3.28-3.12 (m, 12H), 3.10-3.02 (m, 3H); ^{13}C NMR (75 MHz, CD_2Cl_2) δ = 157.3, 148.8, 147.2, 135.2, 132.9, 132.7, 130.3, 127.0, 126.8, 123.2, 117.8, 115.8, 115.8, 66.6, 66.3, 57.3, 54.5, 47.9 (d, *J* = 9.2 Hz), 46.2 (d, *J* = 7.1 Hz), 36.4; ^{31}P CPD NMR (160 MHz, CD_2Cl_2) δ = -25.21; ^{19}F NMR (376 MHz, CD_2Cl_2) δ = -62.88.

Chloroazaphosphatrane 1-Cl$^+$.Cl$^-$

Under an atmosphere of argon, in a flame-dried Schlenk flask, hemicryptophane azaphosphatrane **3-H$^+$.Cl$^-$** (310 mg, 0.31 mmol) was dissolved in dried dichloromethane (1.7 mL) *t*-BuOK (86 mg, 0.76 mmol) was added, and the reaction mixture was stirred at room temperature for 2 h. Then the solvent was removed under vacuum, and anhydrous toluene (3.4 mL) was added. The reaction mixture was stirred for another 0.5 h, and then the suspension was filtered under argon through a two-necked fritted glass funnel, then the solvent was removed under vacuum to give pure hemicryptophane proazaphosphatrane **5**. The product was directly used for the following step. To a flame-dried Schlenk tube was added freshly prepared hemicryptophane proazaphosphatrane **5**, dried dichloromethane (1.6 mL), and hexachloroethane (74 mg, 0.31 mmol); the mixture was stirred under argon at room temperature for 2 h. The solvent was evaporated under vacuum and the residue was washed three times by Et_2O to give the desired product **1-Cl$^+$.Cl$^-$** (196 mg, 60%). T_m : 162-163 °C . IR: cm^{-1} 3361, 3327, 2961, 2870, 1609, 1585, 1510, 1455, 1398, 1347, 1261, 1180, 1091, 948, 903, 869, 737. HRMS (ESI): m/z [M]$^+$ calcd for $C_{57}H_{63}ClN_4O_9P^+$, 1013.4016; found at 1013.4012; ^{1}H NMR (300

---

[1] P. Dimitrov Raytchev, A. Martinez, H. Gornitzka, J.-P. Dutasta, "Encaging the Verkade's Superbases: Thermodynamic and Kinetic Consequences", *Journal of the American Chemical Society* **133**, (2011): 2157–2159.

[2] P. D. Raytchev, O. Perraud, C. Aronica, A. Martinez, J. P. Dutasta, "A New Class of C_3-Symmetrical Hemicryptophane Hosts: Triamide- and Tren-Hemicryptophanes" *Journal of Organic Chemistry* **75**, (2010): 2099–2102.

[3] C. Li, A.-D. Manick, J. Yang, D. Givaudan, B. Biletskyi, S. Michaud-Chevalier, J.-P. Dutasta, D. Hérault, X. Bugaut, B. Chatelet, A. Martinez, "The Chloroazaphosphatrane Motif for Halogen Bonding in Solution", *Inorganic Chemistry* **60**, (2021): 11964–11973

MHz, CD_2Cl_2) δ = 6.89 (d, *J* = 8.6 Hz, 6H), 6.74 (d, *J* = 6.1 Hz, 6H), 6.74-6.68 (m, 6H), 4.64 (d, *J* = 13.8 Hz, 3H), 4.48 (d, *J* = 13.8 Hz, 3H), 4.42-4.32 (m, 6H), 4.27-4.07 (m, 6H), 4.07-3.92 (m, 3H), 3.71 (s, 9H), 3.65-3.49 (m, 9H), 3.44 (d, *J* = 13.8 Hz, 3H), 3.43 (dd, *J* = 6.9 Hz, 14.0 Hz, 3H); ^{13}C NMR (75 MHz, CD_2Cl_2) δ = 156.7, 148.8, 147.4, 132.9, 132.7, 131.6, 126.8, 116.2, 115.9, 115.5, 66.5, 66.2, 57.5, 54.6, 45.9 (d, *J* = 9.4 Hz), 45.5 (d, *J* = 8.9 Hz), 36.4; ^{31}P CPD NMR (121 MHz, CD_2Cl_2) δ = -20.44.

Chloroazaphosphatrane 1-Cl$^+$.BArF$^-$

In a flame-dried Schlenk flask were added dried dichloromethane (1.0 mL), and **1-Cl$^+$.Cl$^-$** (20 mg, 0.019 mmol). NaBArF (17 mg, 0.019 mmol) was added dropwise, and the mixture was stirred overnight at room temperature. The solvent was evaporated under vacuum and dried acetone (1.0 mL) was added. A white solid precipitate was observed. The supernatant was evaporated under vacuum to give pure **1-Cl$^+$. BArF$^-$** (21 mg, 92%). T_m : 124-125 °C . IR: cm^{-1}
2936, 1610, 1512, 1457, 1355, 1277, 1222, 1124, 1092, 887, 839, 713, 682, 670. HRMS (ESI): m/z [M]$^+$ calcd for $C_{57}H_{63}ClN_4O_9P^+$, 1013.4016; found at 1013.4012; ^{1}H NMR (500 MHz, CD_2Cl_2) δ = 7.72 (t, *J* = 2.73 Hz, 8H), 7.56 (s, 4H), 6.88 (d, *J* = 8.7 Hz, 6H), 6.77 (d, *J* = 5.7 Hz, 6H), 6.73 (d, *J* = 7.9 Hz, 6H), 4.66 (d, *J* = 13.8 Hz, 3H), 4.47-4.32 (m, 9H), 4.27 (dt, *J* = 4.0 Hz, 11.1 Hz, 3H), 4.19-4.11 (m, 3H), 4.11-3.97 (m, 3H), 3.72 (s, 9H), 3.56-3.33 (m, 6H), 3.46 (d, *J* = 13.8 Hz, 3H), 3.32-3.19 (m, 3H), 3.10-2.97 (m, 3H); ^{13}C NMR (75 MHz, CD_2Cl_2) δ = 157.2, 148.7, 147.1, 135.2, 133.0, 132.7, 130.3, 130.3, 129.0, 127.0, 126.8, 123.2, 117.9, 115.8, 115.7, 66.7, 66.4, 57.3, 54.6, 47.9 (d, *J* = 7.8 Hz), 46.2 (d, *J* = 7.2 Hz), 36.4; ^{31}P CPD NMR (121 MHz, CD_2Cl_2) δ = -7.71; ^{19}F NMR (376 MHz, CD_2Cl_2) δ = -62.88.

III/ NMR spectra

Azaphosphatrane 1-H⁺.BArF⁻

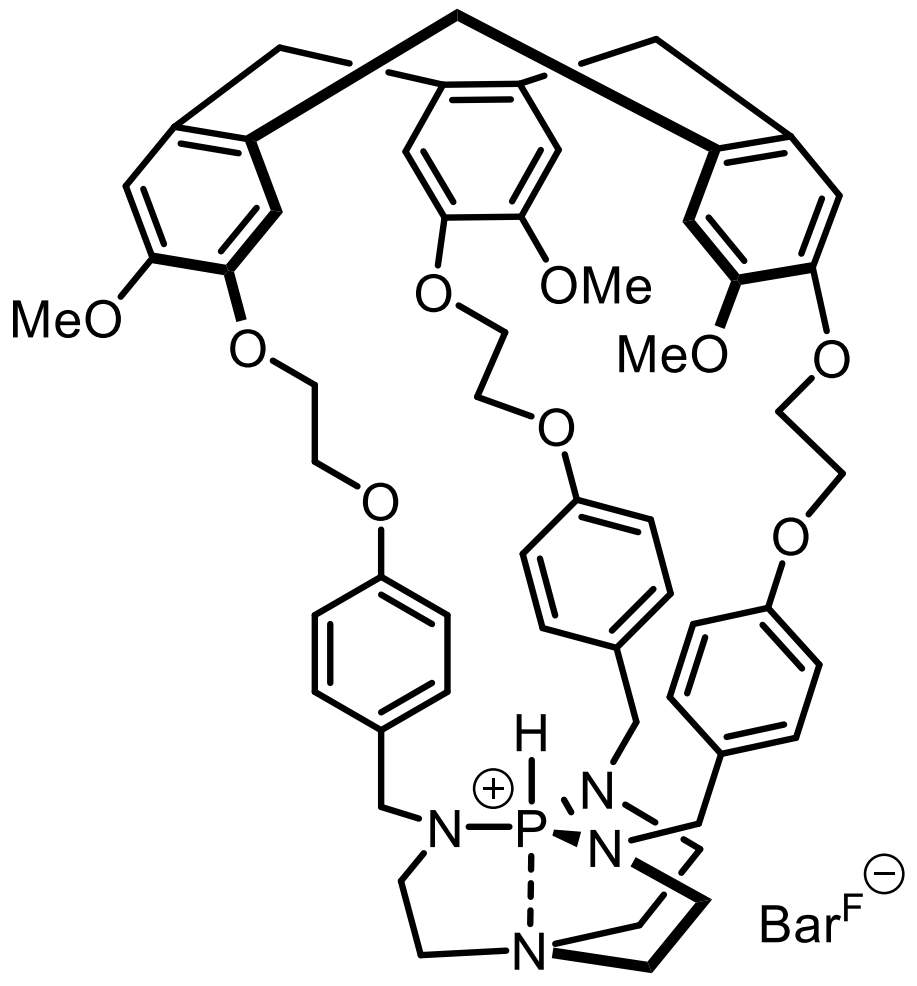

1-H⁺.Bar^F-

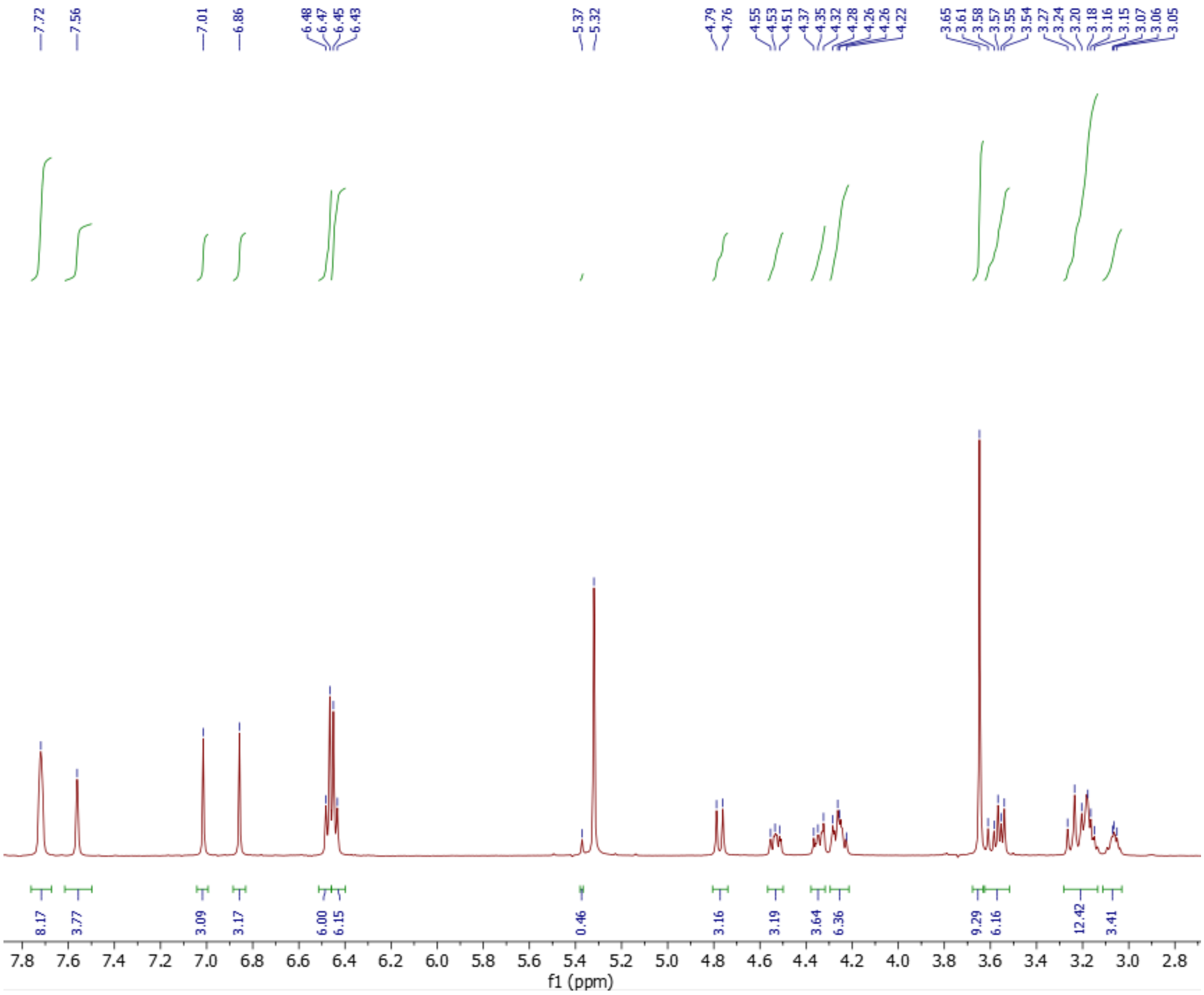

Figure S1 : ^{1}H NMR (500 MHz, CD_2Cl_2) spectrum of **1-H⁺.BArF**

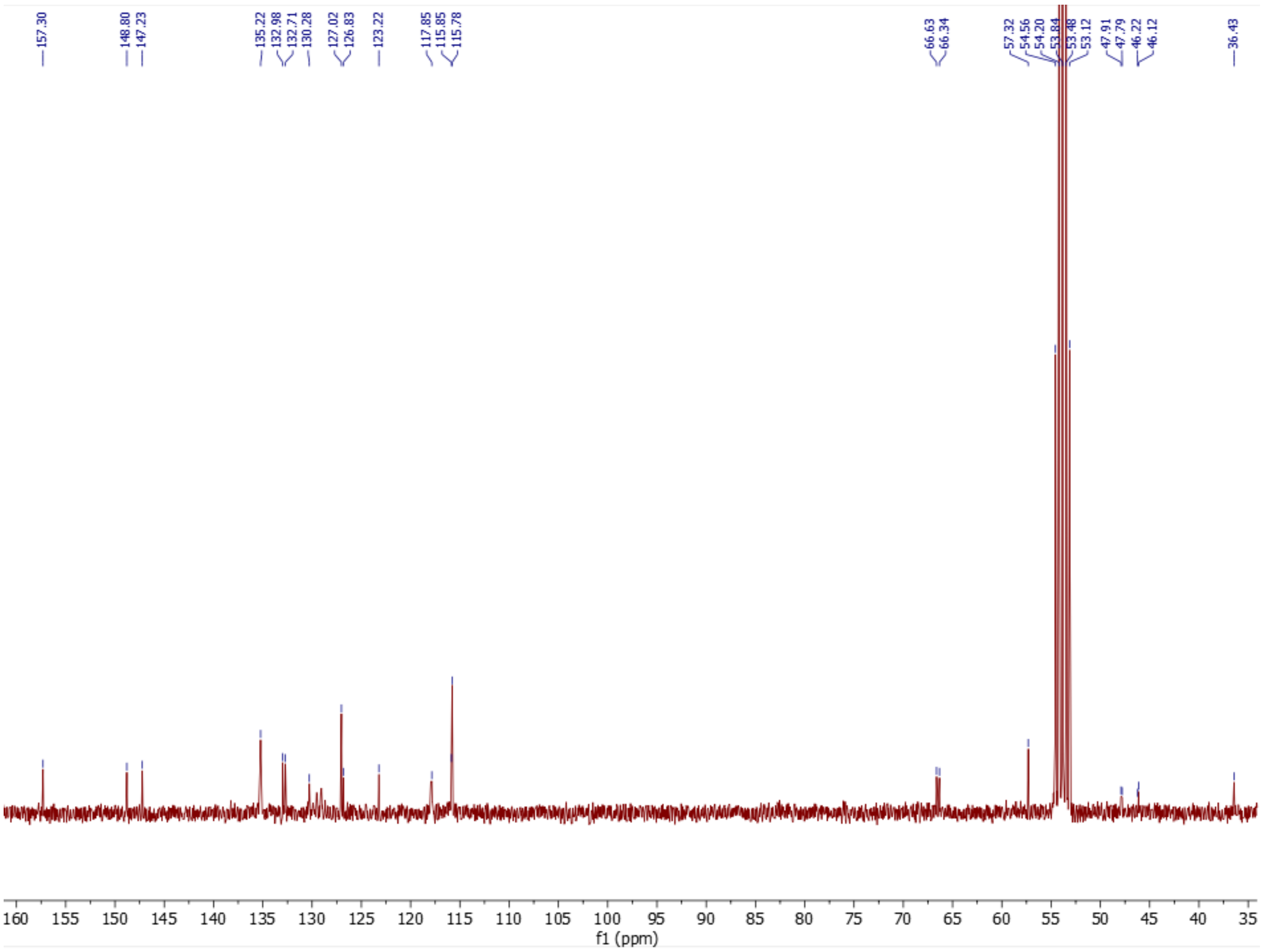

Figure S2 : ^{13}C NMR (75 MHz, CD_2Cl_2) spectrum of **1-H$^+$.BArF$^-$**

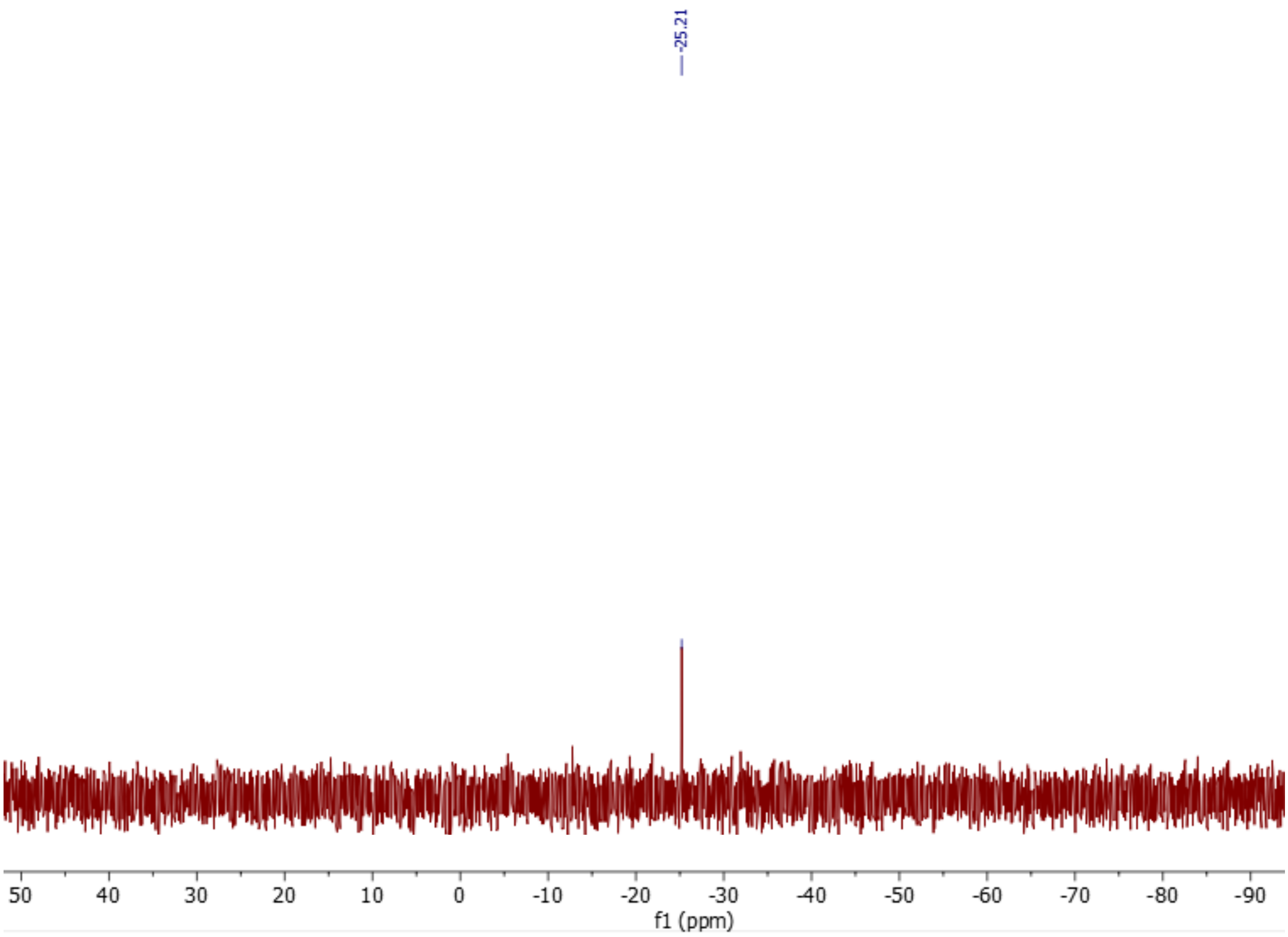

Figure S3 : ^{31}P CPD NMR (160 MHz, $CDCl_3$) spectrum of **1-H$^+$.BArF$^-$**

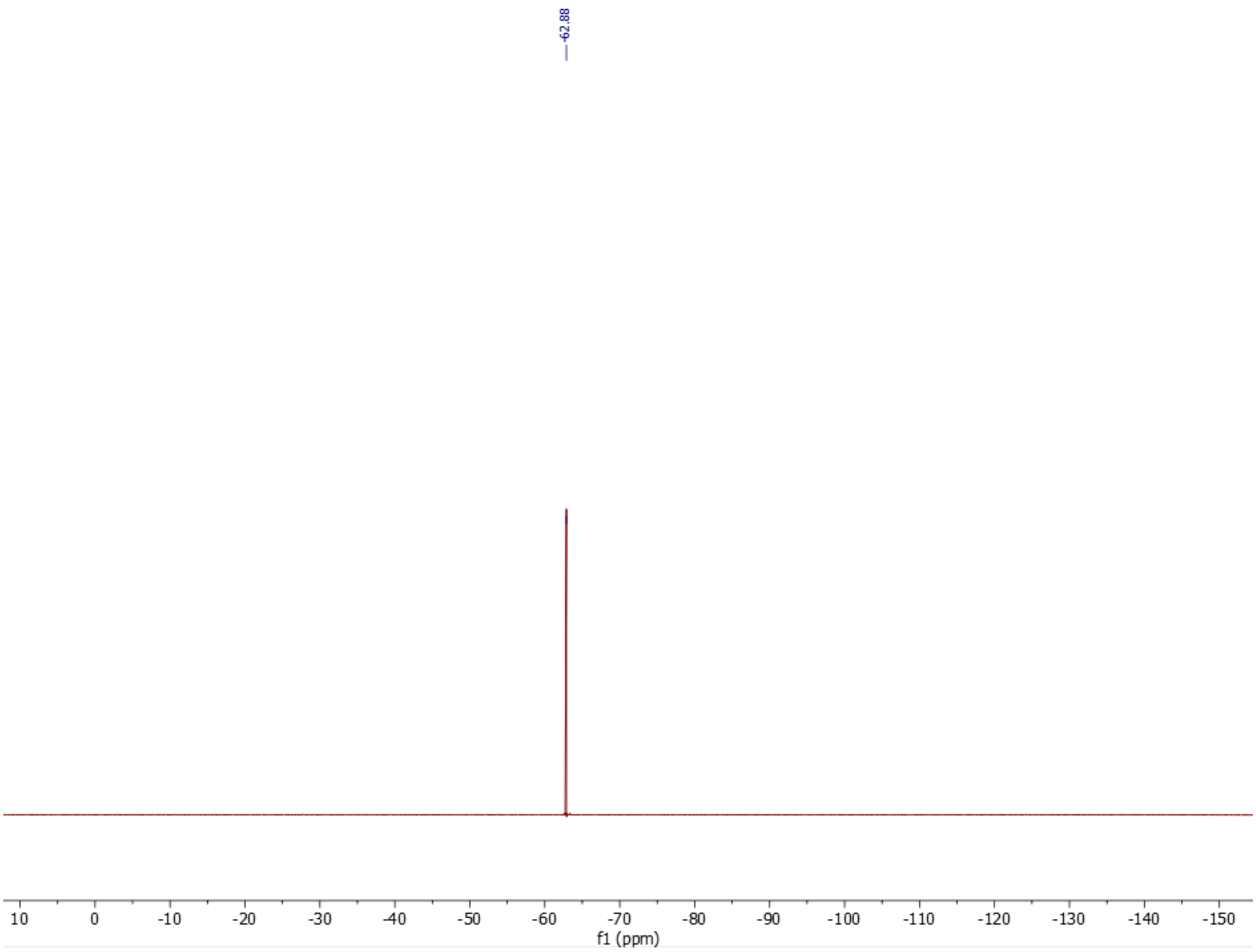

Figure S4 : ^{19}F NMR (376 MHz, CD_2Cl_2) spectrum of **1-H$^+$.BArF$^-$**

Chloroazaphosphatrane 1-Cl$^+$.Cl$^-$

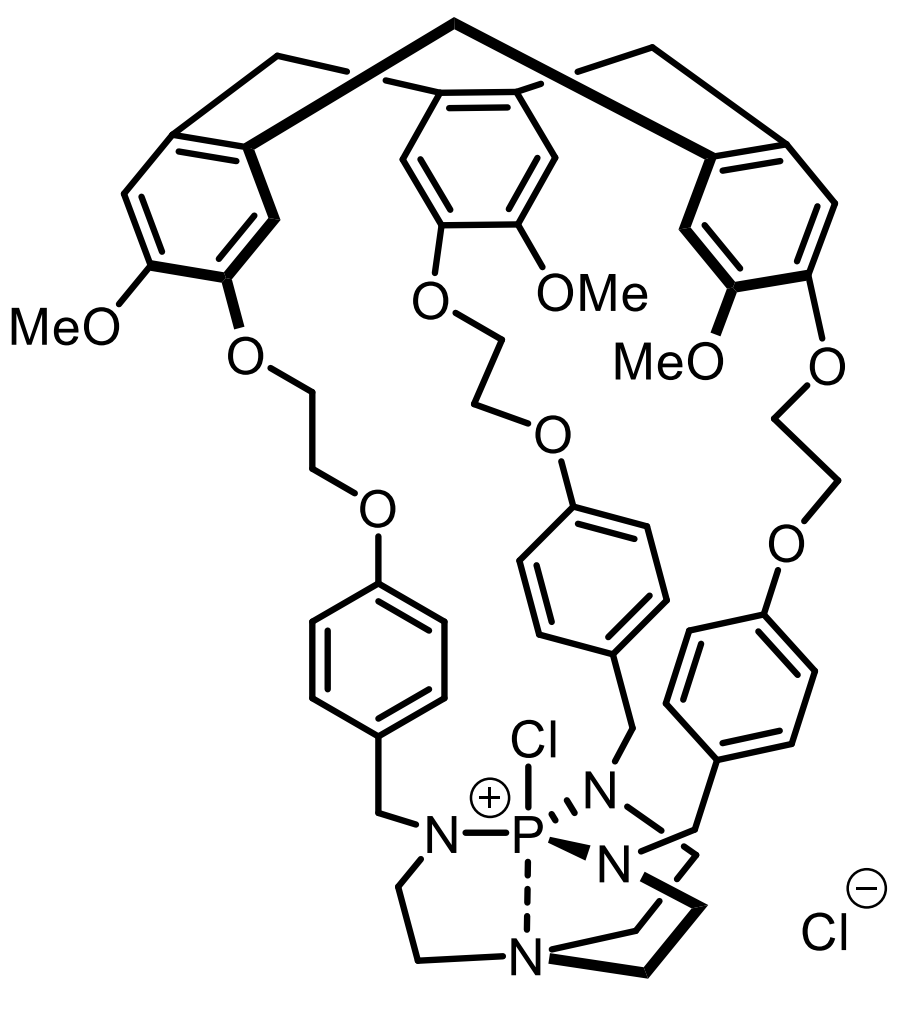

1-Cl$^+$.Cl$^-$

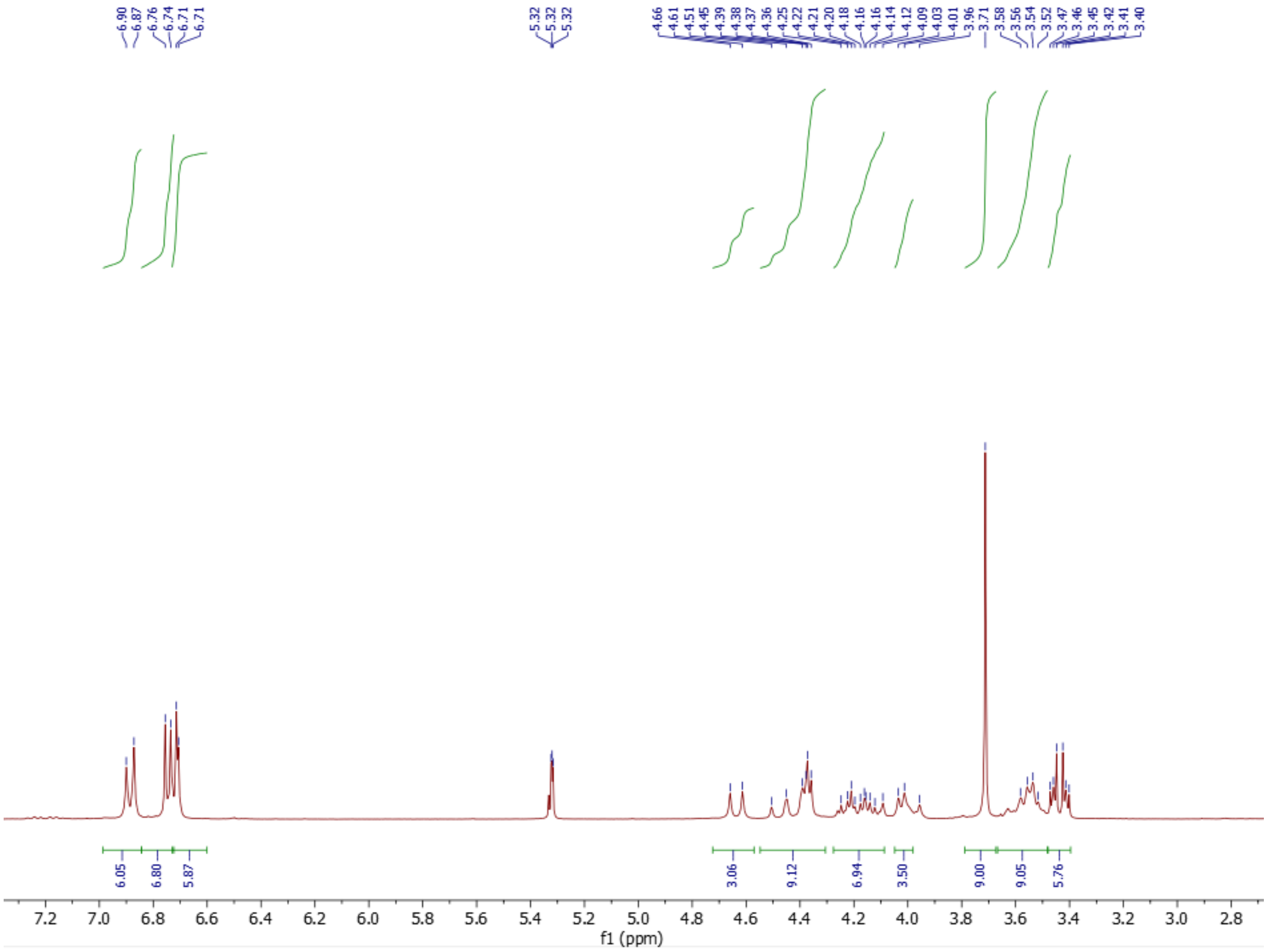

Figure S5 : ^{1}H NMR (300 MHz, CD_2Cl_2) spectrum of **1-Cl^{+}.Cl^{-}**

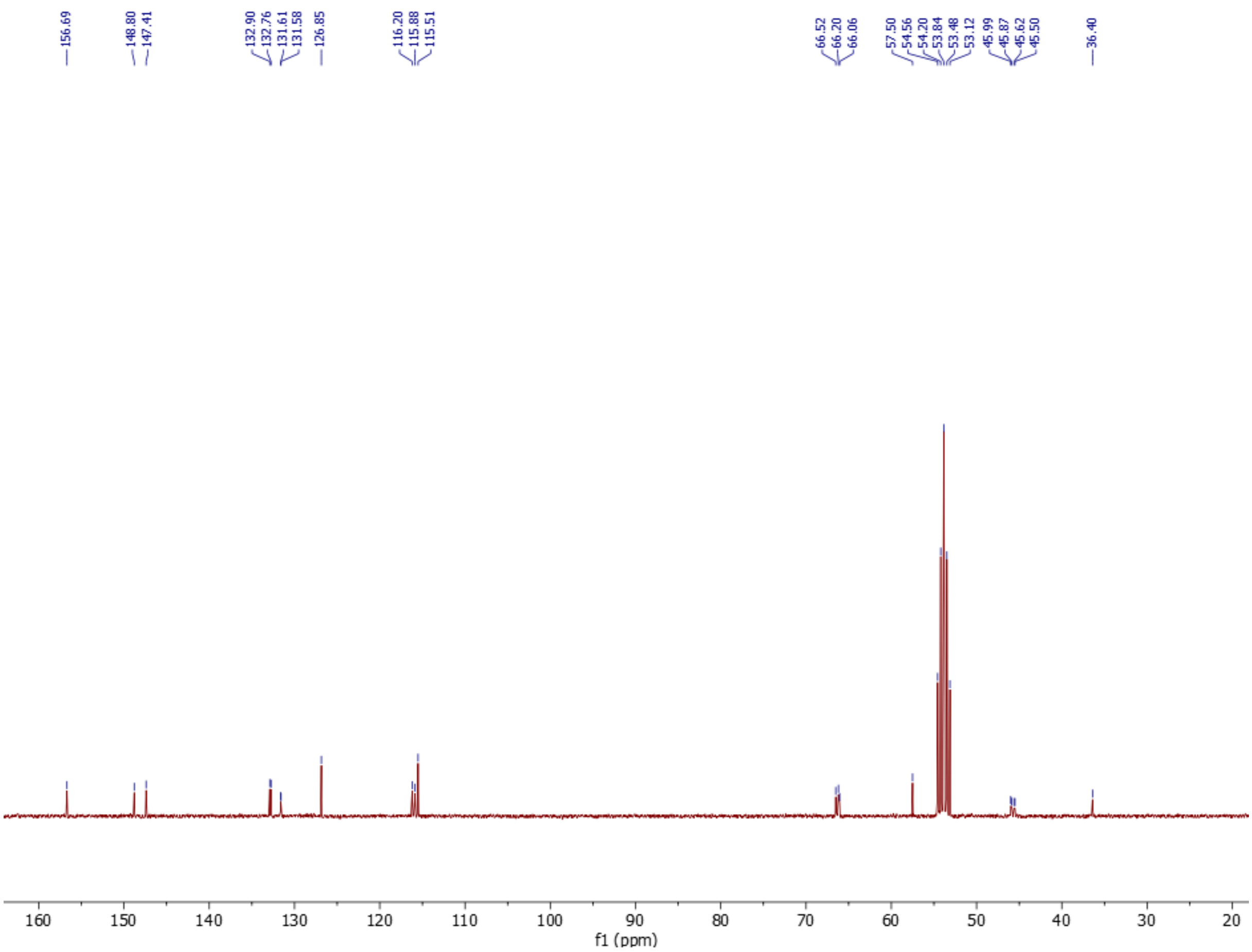

Figure S6 : ^{13}C NMR (75 MHz, CD_2Cl_2) spectrum of **1-Cl^{+}.Cl^{-}**

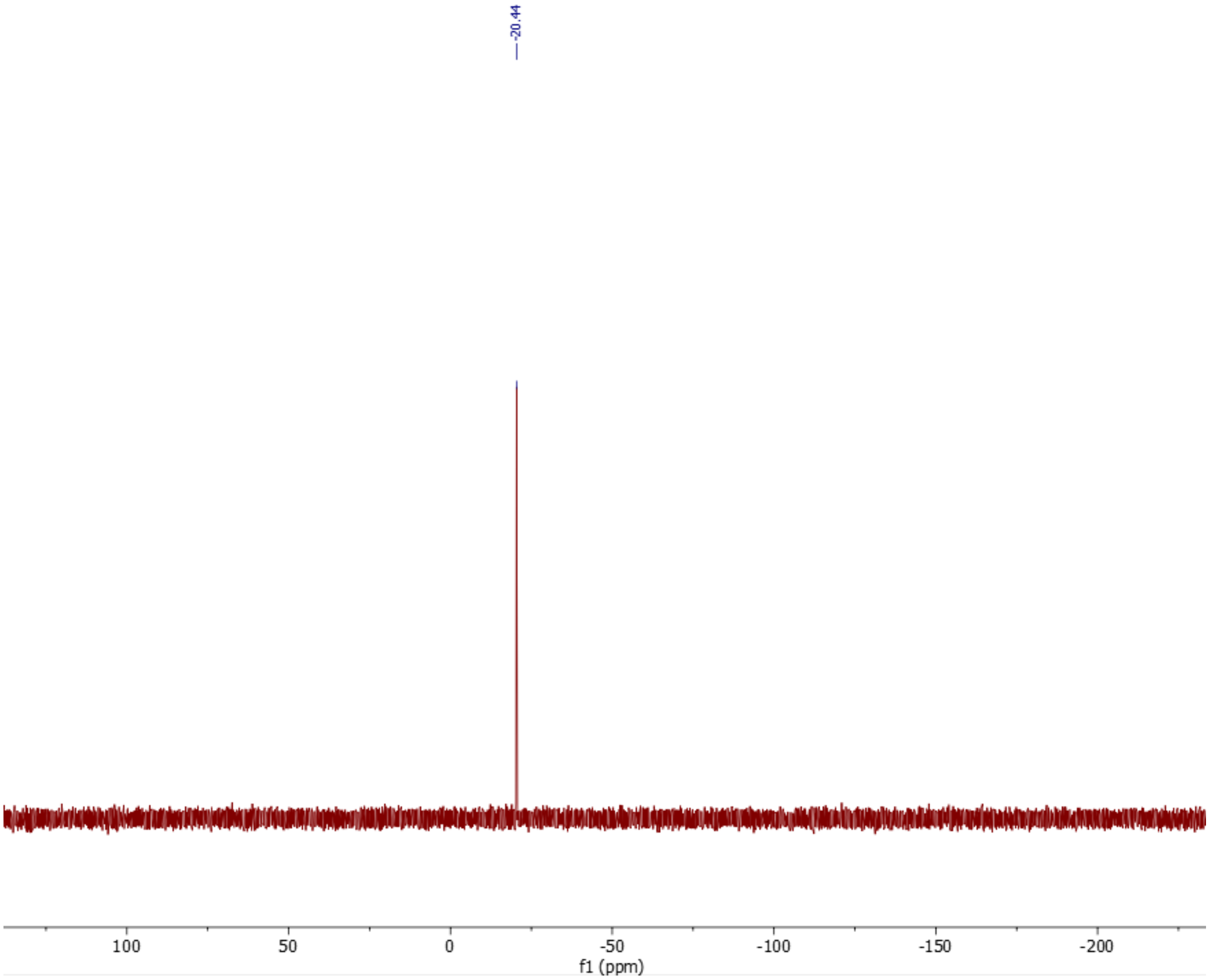

Figure S7 : ^{31}P CPD NMR (121 MHz, CD_2Cl_2) spectrum of **1-Cl$^+$.Cl$^-$**

Chloroazaphosphatrane 1-Cl$^+$.BArF$^-$

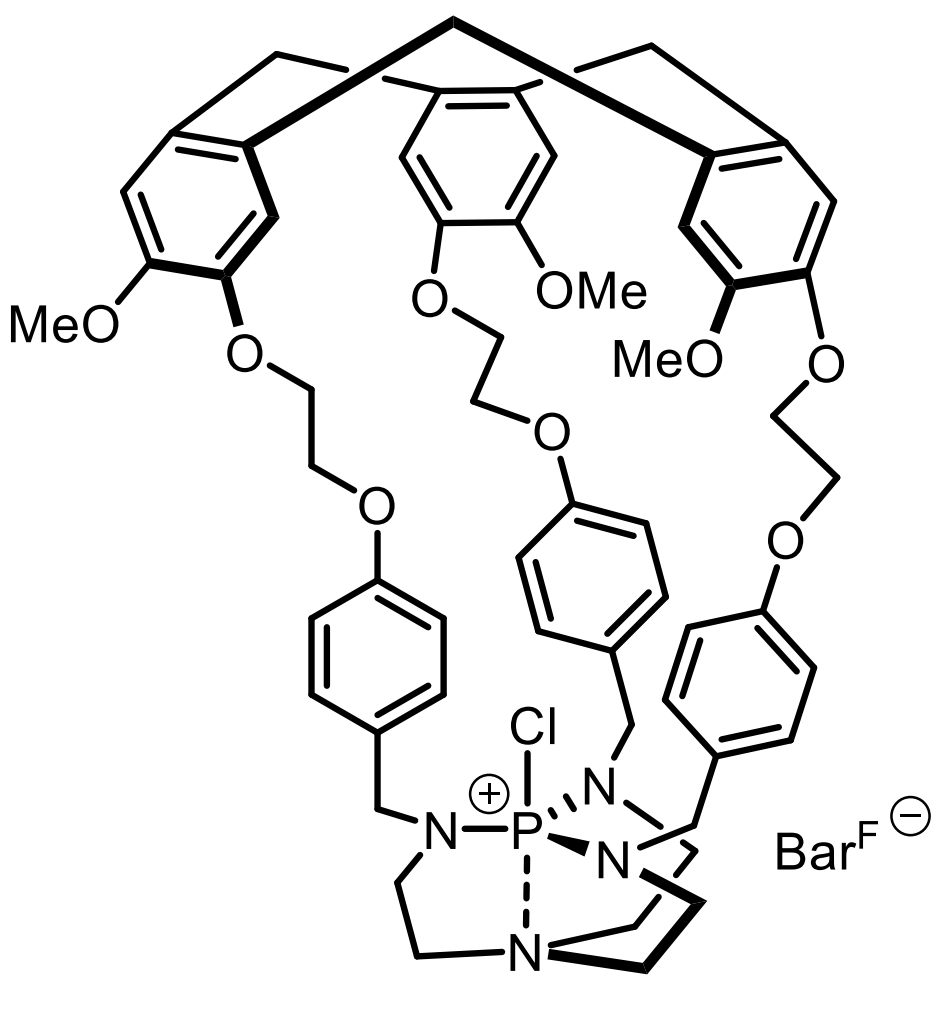

1-Cl$^+$.Bar^{F-}

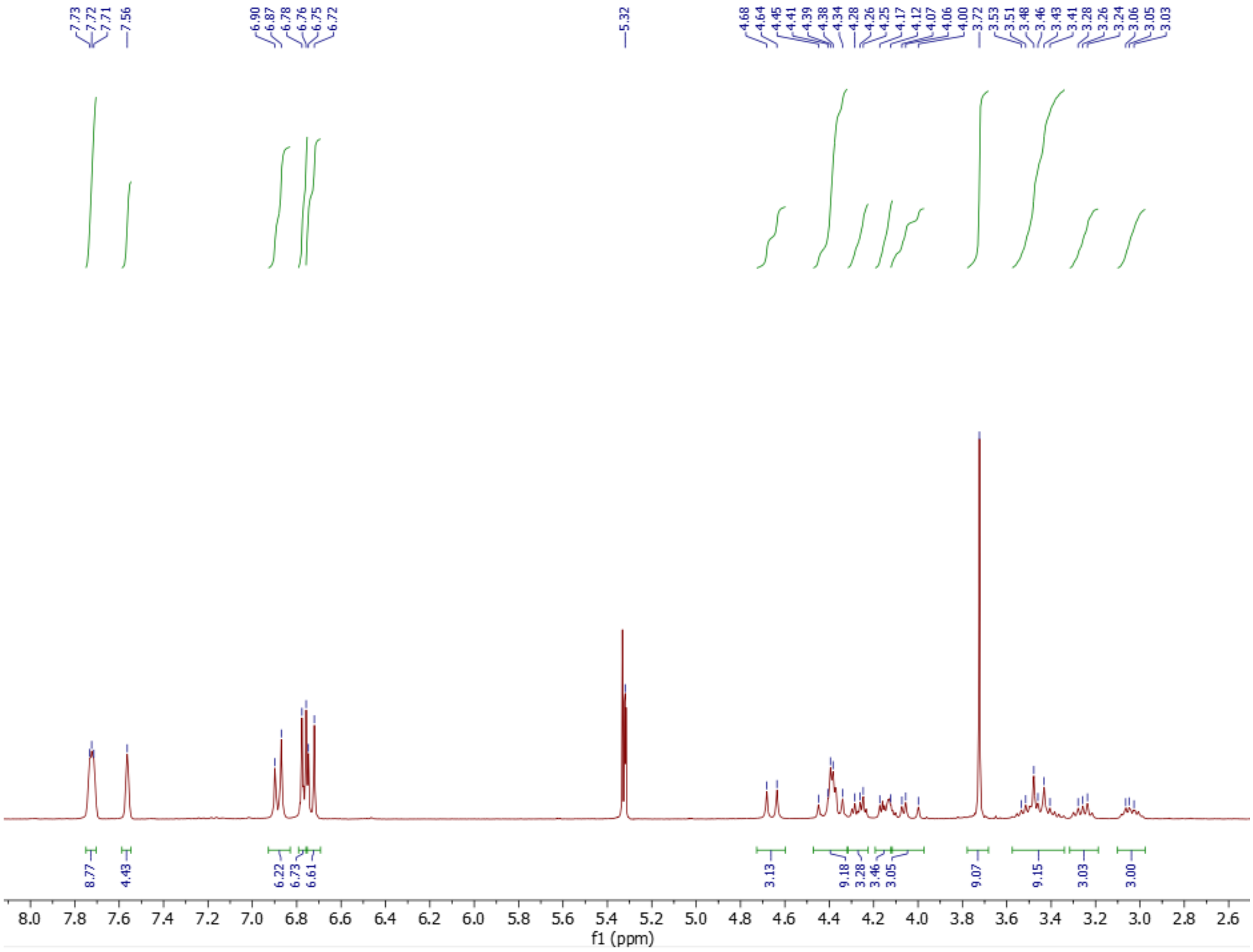

Figure S8 : ^{1}H NMR (300 MHz, CD_2Cl_2) spectrum of **1-Cl⁺.BArF⁻**

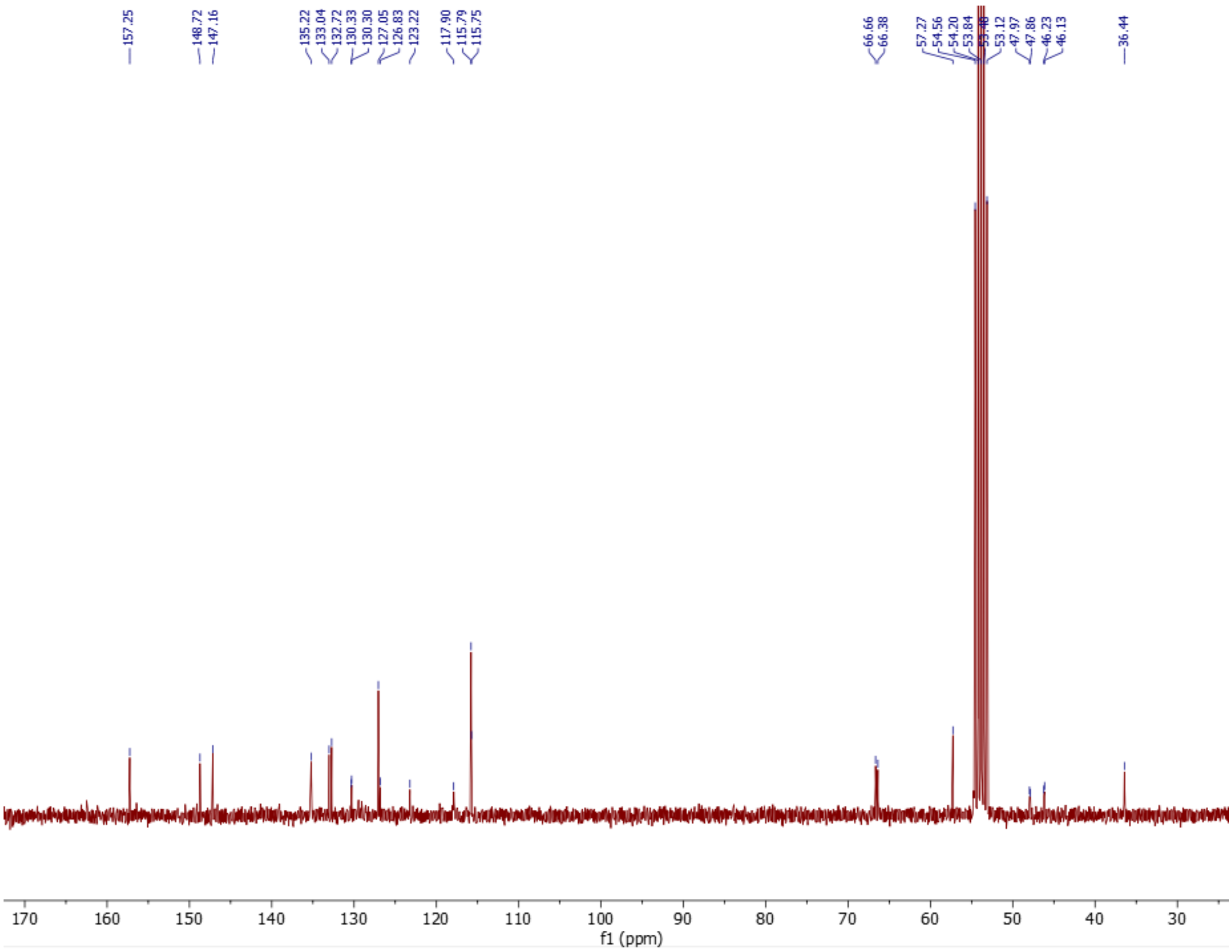

Figure S9 : ^{13}C NMR (75 MHz, CD_2Cl_2) spectrum of **1-Cl⁺.BArF⁻**

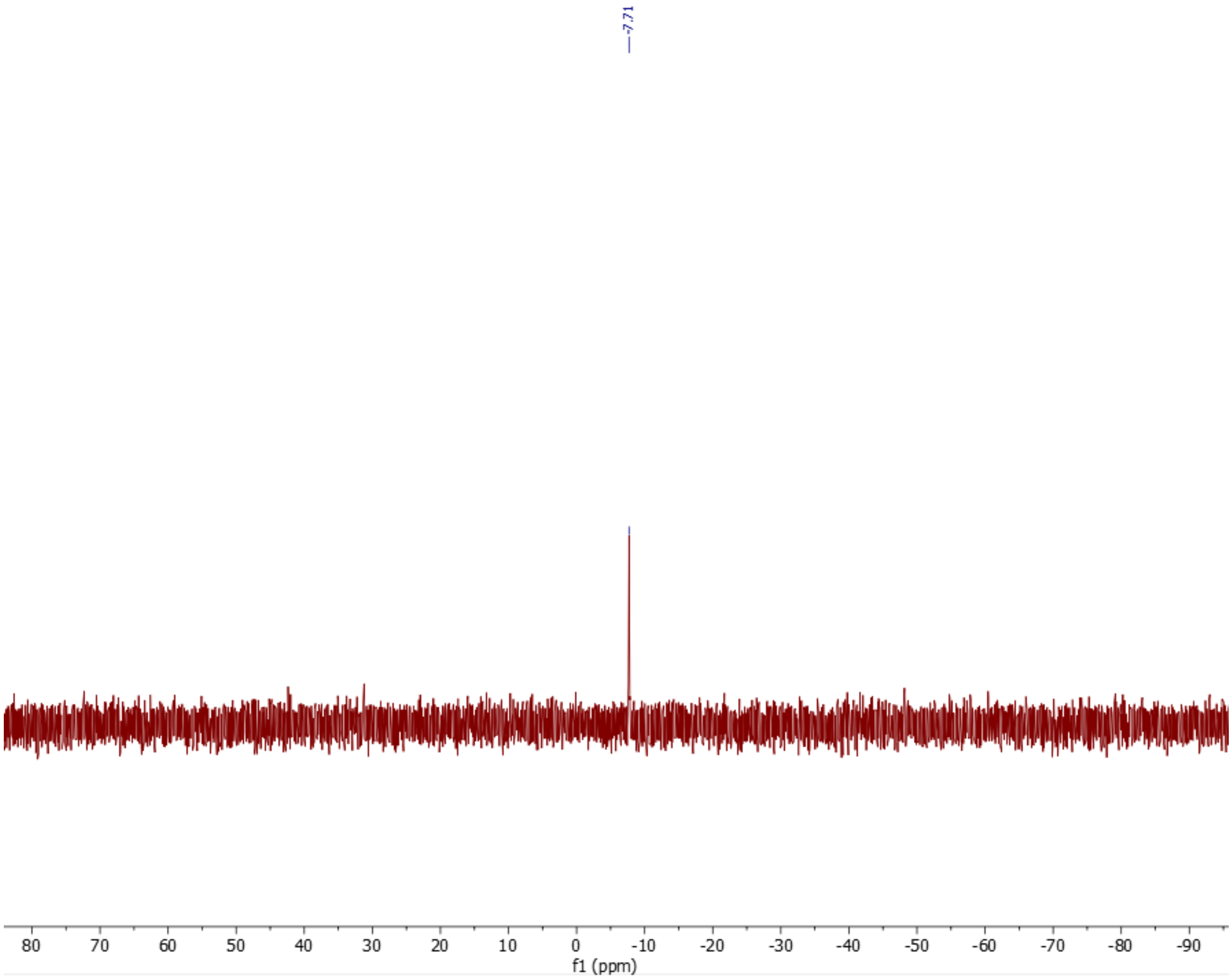

Figure S10 : ^{31}P CPD NMR (121 MHz, CD_2Cl_2) spectrum of **1-Cl$^+$.BArF$^-$**

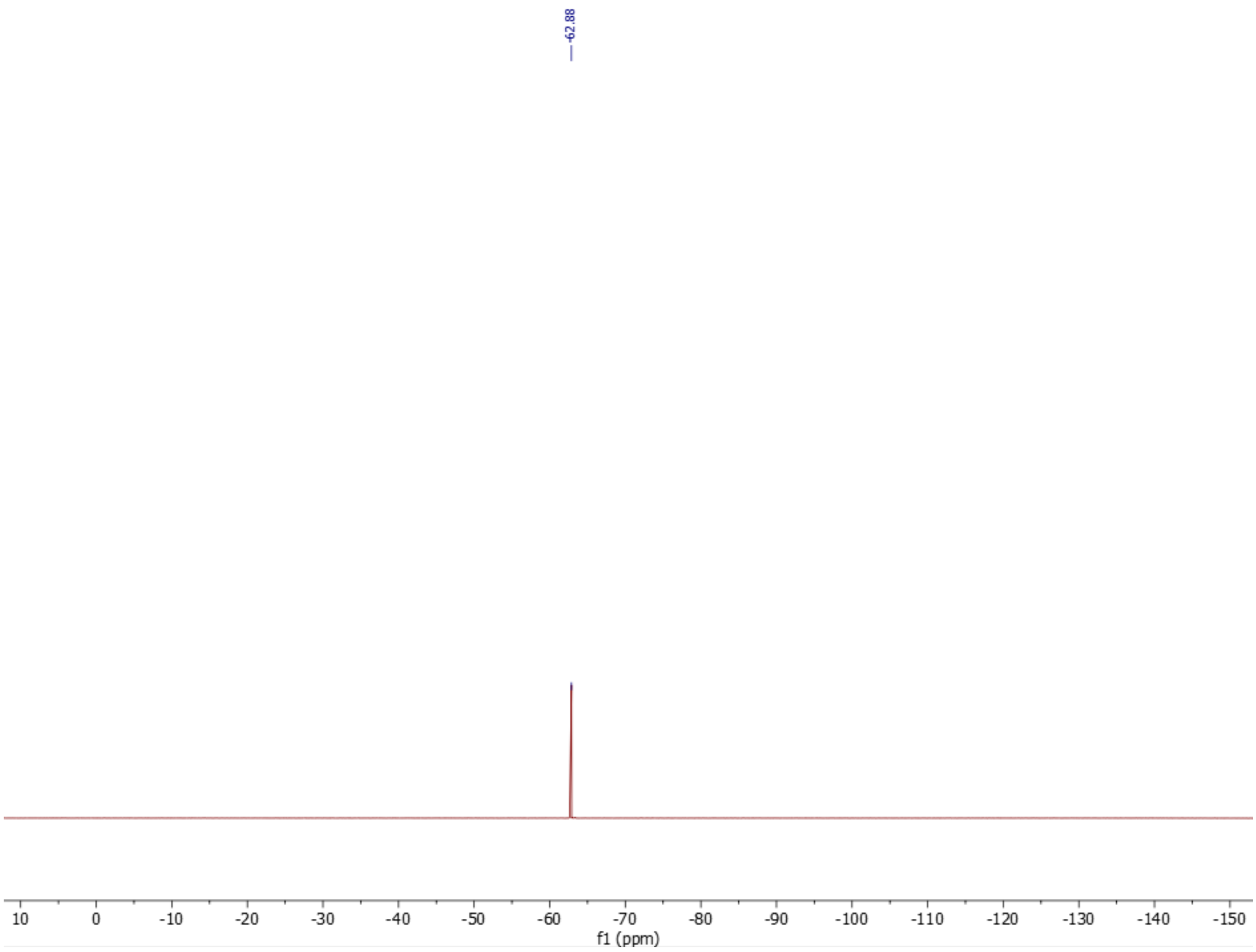

Figure S11 : ^{19}F NMR (376 MHz, CD_2Cl_2) spectrum of **1-Cl$^+$.BArF$^-$**

IV/ Mass spectra

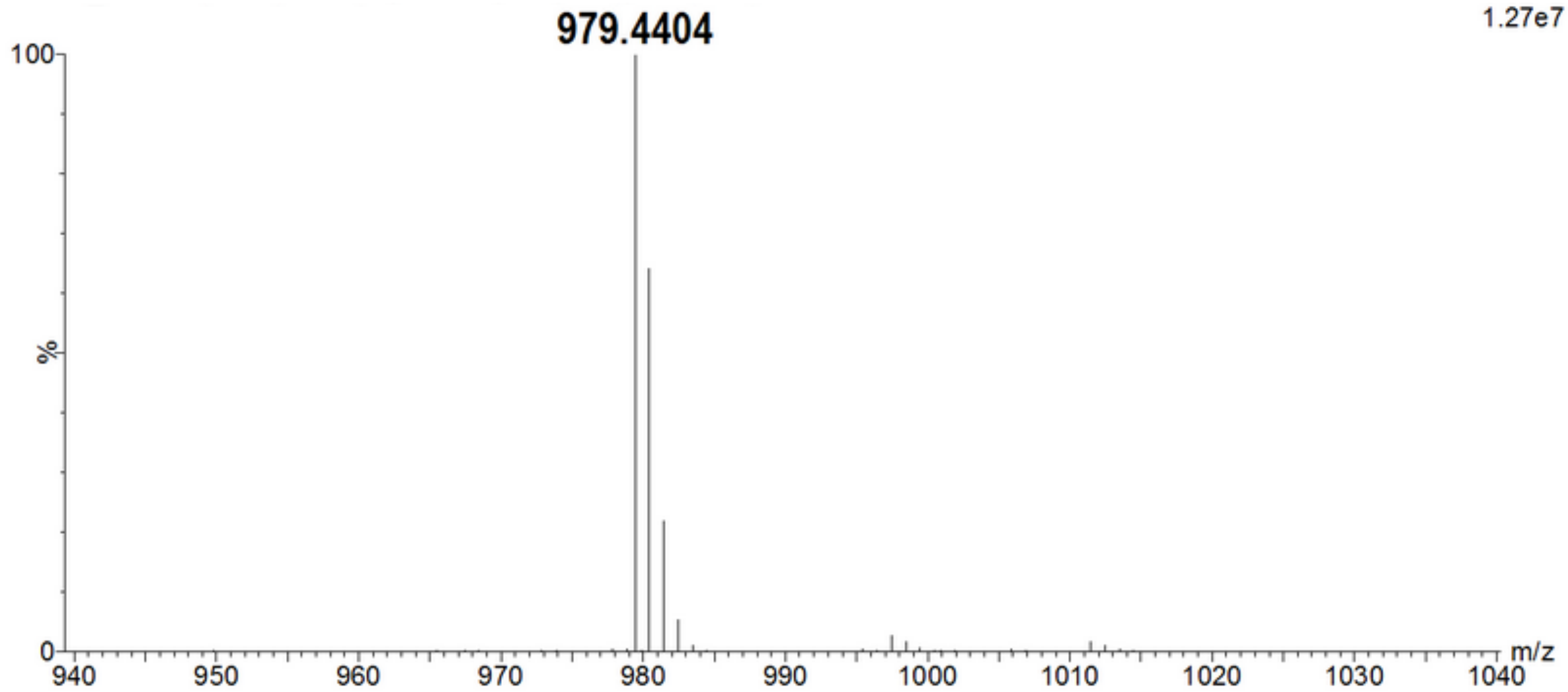

Figure S12 : Positive ESI-HRMS spectrum of **1-H⁺.Cl⁻**: *m/z* $[M]^+$ calcd for $C_{57}H_{64}N_4O_9P^+$, 979.4405; found at 979.4404.

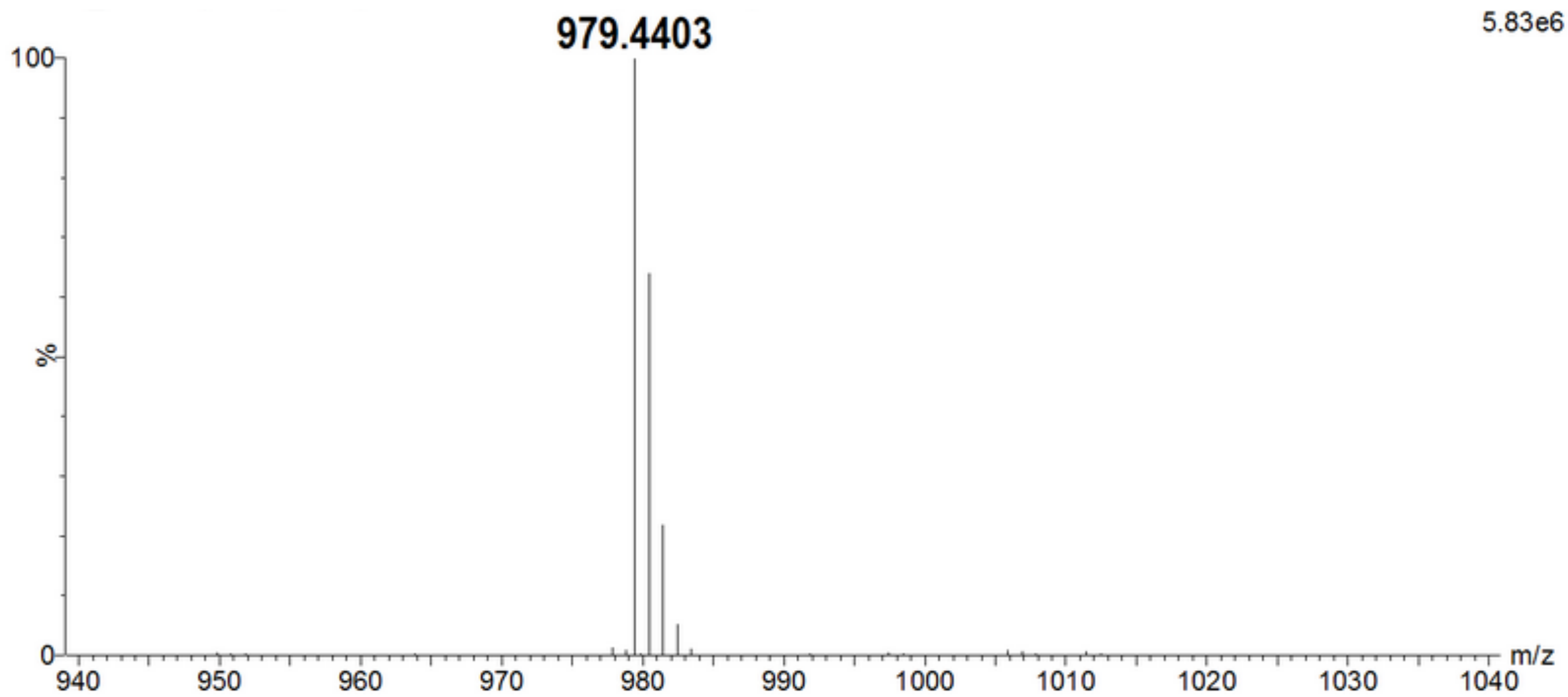

Figure S13 : Positive ESI-HRMS spectrum of **1-H⁺.BarF⁻**: *m/z* $[M]^+$ calcd for $C_{57}H_{64}N_4O_9P^+$, 979.4405; found at 979.4403.

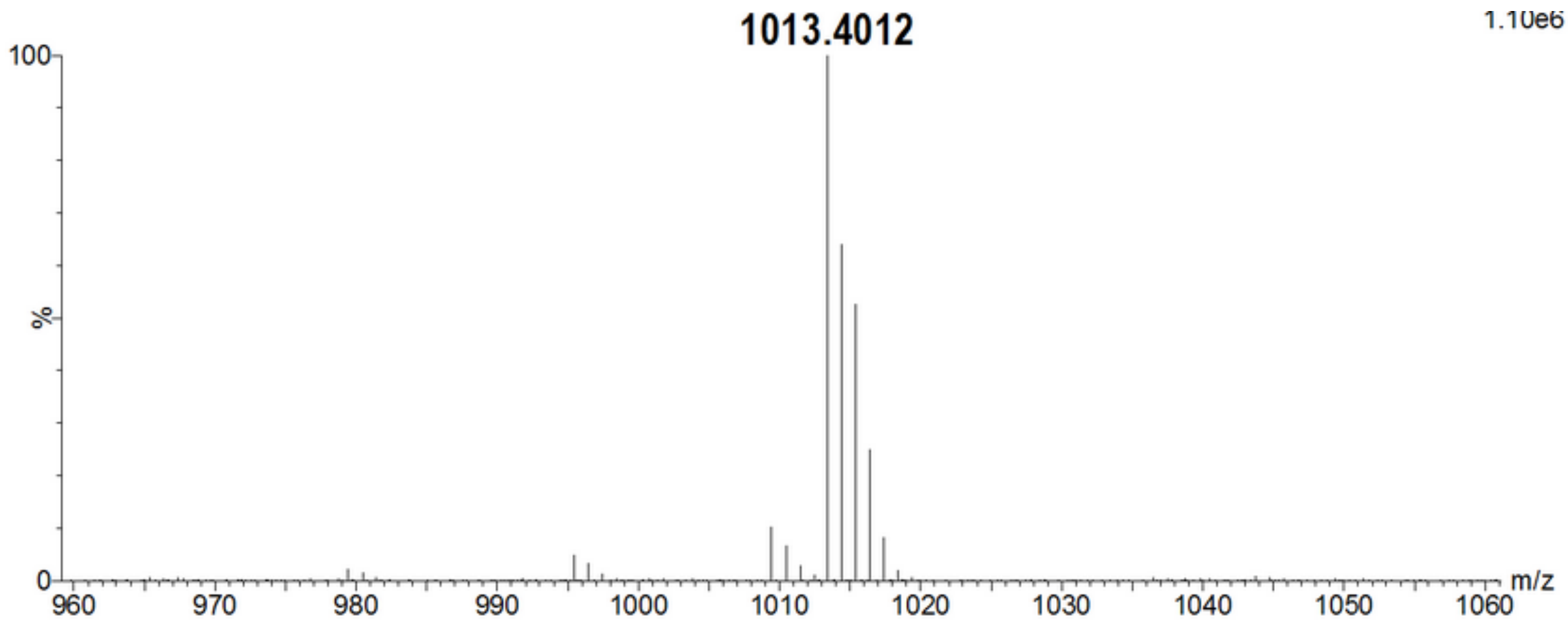

Figure S14 : Positive ESI-HRMS spectrum of **1-Cl⁺.Cl⁻**: *m/z* $[M]^+$ calcd for $C_{57}H_{63}ClN_4O_9P^+$, 1013.4016; found at 1013.4012.

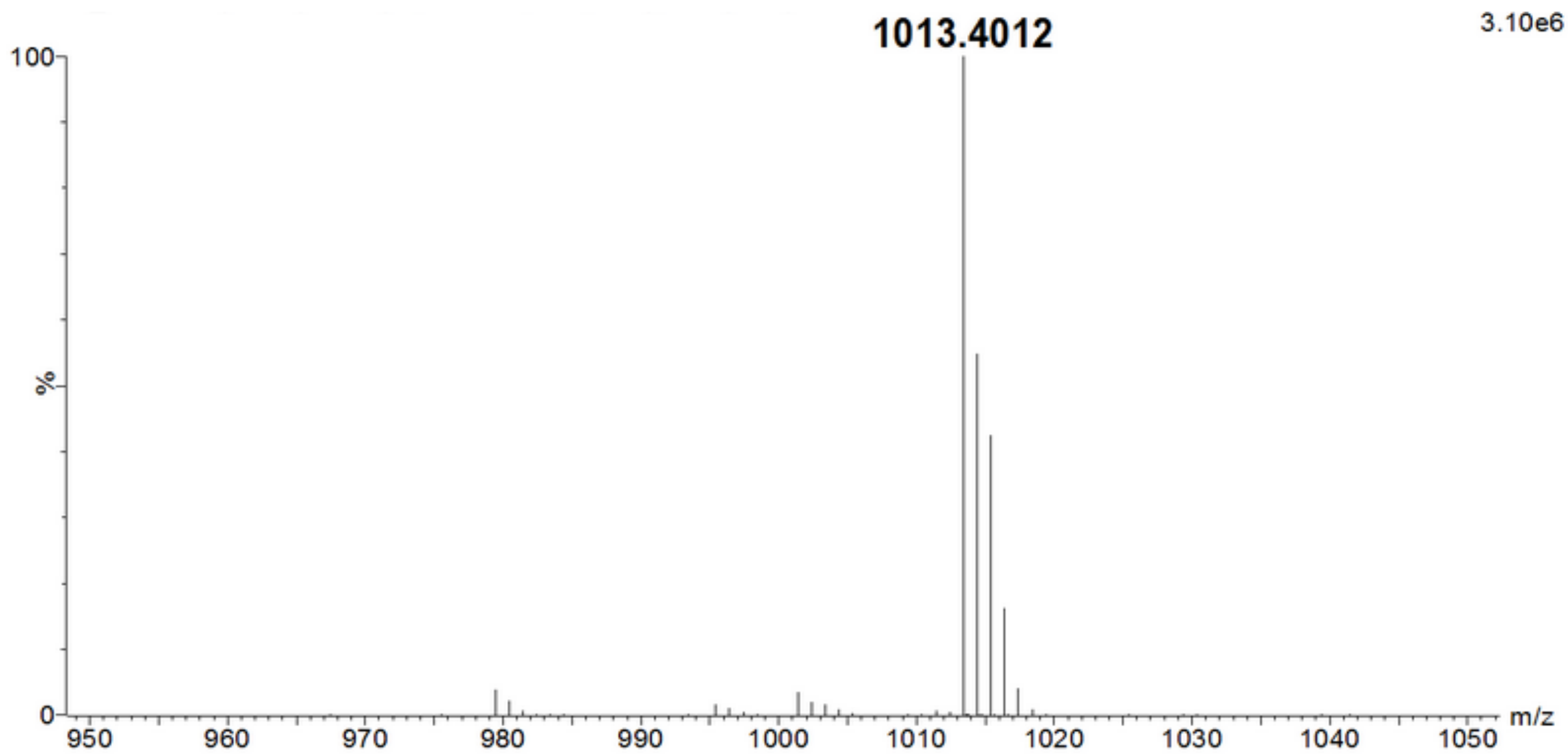

Figure S15 : Positive ESI-HRMS spectrum of **1-Cl⁺.BArF⁻**: m/z $[M]^+$ calcd for $C_{57}H_{63}ClN_4O_9P^+$, 1013.4016; found at 1013.4012.

V/ Crystallographic data

CCDC number : 2559741

The product **1-Cl⁺.Cl⁻** was crystallized from slow evaporation in chloroform.

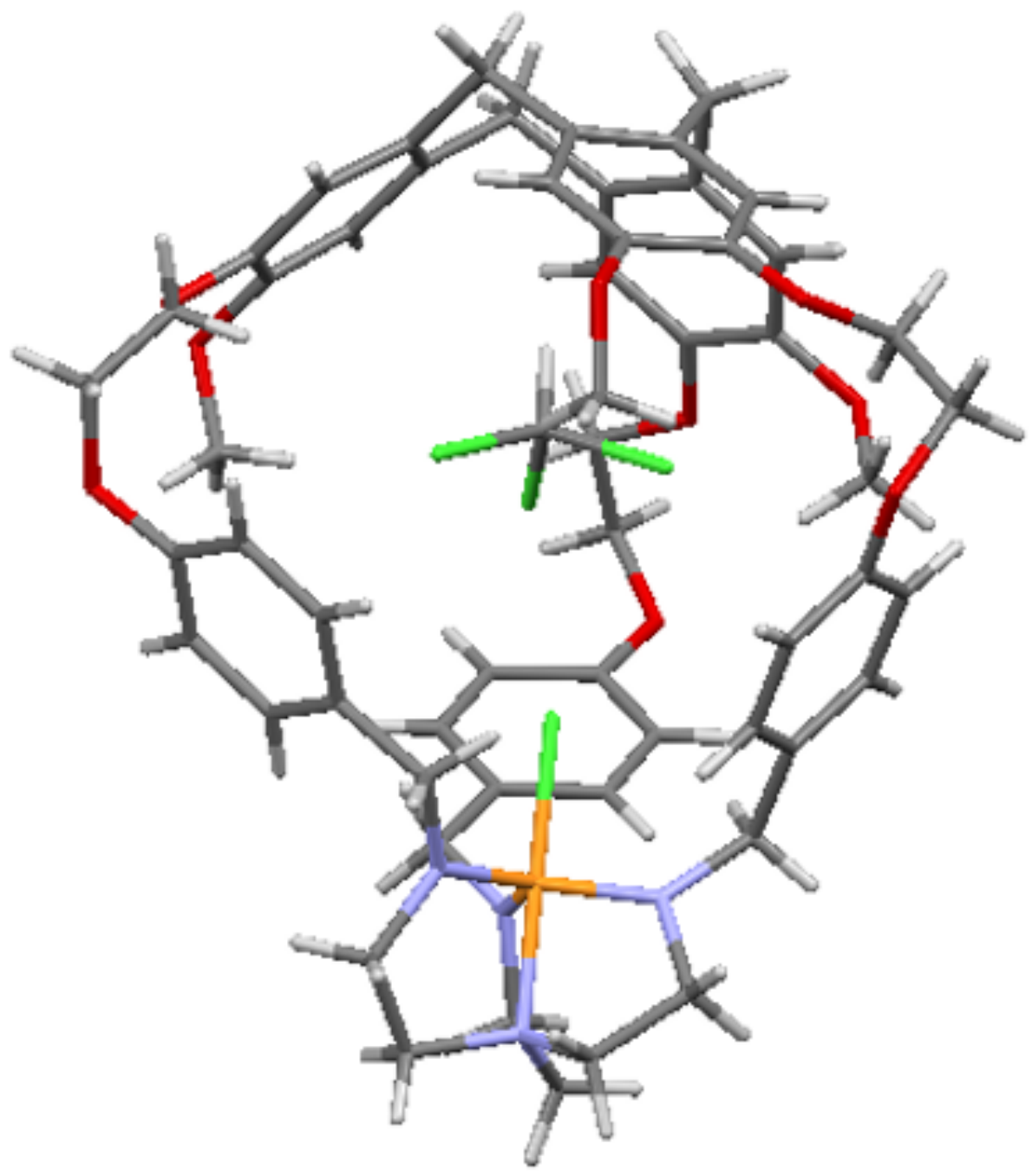

Figure S16 : Helical arrangement of **1-Cl$^+$.Cl$^-$**

**Compound**	**1-Cl$^+$.Cl$^-$**
CCDC number	2559741
Formula	$C_{61}H_{69}Cl_{14}N_4O_{10}P$
M_w	1545.47
Crystal system	Orthorhombic
Temp./ K	293
Space group	$P2_12_12_1$
a/ Å	12.2892(3)
b/ Å	24.2061(5)
c/ Å	24.4316(4)
α /°	90
β /°	90
γ /°	90
V/ Å^3	7267.76
Z	4
Dc/g.cm^{-3}	1.412
h, k, l, max	15, 29, 29
μ(Cu-Kα)/mm^{-1}	1.54184
N° of refl. measured	14016
N° of independent refl.	7690

Data	13623
parameters	832
R_1 [11348 refl.]	0.0706
wR_2 [13623 refl.]	0.2186
Goodness of fit on F^2	1.065
F(000)	3184.0

VI/ ^{1}H NMR titrations

1. General procedure

A solution of host **1-Cl$^+$.BarF$^-$** or **2-Cl$^+$.BarF$^-$** or **1-H$^+$.BarF$^-$** (5.0 mM in CD_2Cl_2, 500 µL) was titrated in NMR tubes with aliquots of a concentrated solution (50 mM in CD_2Cl_2) of guest. The chemical shifts of protons of the host were measured after each addition and plotted as a function of the guest/host ratio ([G]/[H]). The association constant K_a was obtained by nonlinear least-squares fitting of these plots using the BINDFIT program.

2. Binding constant's summary

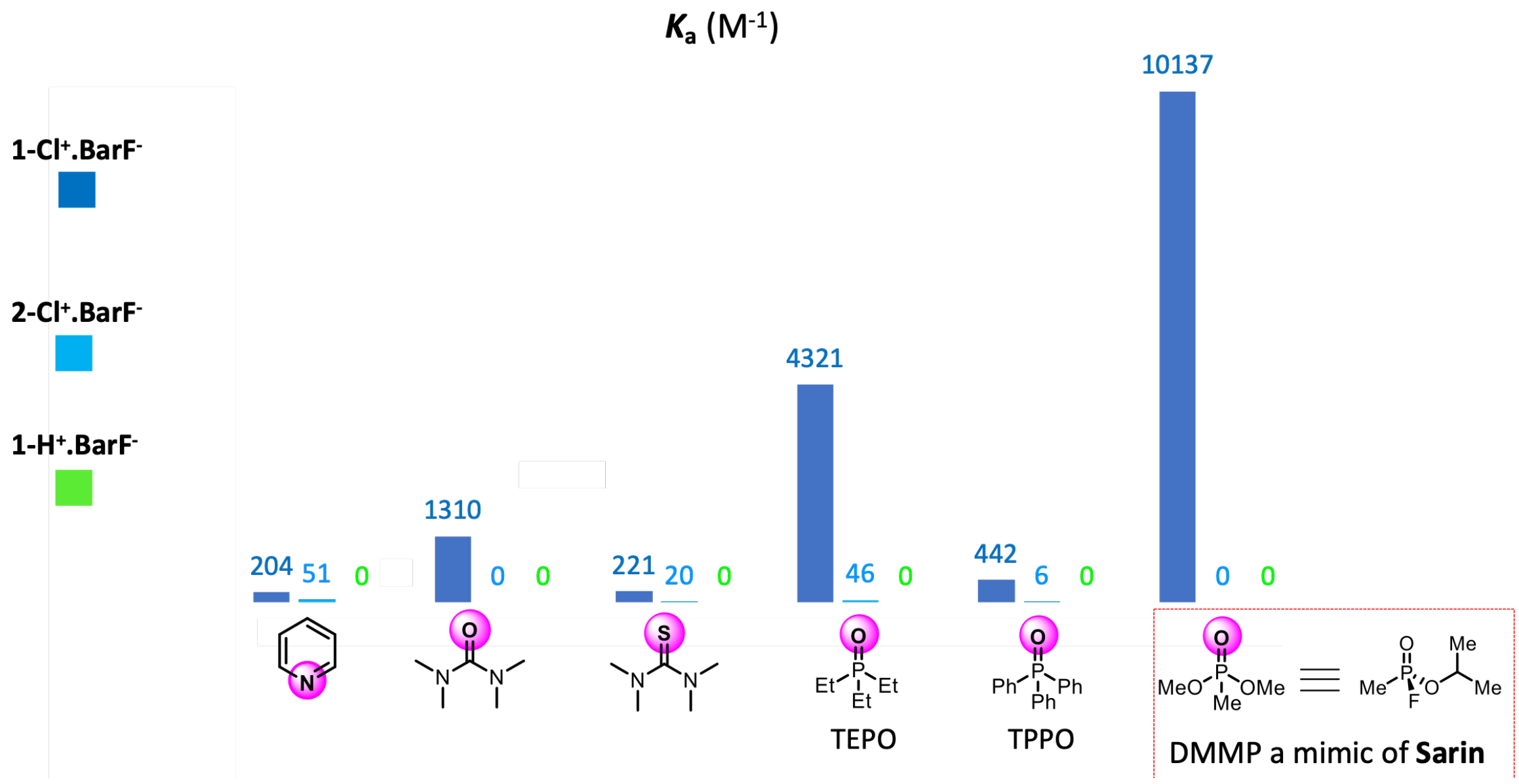

Figure S17 : Affinity constants, K_a (M^{-1}) for the association between the hosts and the guests at 300 K in CD_2Cl_2. The results reported in this graph have been obtained from the fit of the titration curves with BINDFIT program.

3. Tetramethylurea used as guest

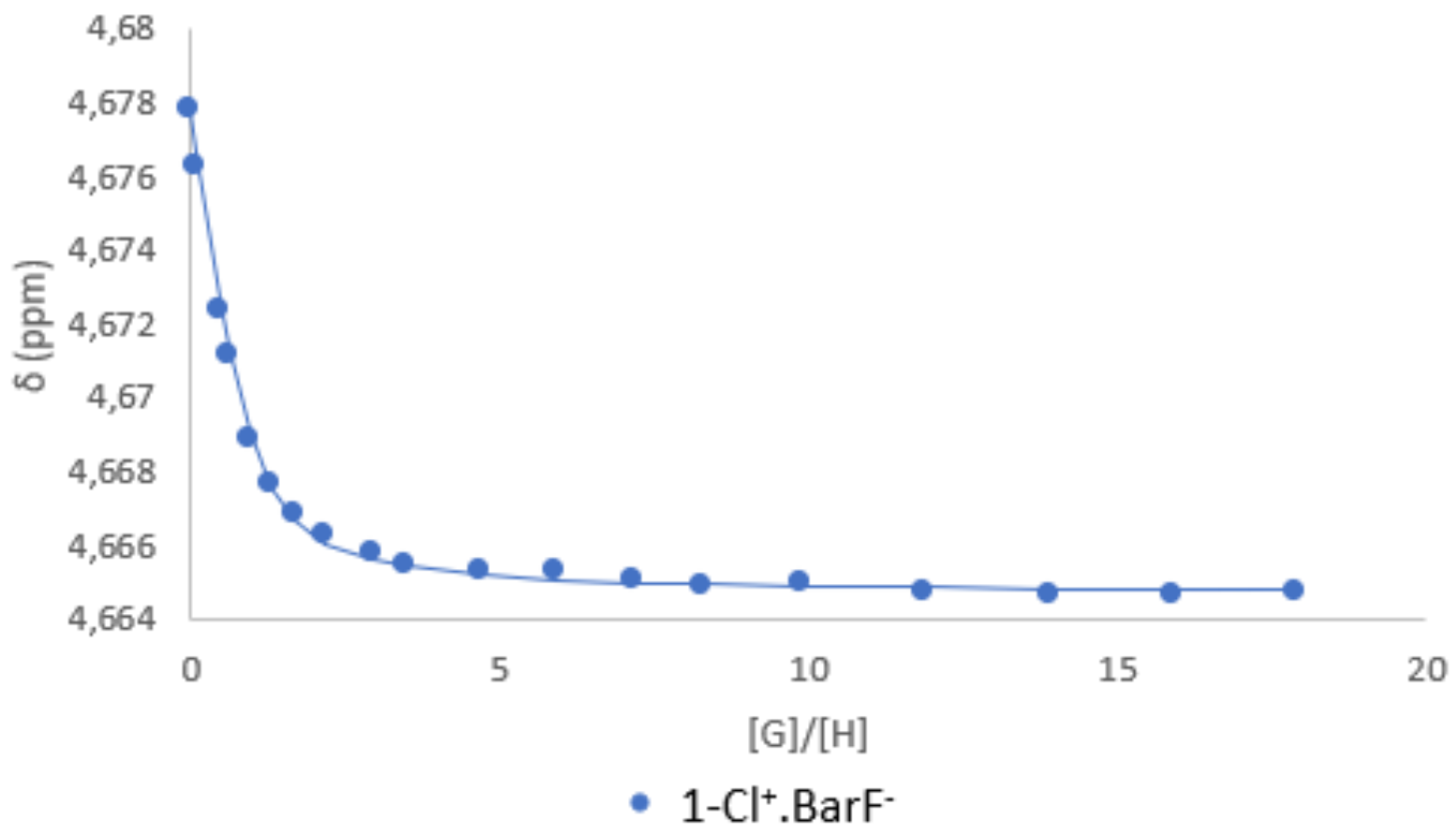

Figure S18 : Titration curve of host **1-Cl+.BarF-** (blue) with tetramethylurea. The chemical shift δ of host's protons at 4.678 ppm were measured and plotted as a function of the ratio [G]/[H] (dots). Curve was fitted with the bindfit program (line).

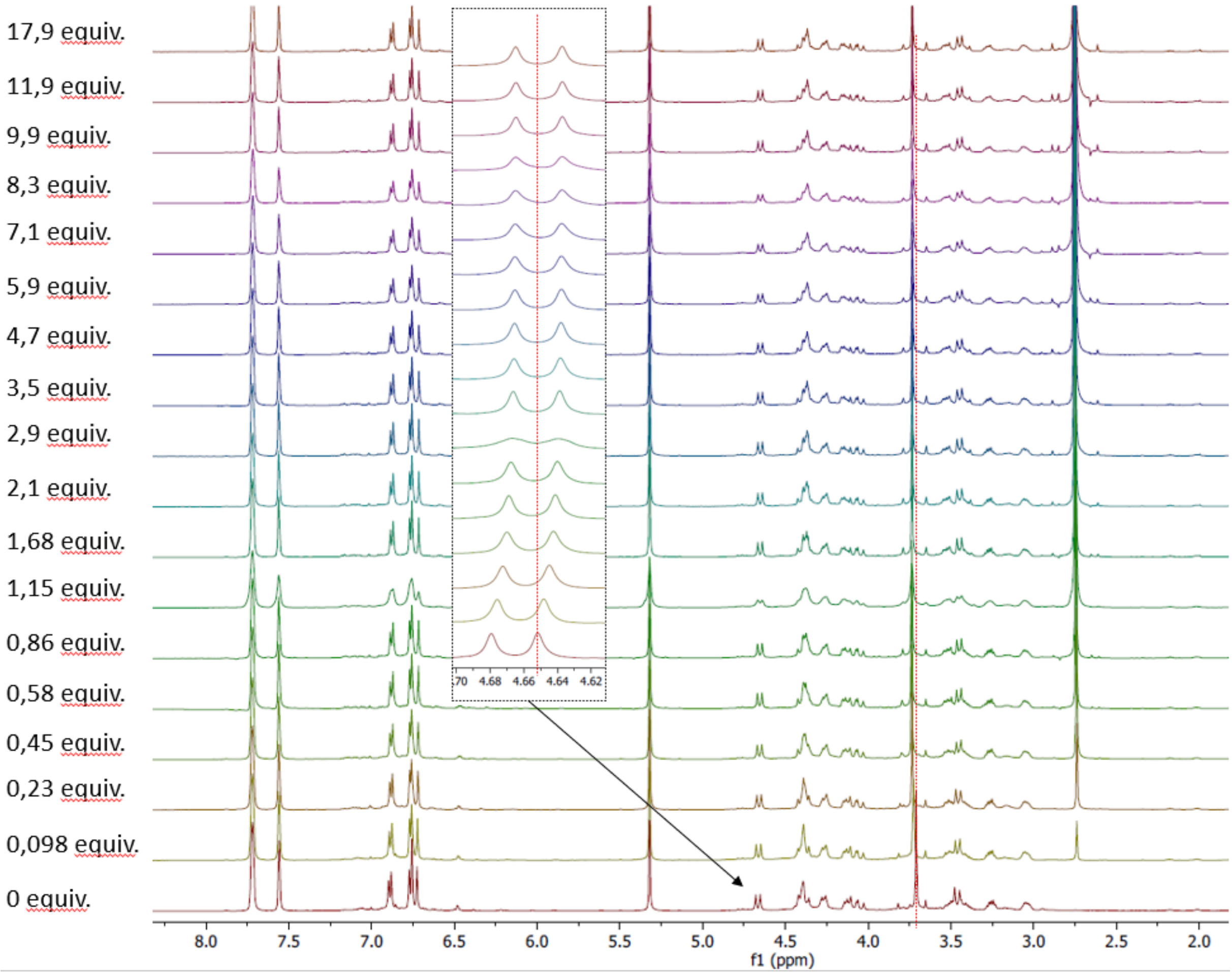

Figure S19 : ^{1}H NMR (CD_2Cl_2, 500 MHz, 300 K) spectra of [**1-Cl+.BarF-**] = 5 mM for several additions of a concentrated solution [tetramethylurea] = 50 mM.

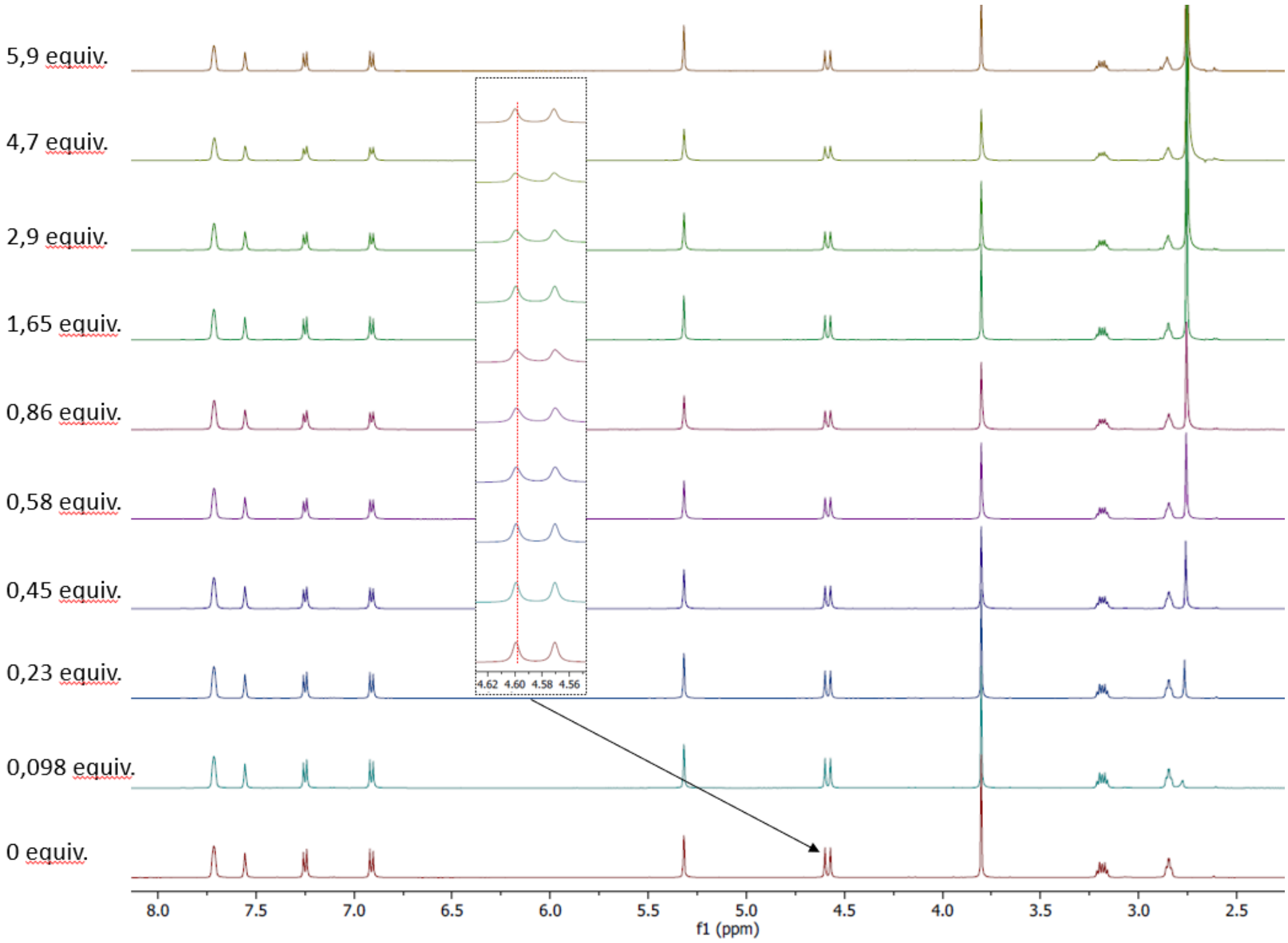

Figure S20 : ^{1}H NMR (CD_2Cl_2, 500 MHz, 300 K) spectra of [**2-Cl$^+$.BarF$^-$**] = 5 mM for several additions of a concentrated solution [tetramethylurea] = 50 mM.

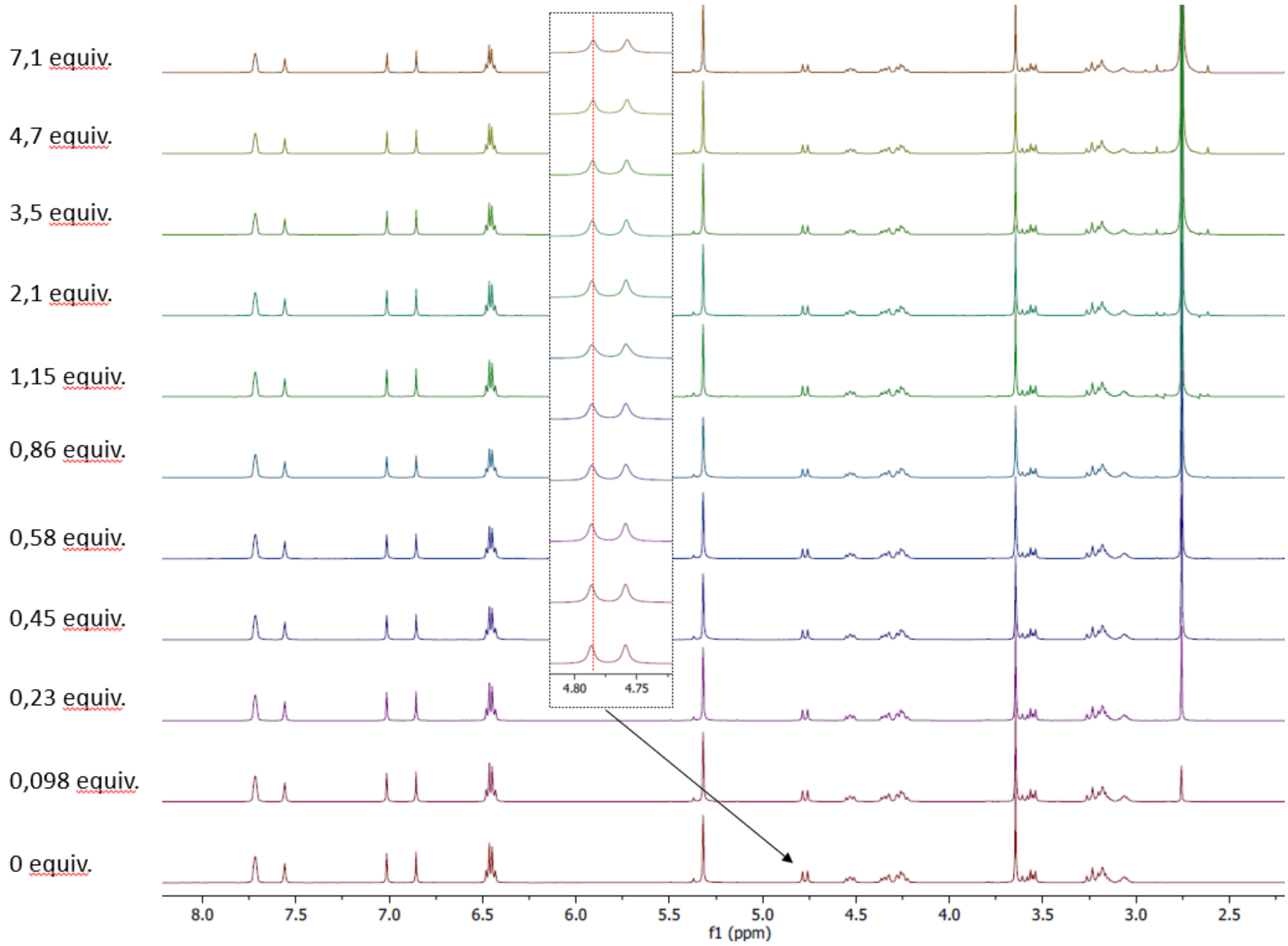

Figure S21 : ^{1}H NMR (CD_2Cl_2, 500 MHz, 300 K) spectra of [**1-H⁺.BarF⁻**] = 5 mM for several additions of a concentrated solution [tetramethylurea] = 50 mM.

4. Tetramethylthiourea used as guest

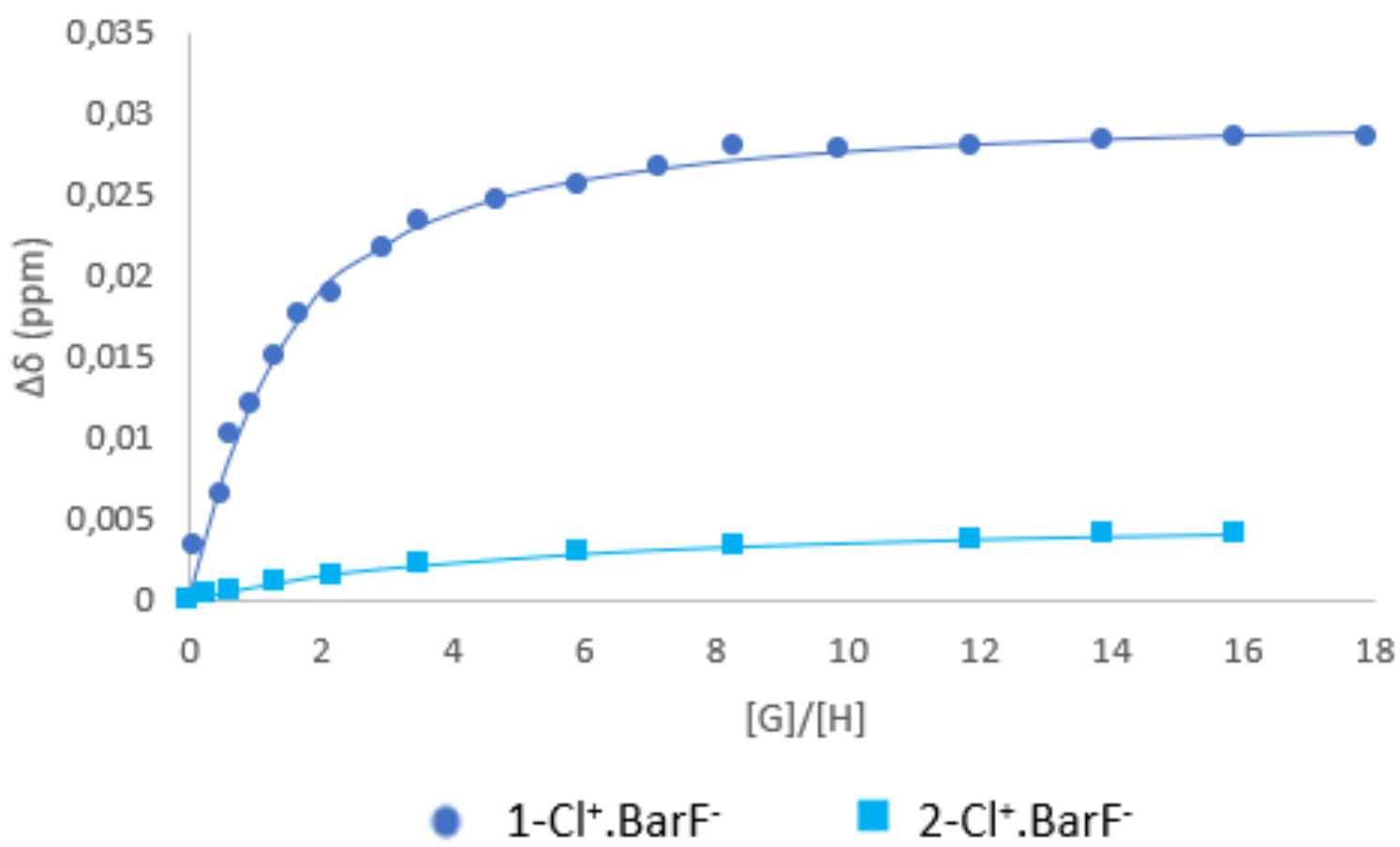

Figure S22 : Titration curve of host **1-Cl⁺.BarF⁻** (blue) and **2-Cl⁺.BarF⁻** (light blue) with tetramethyl thiourea. The chemical shift Δδ of host's protons at 3.265 ppm (**1-Cl⁺.BarF⁻**) and 3.201 ppm (**2-Cl⁺.BarF⁻**) were measured and plotted as a function of the ratio [G]/[H] (dots). Curves were fitted with the bindfit program (lines).

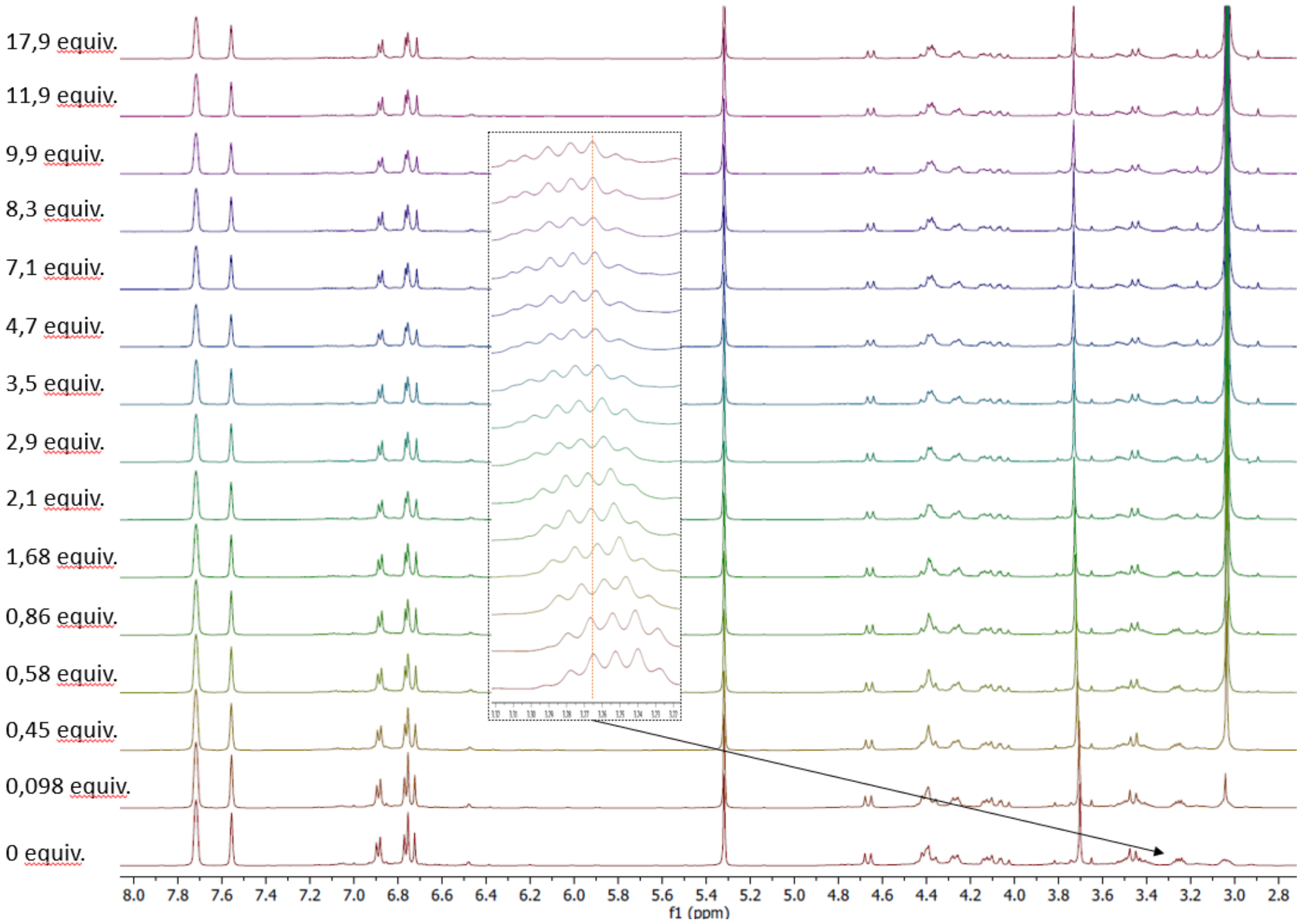

Figure S23 : ^{1}H NMR (CD_2Cl_2, 500 MHz, 300 K) spectra of [**1-Cl⁺.BarF⁻**] = 5 mM for several additions of a concentrated solution [tetramethyl thiourea] = 50 mM.

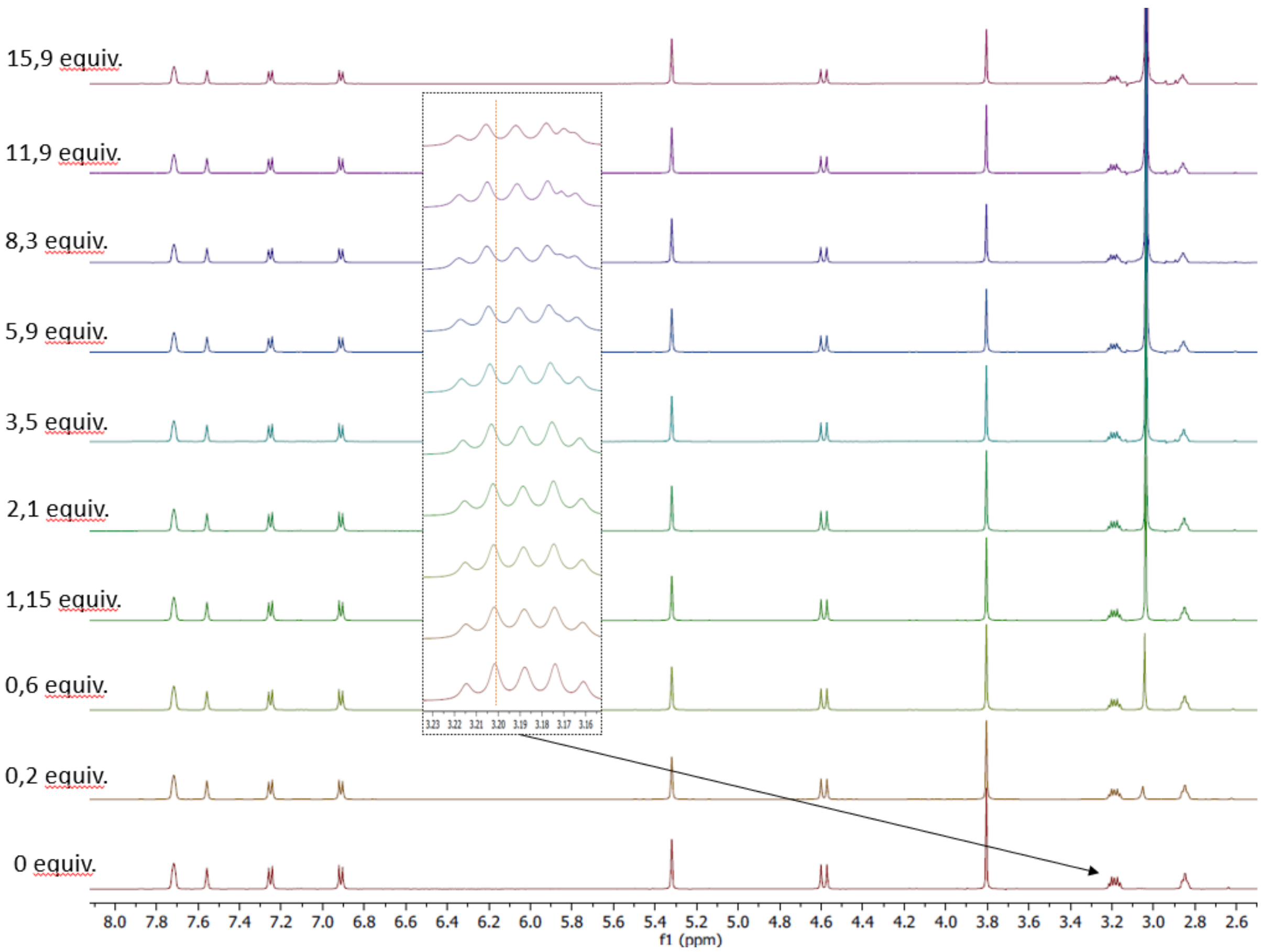

Figure S24 : ^{1}H NMR (CD_2Cl_2, 500 MHz, 300 K) spectra of [**2-Cl⁺.BarF⁻**] = 5 mM for several additions of a concentrated solution [tetramethyl thiourea] = 50 mM.

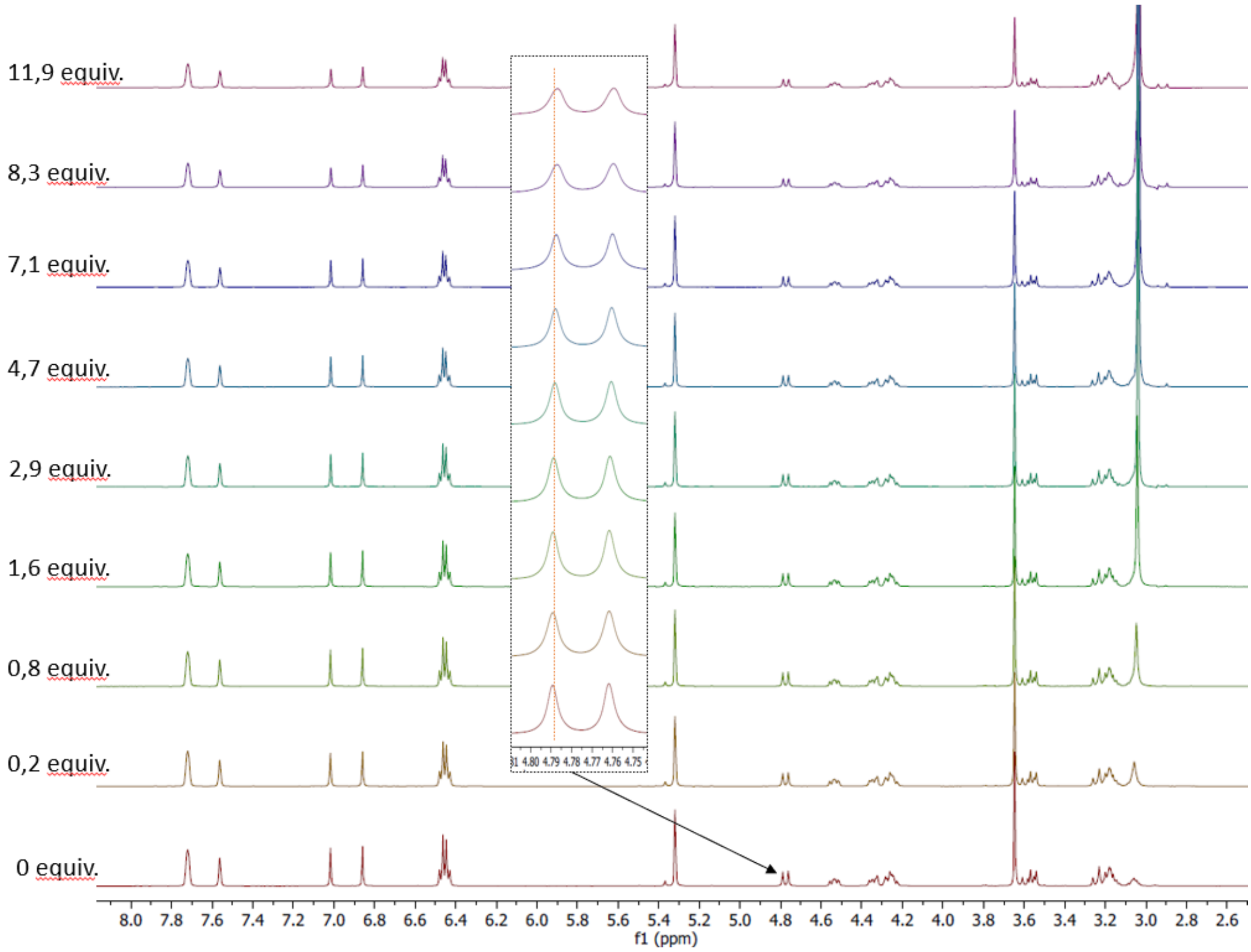

Figure S25 : ^{1}H NMR (CD_2Cl_2, 500 MHz, 300 K) spectra of [**1-H⁺.BarF⁻**] = 5 mM for several additions of a concentrated solution [tetramethyl thiourea] = 50 mM.

5. Pyridine used as guest

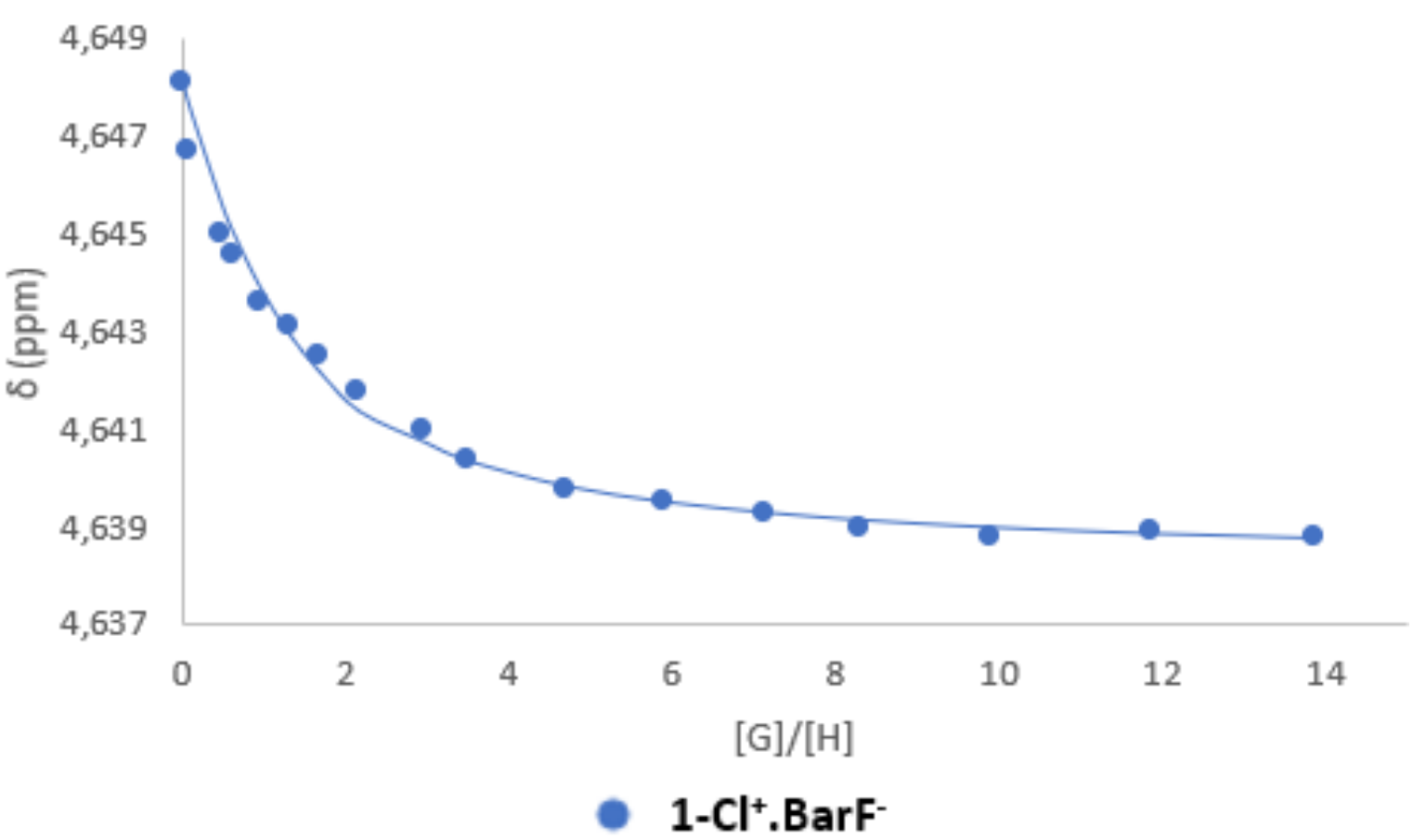

Figure S26 : Titration curve of host **1-Cl⁺.BarF⁻** with pyridine. The chemical shift changes Δδ of host's protons at 4.648 ppm were measured and plotted as a function of the ratio [G]/[H] (dots). Curve was fitted with the bindfit program (line).

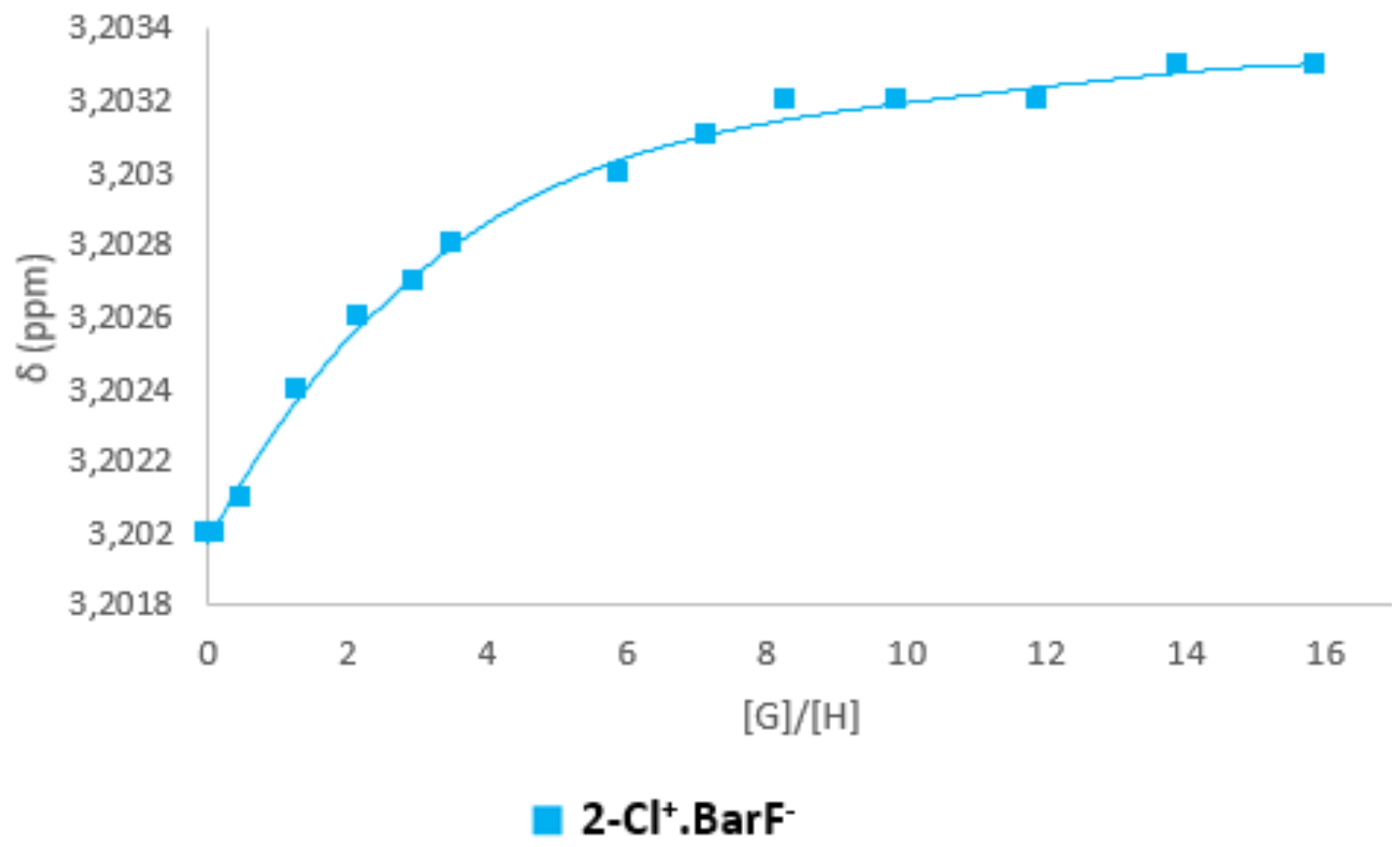

Figure S27 : Titration curve of host **2-Cl⁺.BarF⁻** with pyridine. The chemical shift changes Δδ of host's protons at 3.202 ppm were measured and plotted as a function of the ratio [G]/[H] (dots). Curve was fitted with the bindfit program (line).

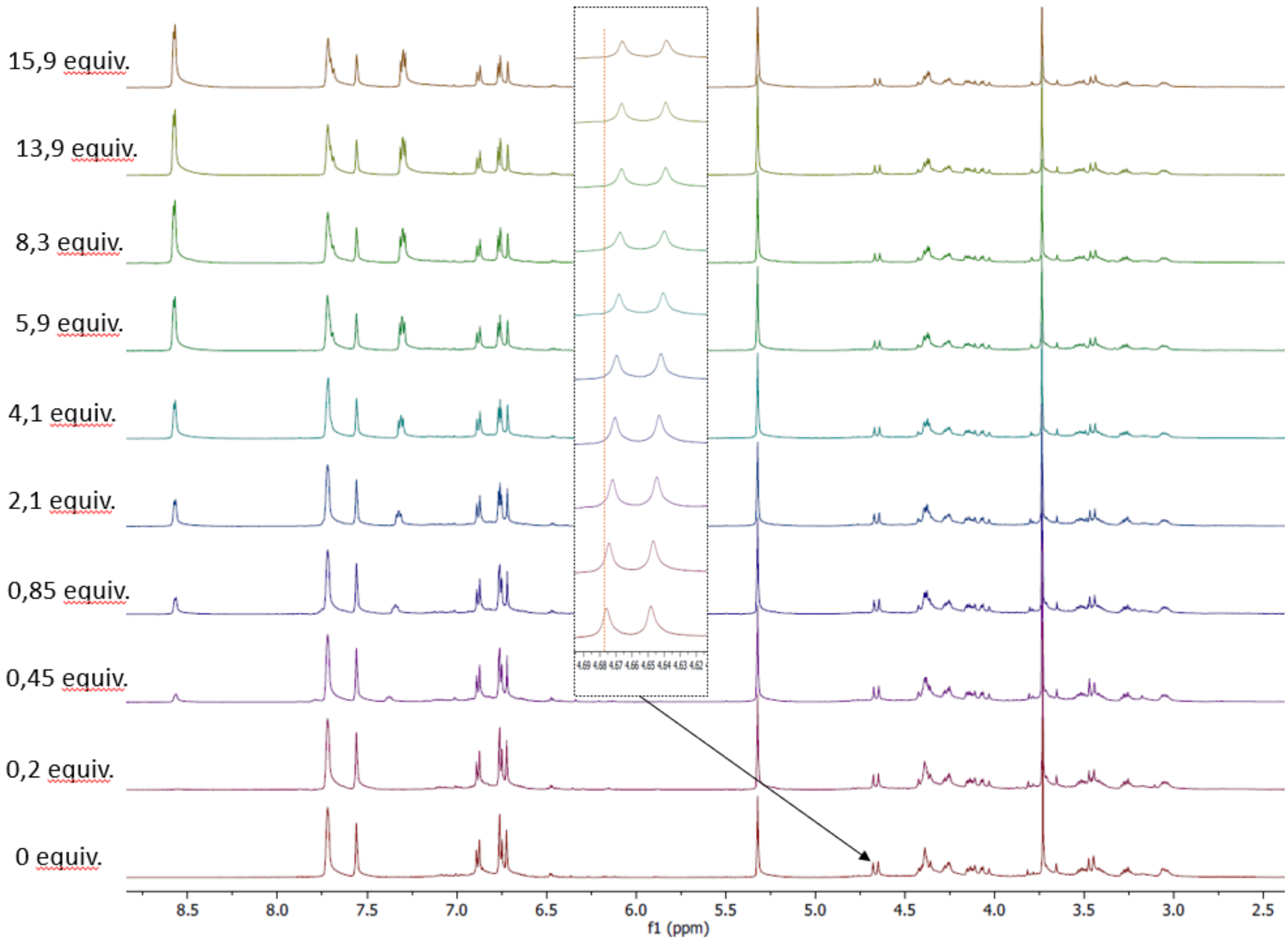

Figure S28 : ^{1}H NMR (CD_2Cl_2, 500 MHz, 300 K) spectra of [**1-Cl⁺.BarF⁻**] = 5 mM for several additions of a concentrated solution [pyridine] = 50 mM.

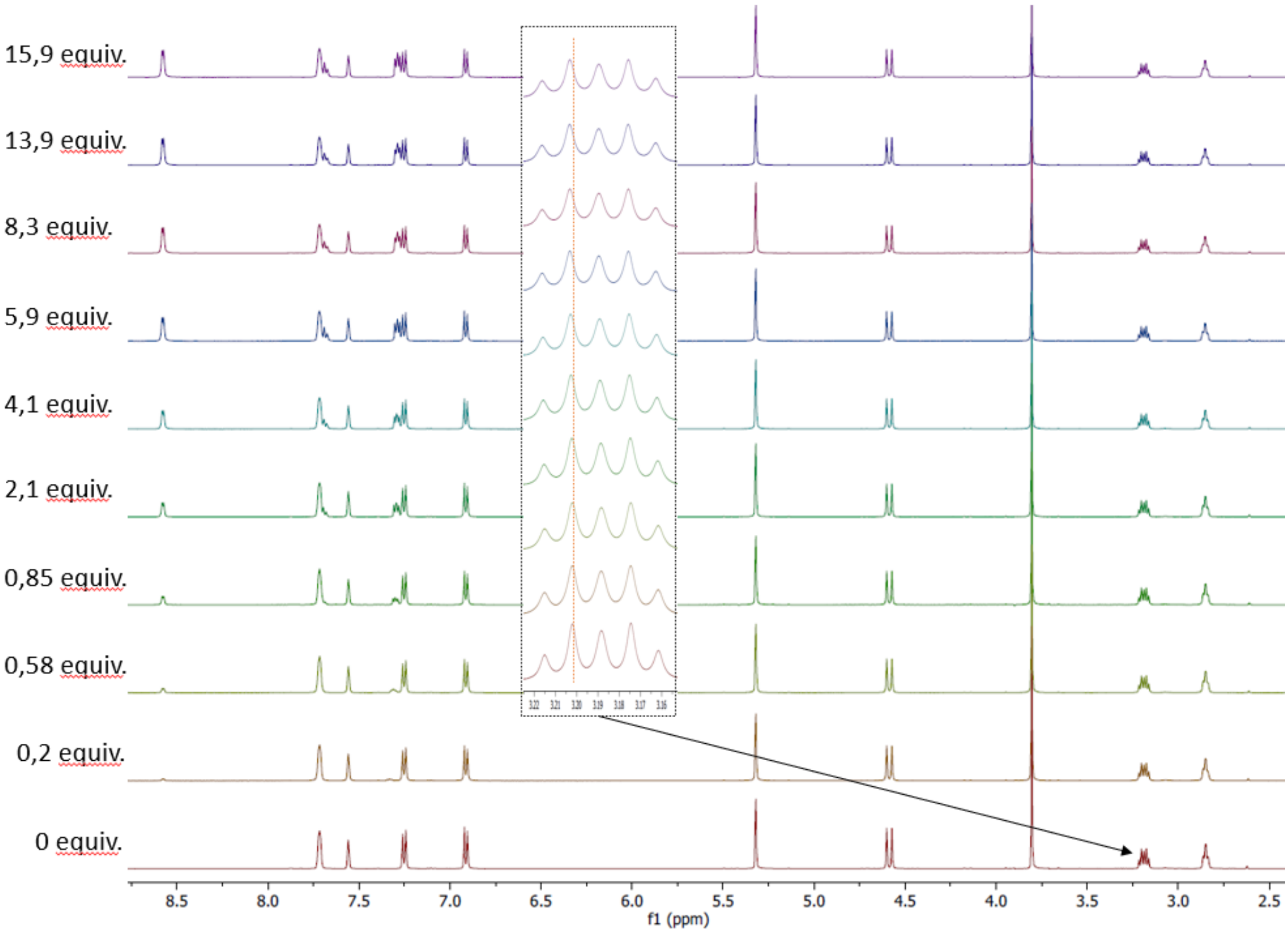

Figure S29 : ^{1}H NMR (CD_2Cl_2, 500 MHz, 300 K) spectra of [**2-Cl⁺.BarF⁻**] = 5 mM for several additions of a concentrated solution [pyridine] = 50 mM.

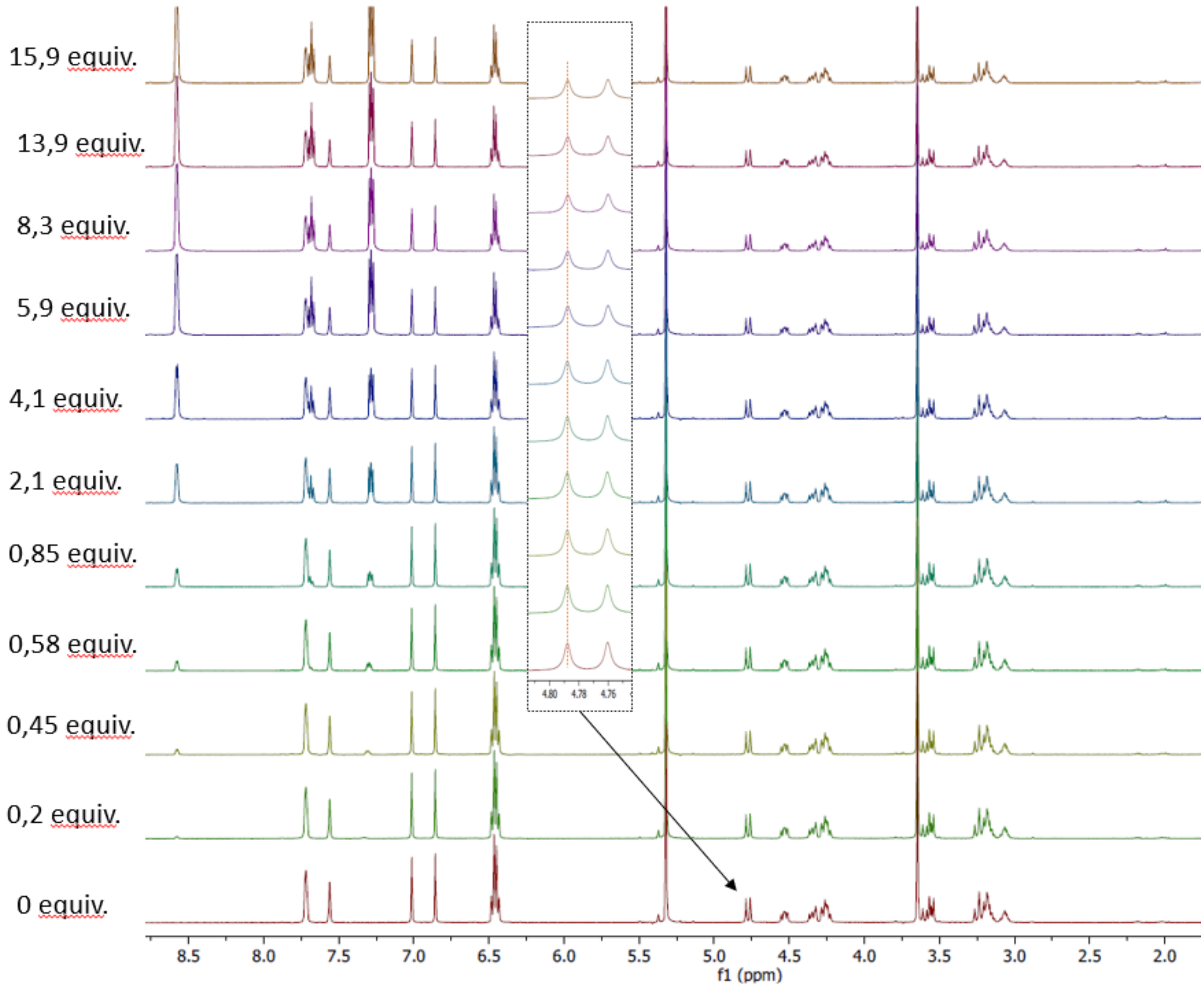

Figure S30 : ^{1}H NMR (CD_2Cl_2, 500 MHz, 300 K) spectra of [**1-H$^+$.BarF$^-$**] = 5 mM for several additions of a concentrated solution [pyridine] = 50 mM.

6. Triethylphosphine oxide (TEPO) used as guest

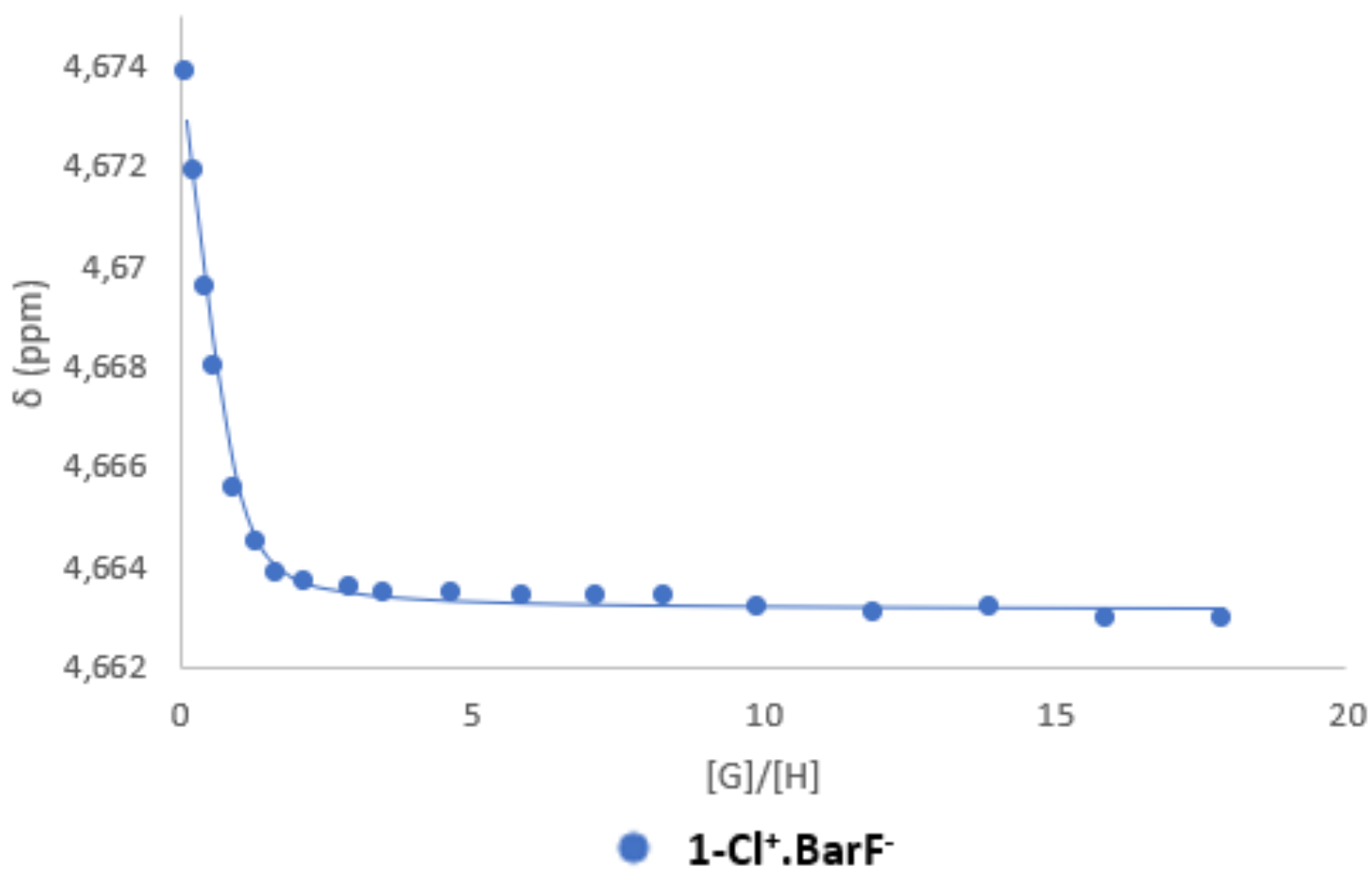

Figure S31 : Titration curve of host **1-Cl$^+$.BarF$^-$** with TEPO. The chemical shift changes Δδ of host's protons at 4.674 ppm were measured and plotted as a function of the ratio [G]/[H] (dots). Curve was fitted with the bindfit program (line).

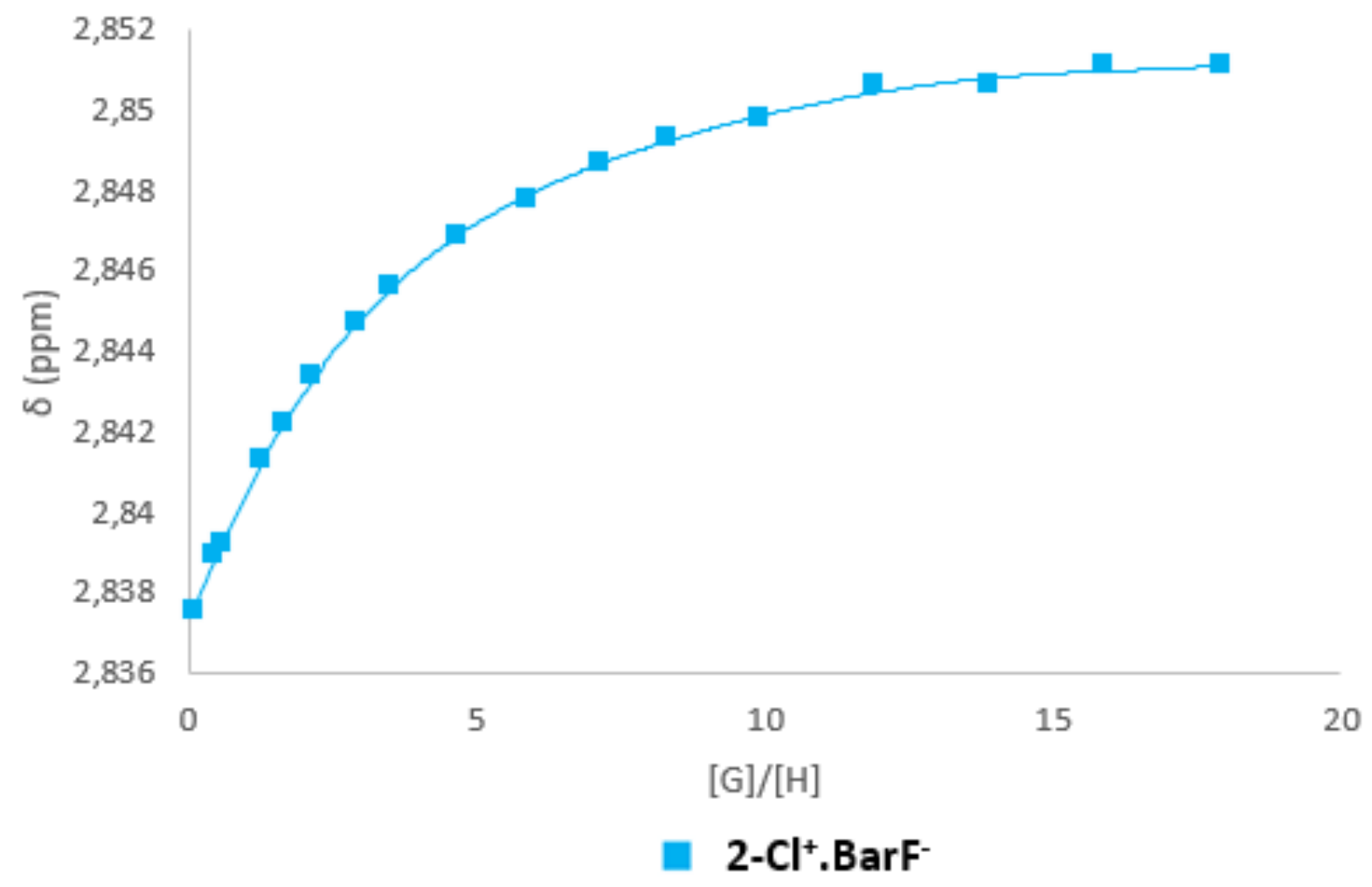

Figure S32 : Titration curve of host **2-Cl$^+$.BarF$^-$** with TEPO. The chemical shift changes Δδ of host's protons at 2.838 ppm were measured and plotted as a function of the ratio [G]/[H] (dots). Curve was fitted with the bindfit program (line).

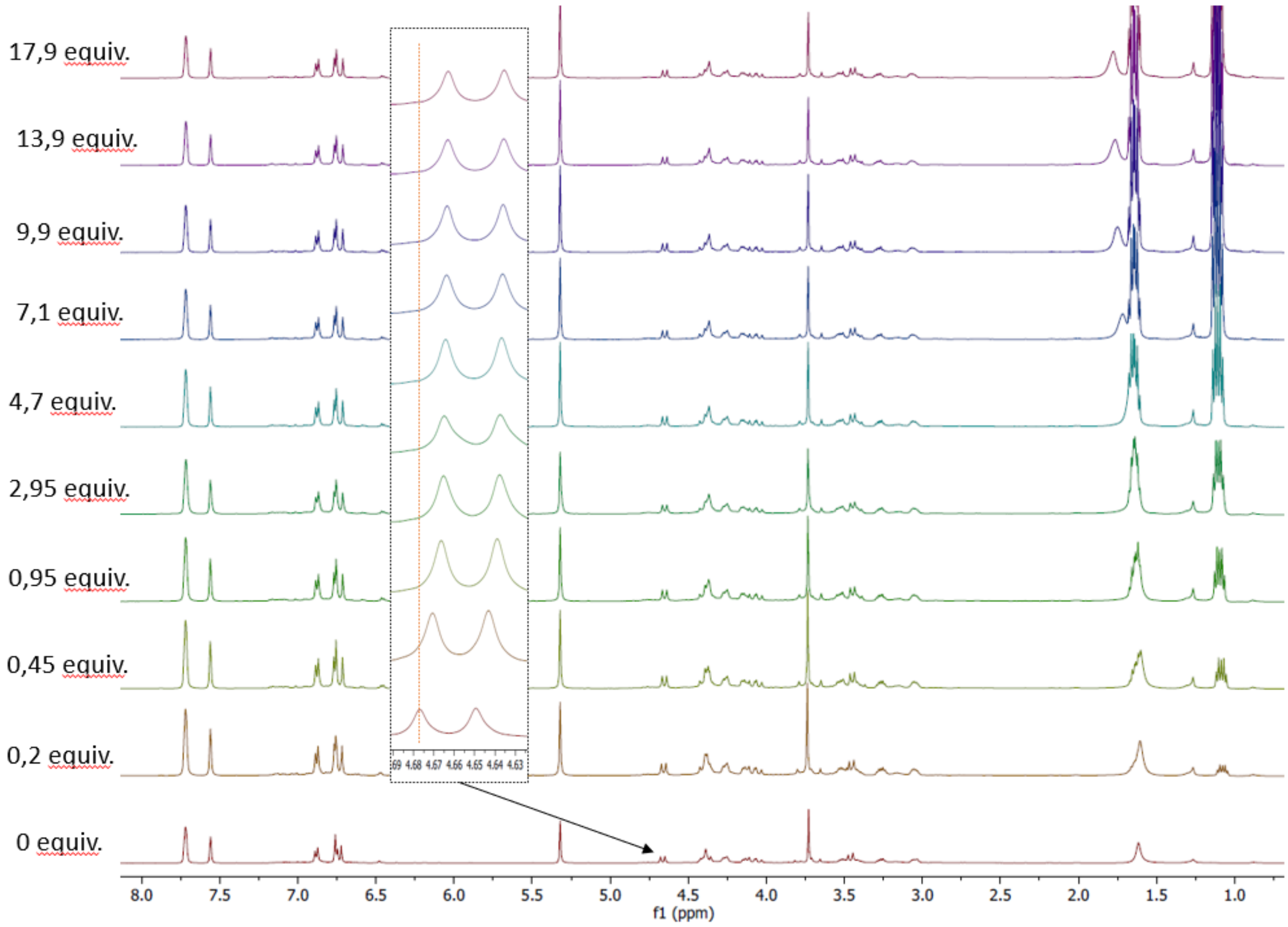

Figure S33 : ^{1}H NMR (CD_2Cl_2, 500 MHz, 300 K) spectra of [**1-Cl$^+$.BarF$^-$**] = 5 mM for several additions of a concentrated solution [TEPO] = 50 mM.

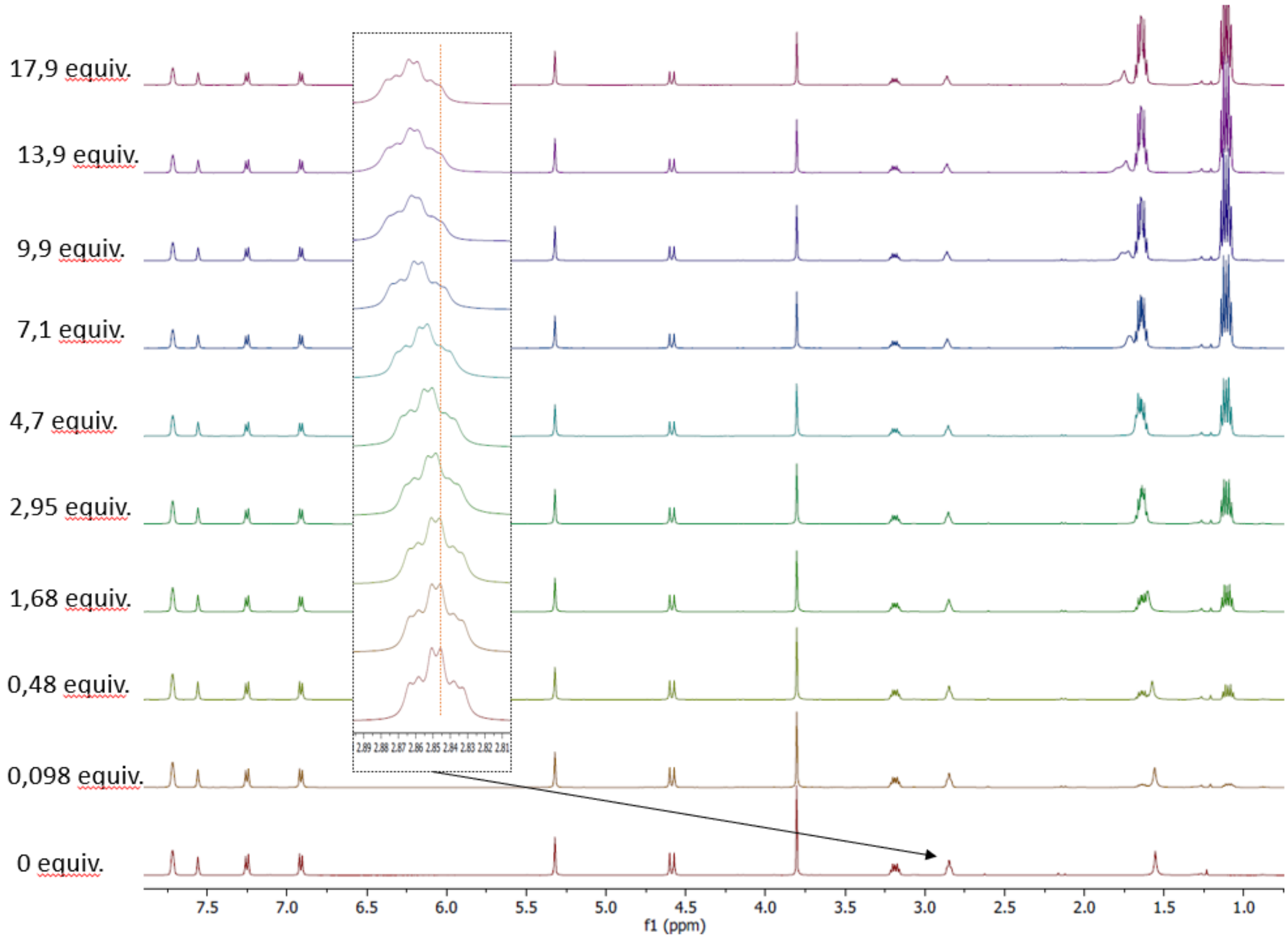

Figure S34 : ^{1}H NMR (CD_2Cl_2, 500 MHz, 300 K) spectra of [**2-Cl^{+}.BarF^{-}**] = 5 mM for several additions of a concentrated solution [TEPO] = 50 mM.

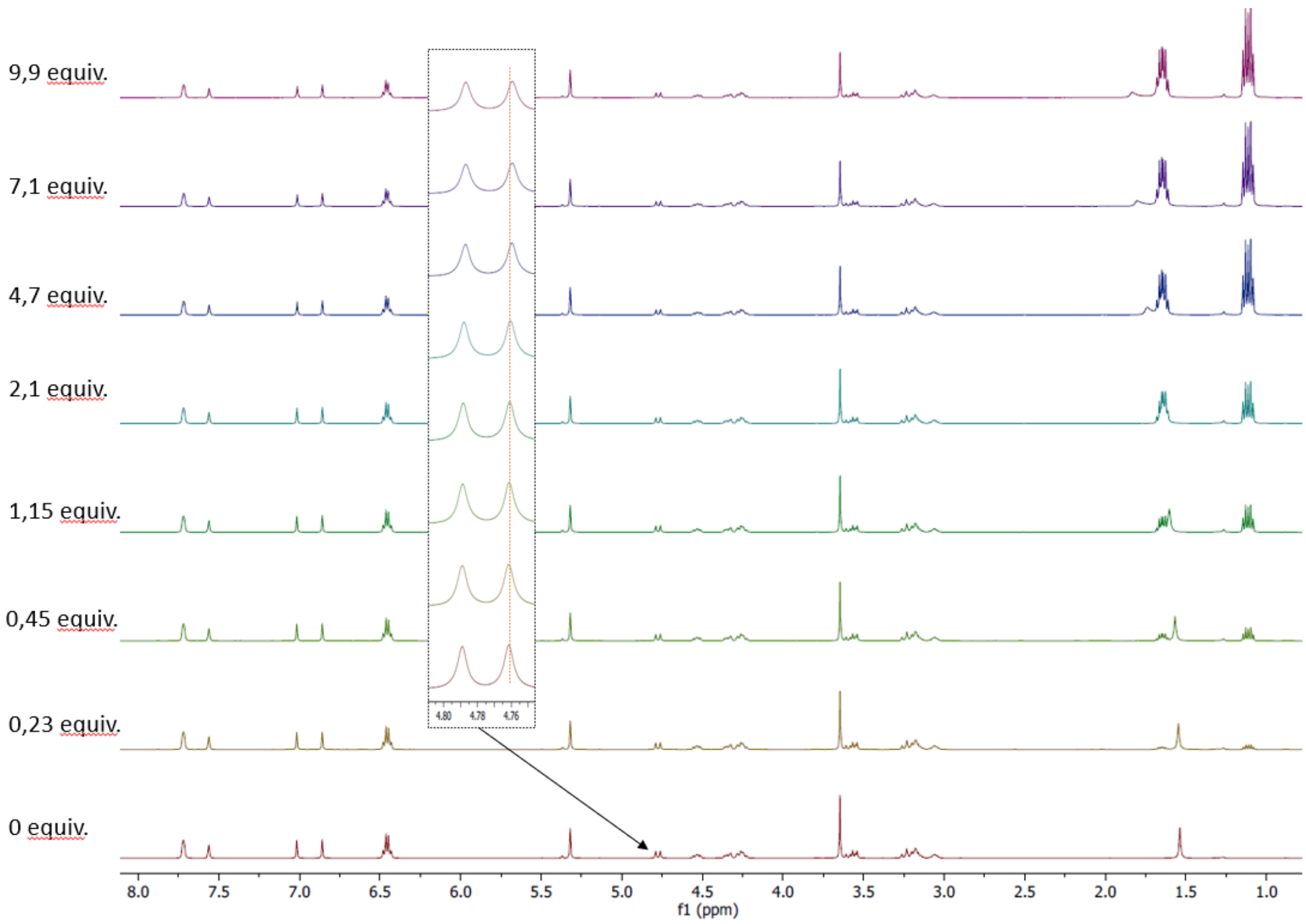

Figure S35 : ^{1}H NMR (CD_2Cl_2, 500 MHz, 300 K) spectra of [**1-H^{+}.BarF^{-}**] = 5 mM for several additions of a concentrated solution [TEPO] = 50 mM.

7. Triphenylphosphine oxide (TPPO) used as guest

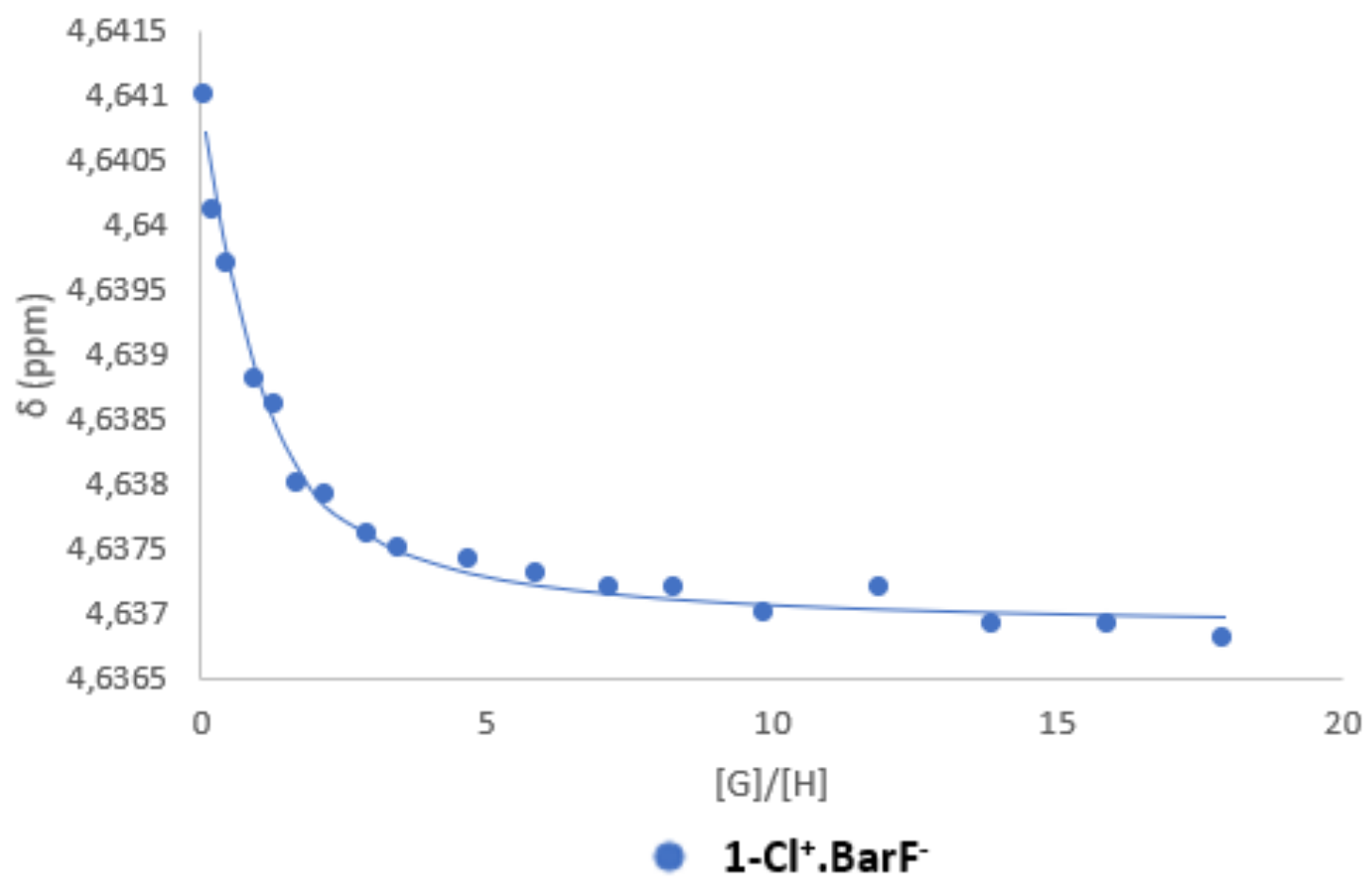

Figure S36 : Titration curve of host **1-Cl$^+$.BarF$^-$** with TPPO. The chemical shift changes Δδ of host's protons at 4.6411 ppm were measured and plotted as a function of the ratio [G]/[H] (dots). Curve was fitted with the bindfit program (line).

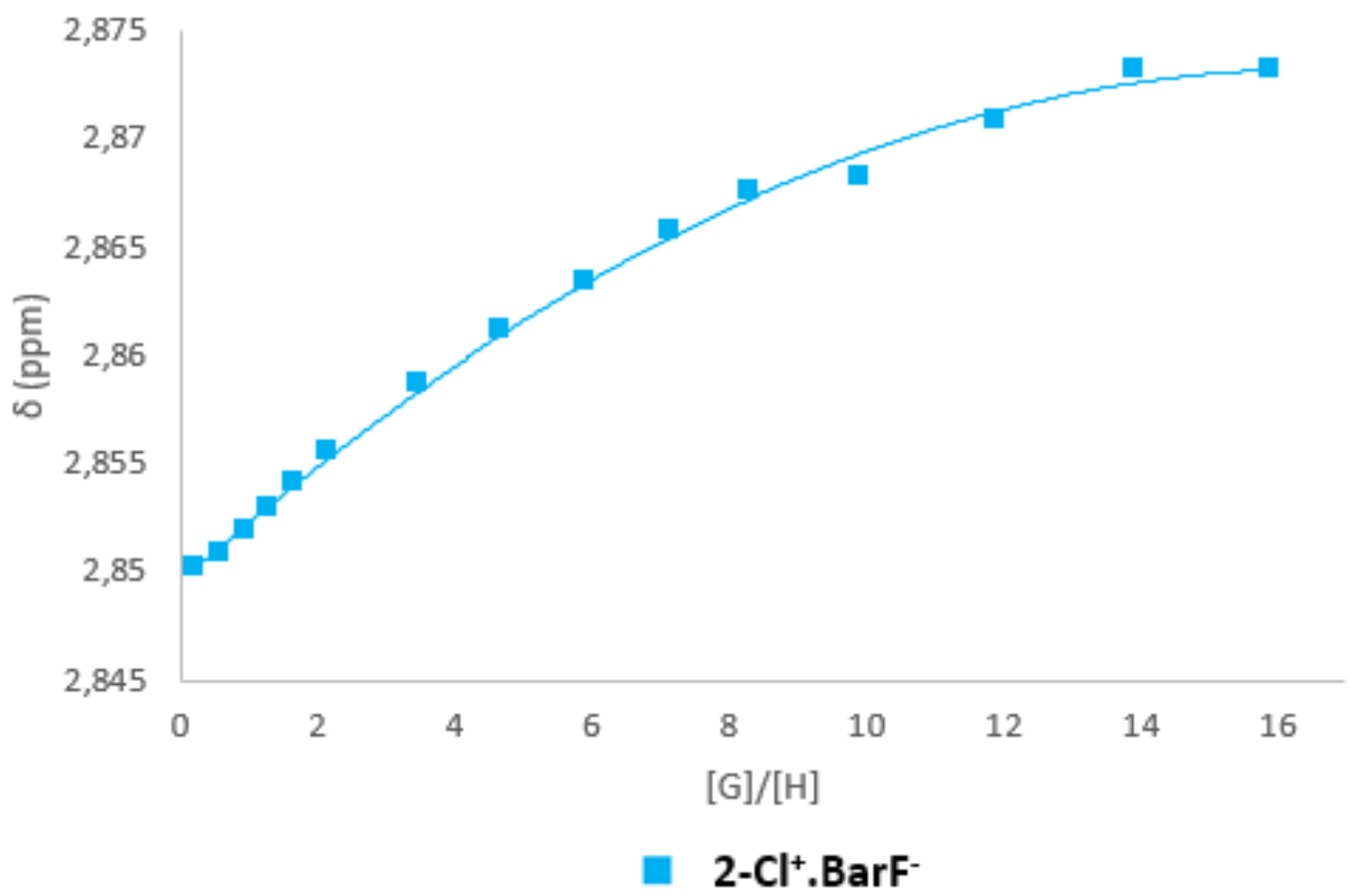

Figure S37 : Titration curve of host **2-Cl$^+$.BarF$^-$** with TPPO. The chemical shift changes Δδ of host's protons at 2.850 ppm were measured and plotted as a function of the ratio [G]/[H] (dots). Curve was fitted with the bindfit program (line).

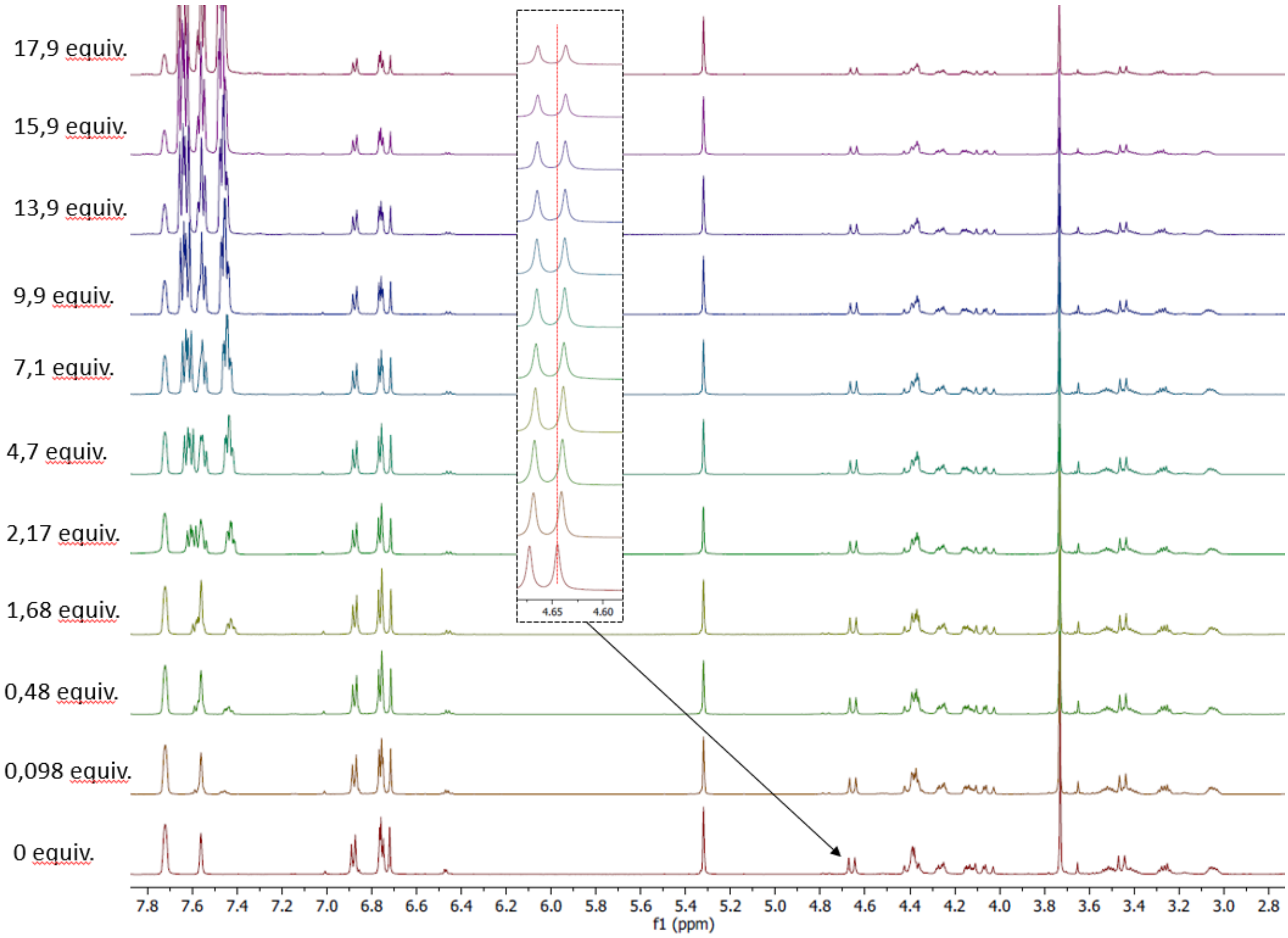

Figure S38 : ^{1}H NMR (CD_2Cl_2, 500 MHz, 300 K) spectra of [**1-Cl$^+$.BarF$^-$**] = 5 mM for several additions of a concentrated solution [TPPO] = 50 mM.

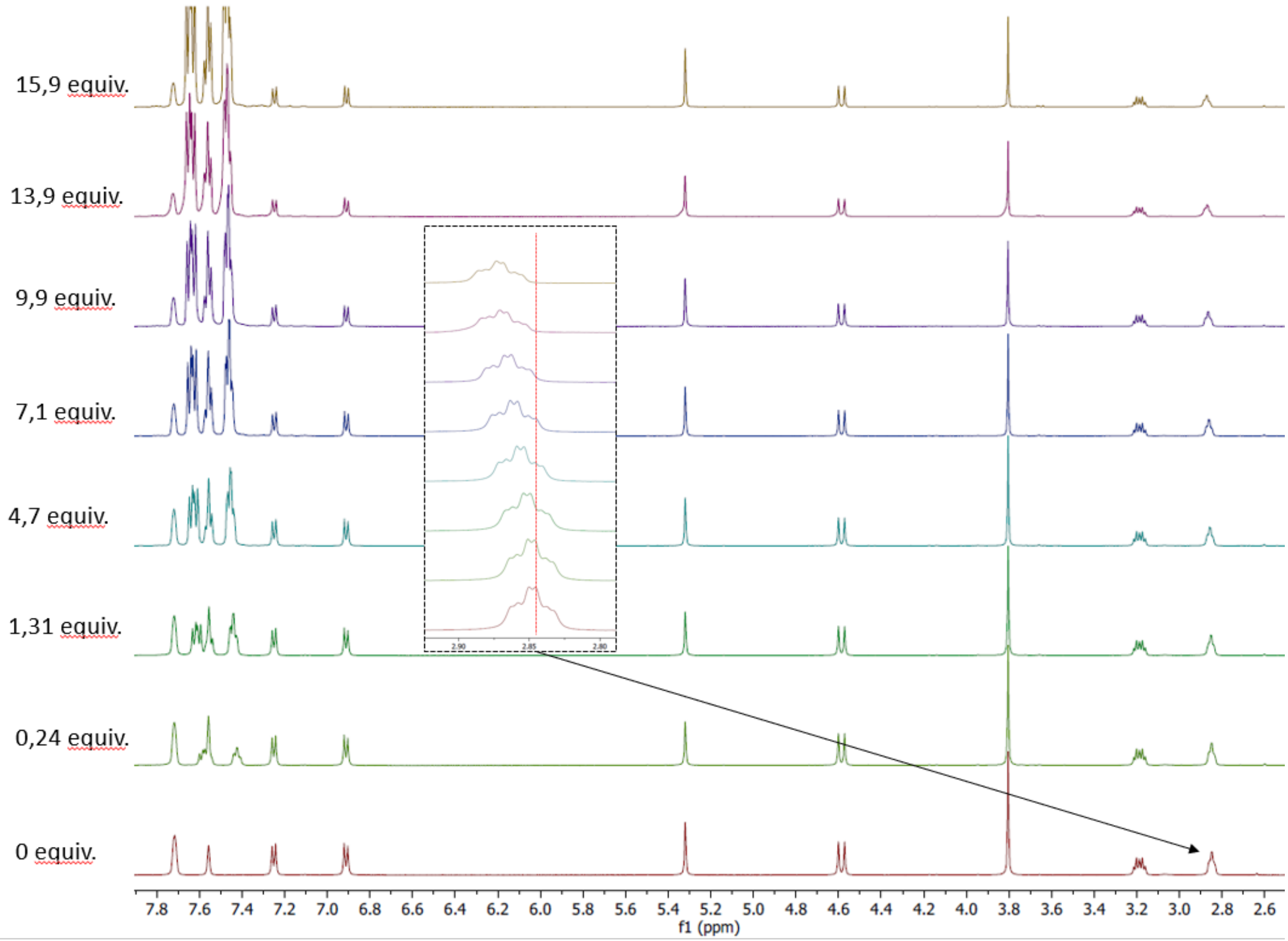

Figure S39 : ^{1}H NMR (CD_2Cl_2, 500 MHz, 300 K) spectra of [**2-Cl$^+$.BarF$^-$**] = 5 mM for several additions of a concentrated solution [TPPO] = 50 mM.

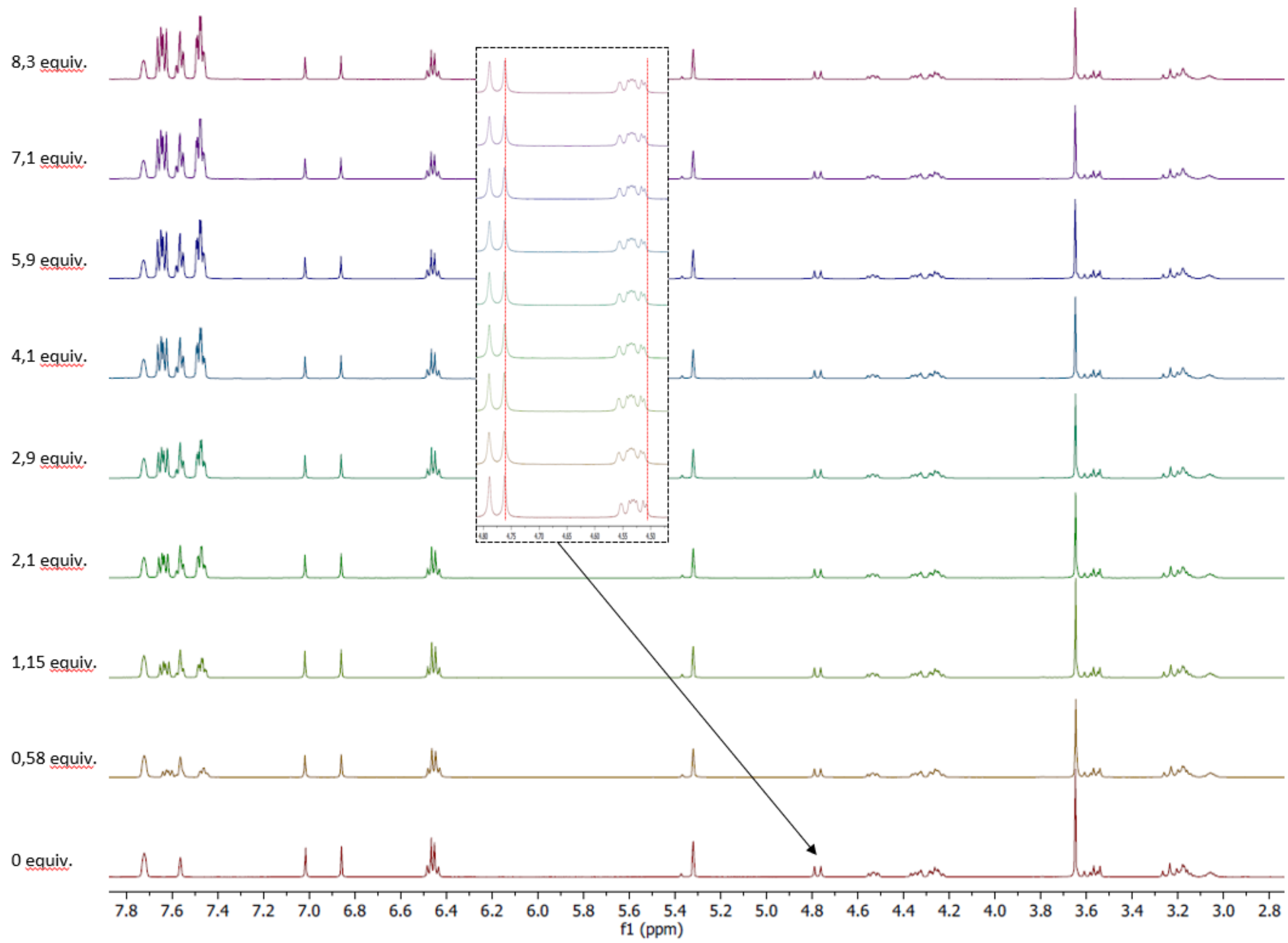

Figure S40 : ^{1}H NMR (CD_2Cl_2, 500 MHz, 300 K) spectra of [**1-H$^+$.BarF$^-$**] = 5 mM for several additions of a concentrated solution [TPPO] = 50 mM.

8. Dimethyl methylphosphonate (DMMP) used as guest

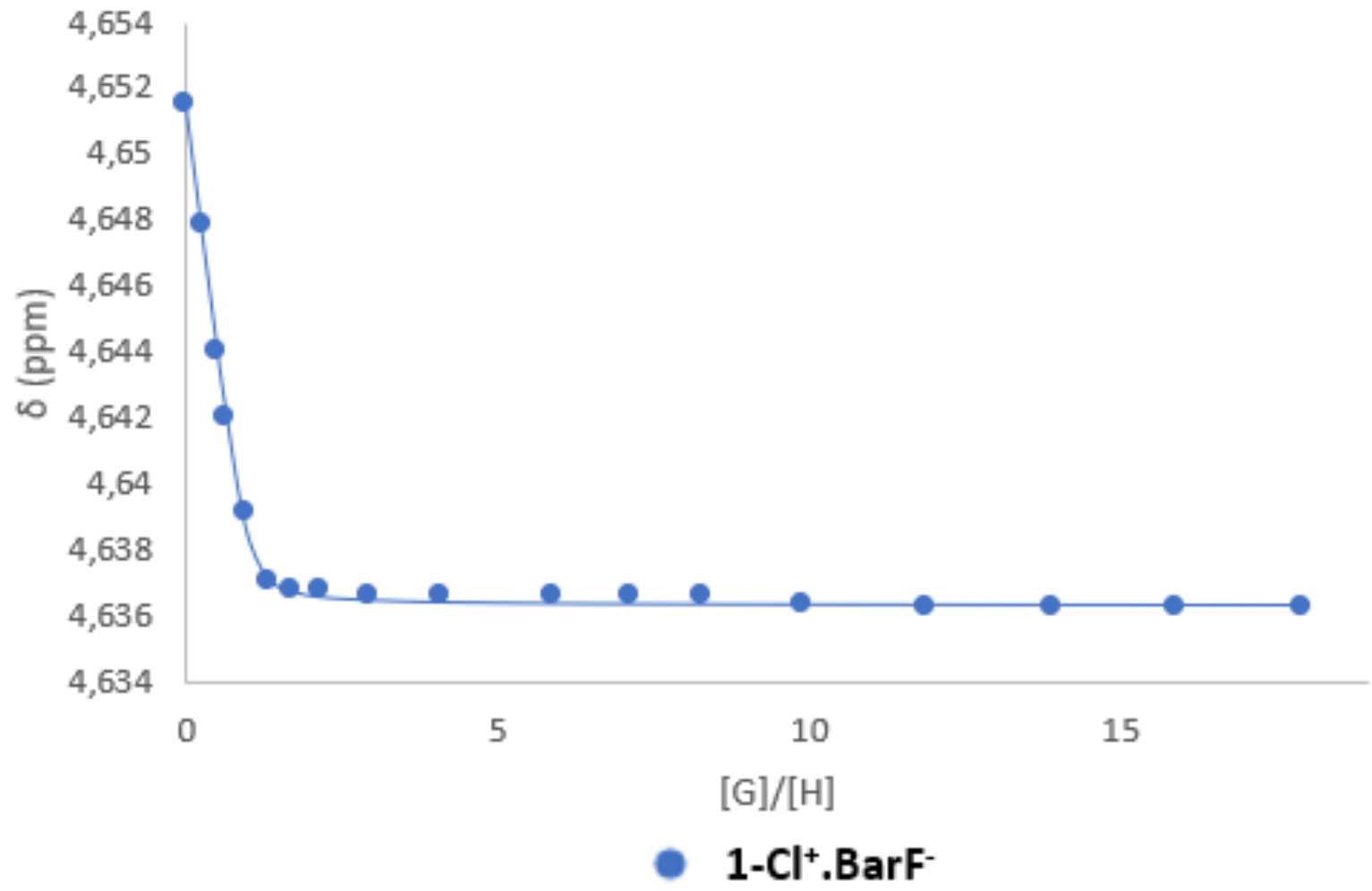

Figure S41 : Titration curve of host **1-Cl$^+$.BarF$^-$** with DMMP. The chemical shift changes Δδ of host's protons at 4.652 ppm were measured and plotted as a function of the ratio [G]/[H] (dots). Curve was fitted with the bindfit program (line).

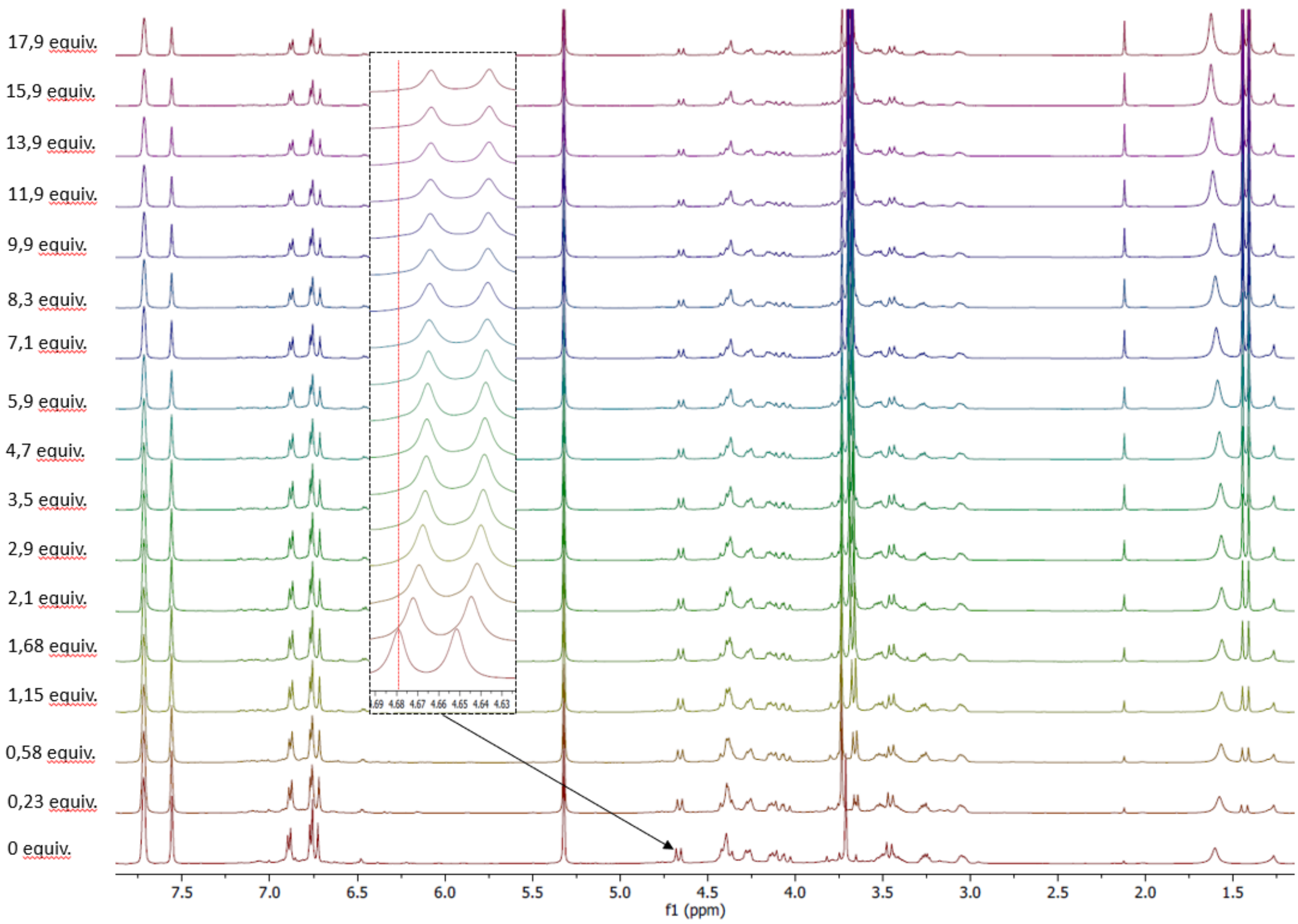

Figure S42 : ^{1}H NMR (CD_2Cl_2, 500 MHz, 300 K) spectra of [**1-Cl$^+$.BarF$^-$**] = 5 mM for several additions of a concentrated solution [DMMP] = 50 mM.

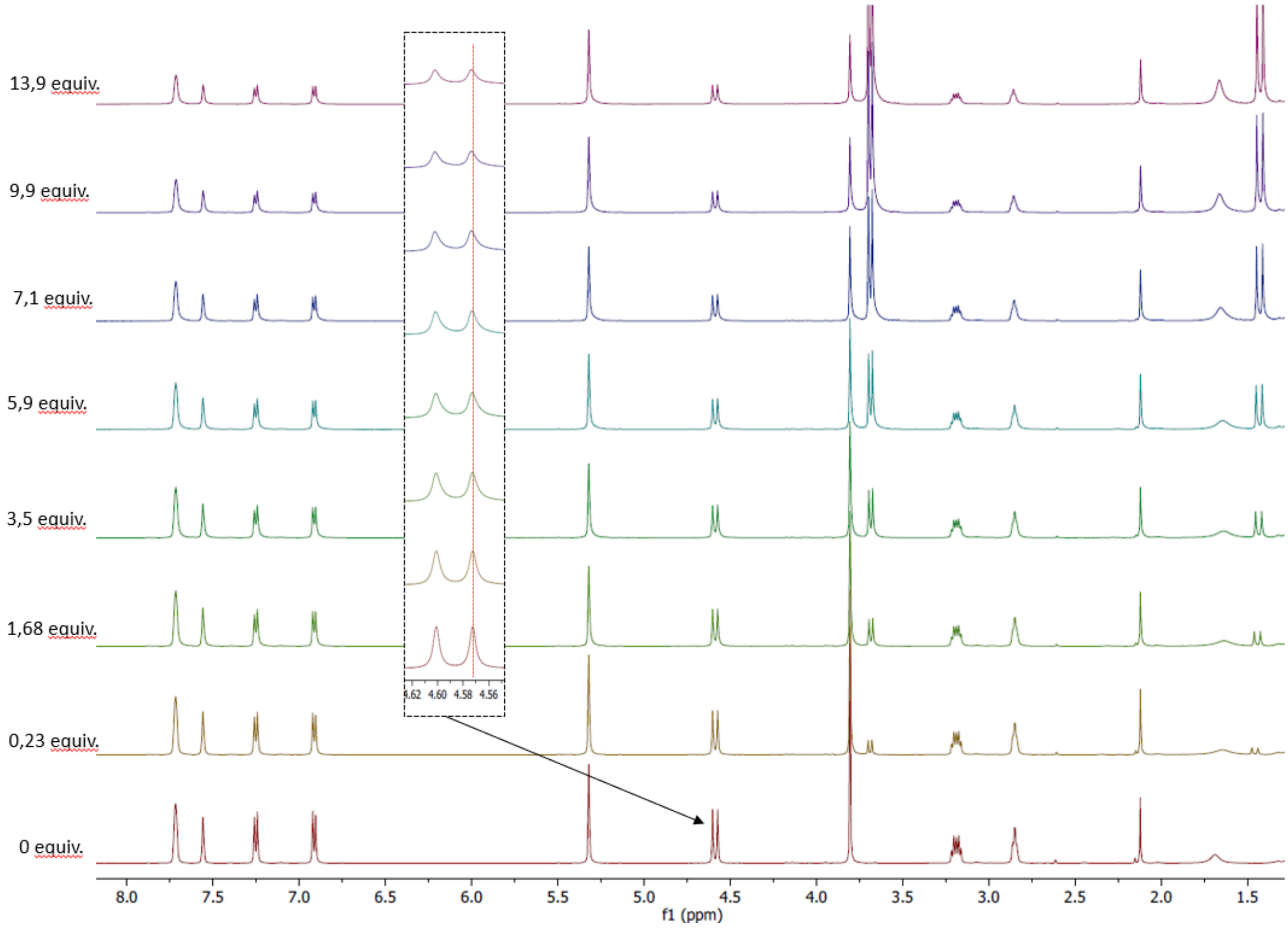

Figure S43 : ^{1}H NMR (CD_2Cl_2, 500 MHz, 300 K) spectra of [**2-Cl⁺.BarF⁻**] = 5 mM for several additions of a concentrated solution [DMMP] = 50 mM.

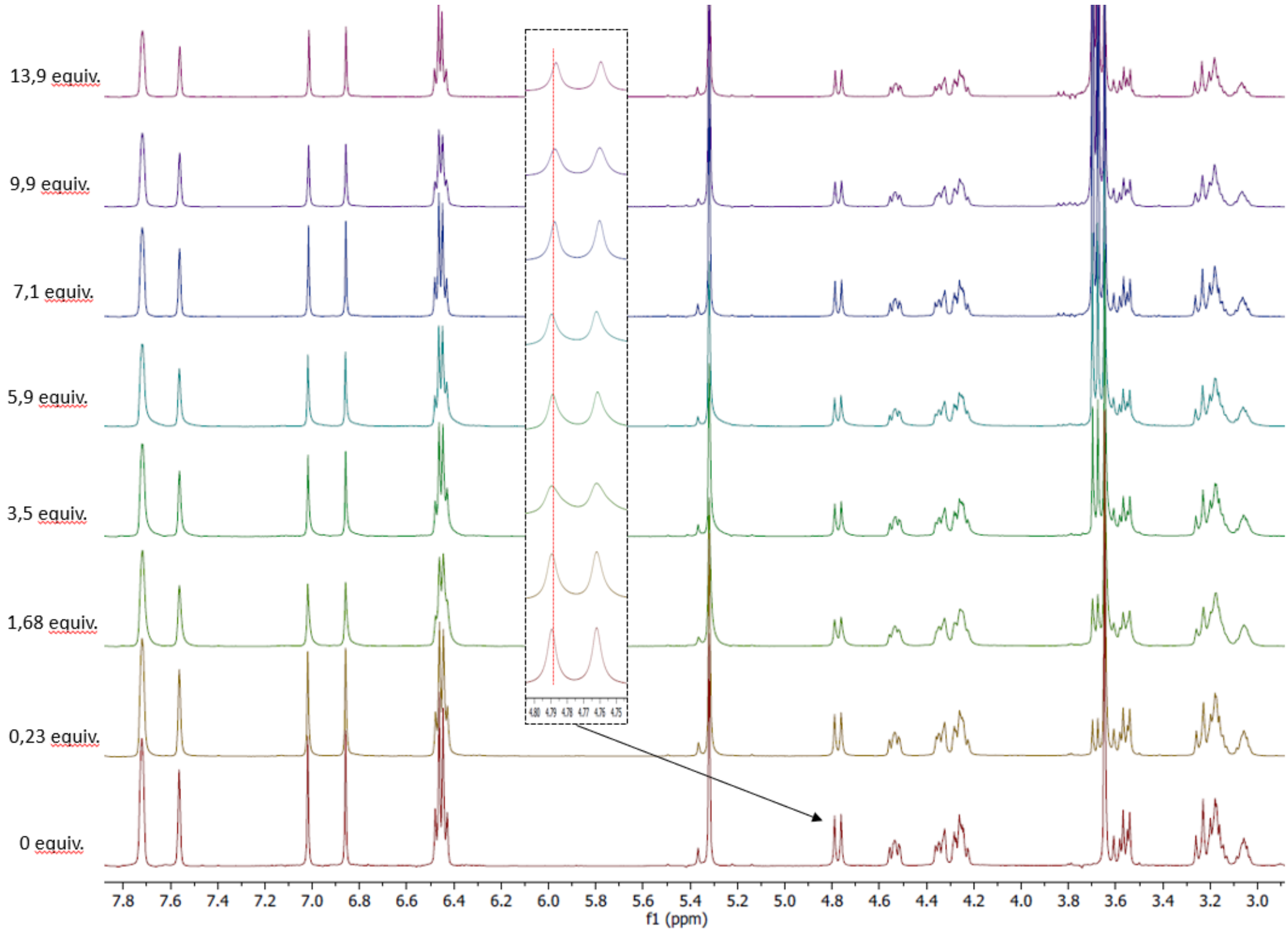

Figure S44 : ^{1}H NMR (CD_2Cl_2, 500 MHz, 300 K) spectra of [**1-H$^+$.BarF$^-$**] = 5 mM for several additions of a concentrated solution [DMMP] = 50 mM.

9. ^{1}H NMR titration at lower concentrations with Dimethyl methylphosphonate (DMMP) as guest

Titration curve of host **1-Cl$^+$.BarF$^-$** with DMMP. The chemical induced shifts δ of host's protons at 4.6519 ppm were measured and plotted as a function of the ratio [G]/[H] (dots). Curve was fitted with the bindfit program (line).

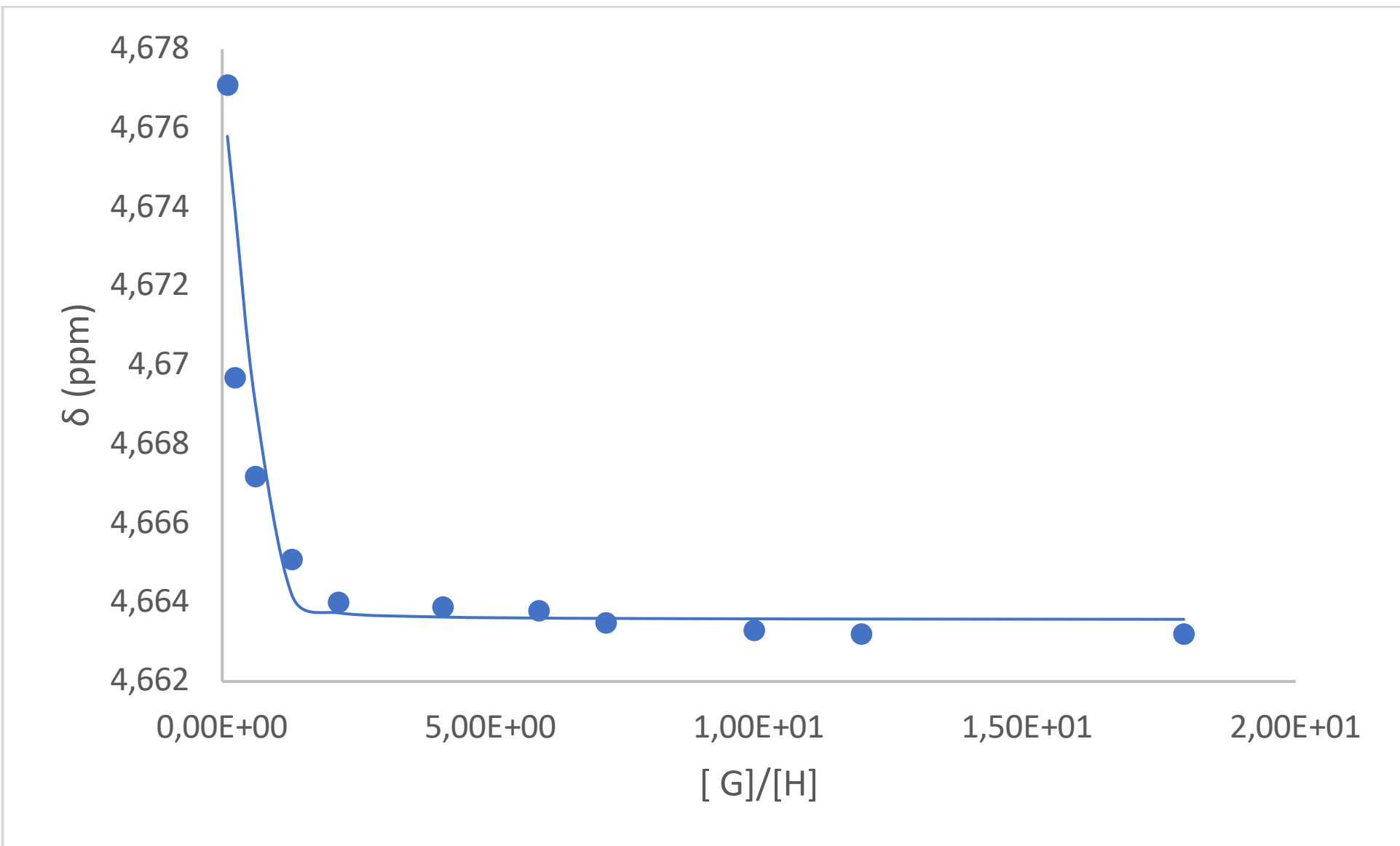

Figure S45 : Titration curve of host **1-Cl$^+$.BarF$^-$** with DMMP. The chemical induced shifts δ of host's protons at 4.6771 ppm were measured and plotted as a function of the ratio [G]/[H] (dots). Curve was fitted with the bindfit program (line). K_a = 10 259 +/- 767 M^{-1}

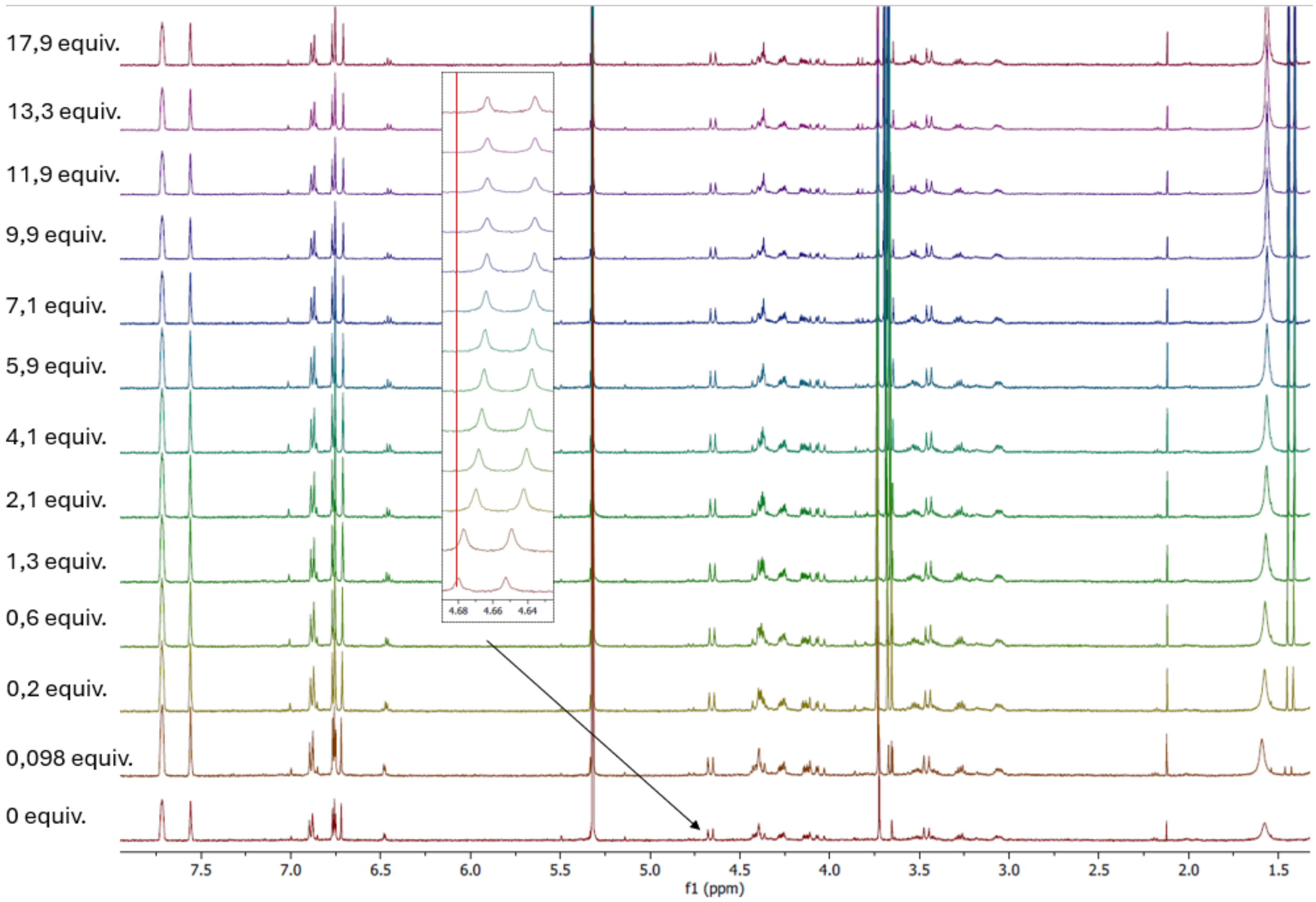

Figure S46 : ^{1}H NMR (CD_2Cl_2, 500 MHz, 300 K) spectra of [**1-Cl⁺.BarF⁻**] = 2 mM for several additions of a concentrated solution [DMMP] = 20 mM.

VII/ Variable-temperature ^{1}H NMR titrations

A solution of host **1-Cl⁺.BarF⁻** (5.0 mM in CD_2Cl_2, 500 µL) was titrated in NMR tubes with aliquots of a concentrated solution (50 mM in CD_2Cl_2) of DMMP at different temperatures (300 K, 288 K and 273 K) using a 600 MHz NMR spectrometer. The chemical shifts of protons of the host were measured after each addition and plotted as a function of the guest/host ratio ([G]/[H]). The association constant K_a was obtained by nonlinear least-squares fitting of these plots using the BINDFIT program.

T (Kelvin)	K_a (M^{-1})	1/T	ln(K_a)
300	10137	0,0033	9,22
288	7230	0,0035	8,88
273	6496	0,0037	8,77

Table S1: Measurement of K_a at different temperatures

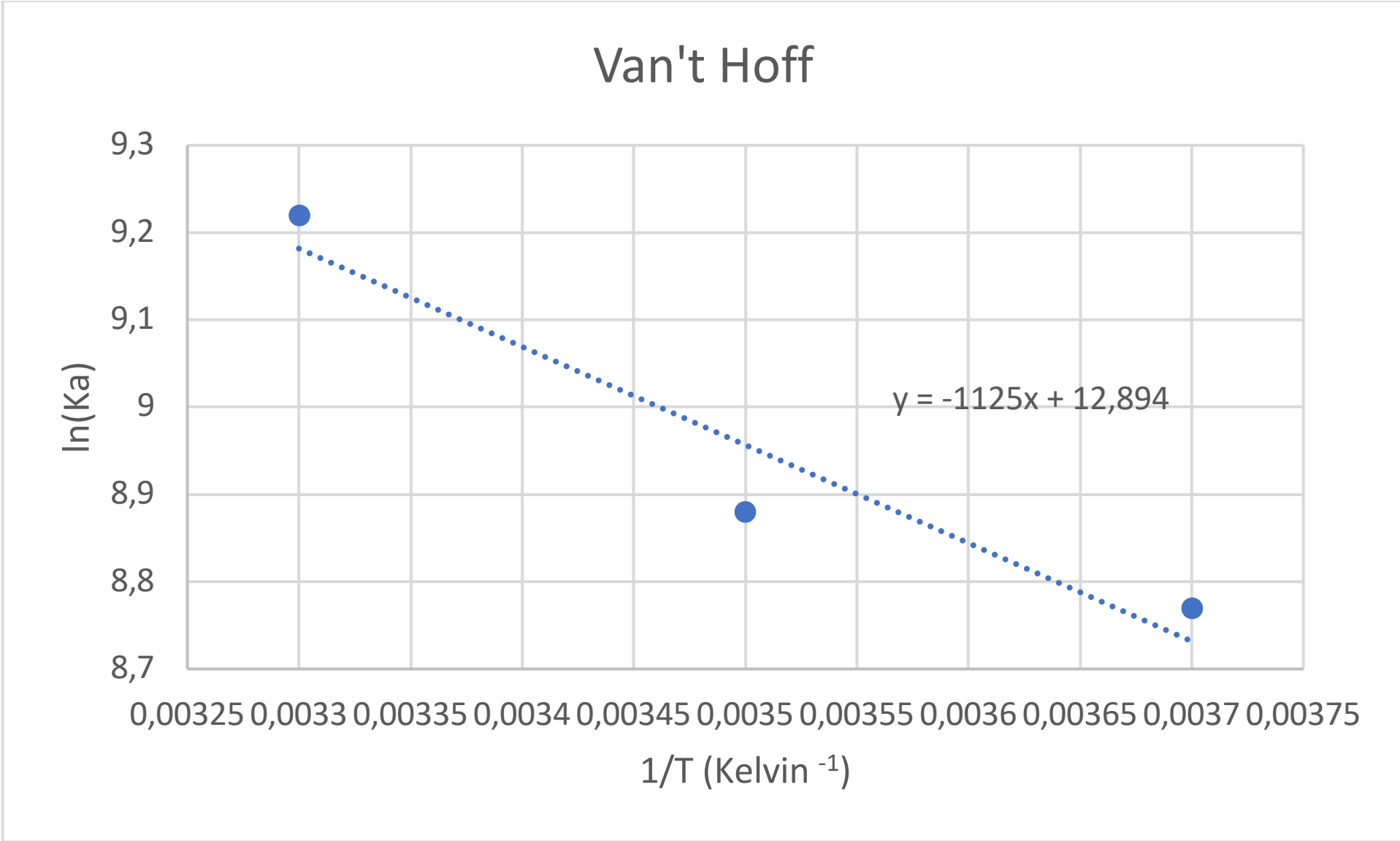

Figure S47 : Van't Hoff equation

$$\ln K_a = -\frac{\Delta H}{R}\frac{1}{T} + \frac{\Delta S}{R}$$

Calculation of ΔH, ΔS and ΔG:

R = 8.314 J.mol^{-1}.K^{-1}

ΔH = -R x slope = 9.35 kJ.mol^{-1}

ΔS = R x intercept = 107 J.K^{-1}.mol^{-1}

ΔG = -22.5 kJ.mol^{-1}

VIII/ DOSY experiments

DOSY experiments are performed on a 5mM solution of the DMMP@**1-Cl$^+$.BarF$^-$** complex in CD_2Cl_2, as well as on a 5mM solution of DMMP in CD_2Cl_2.

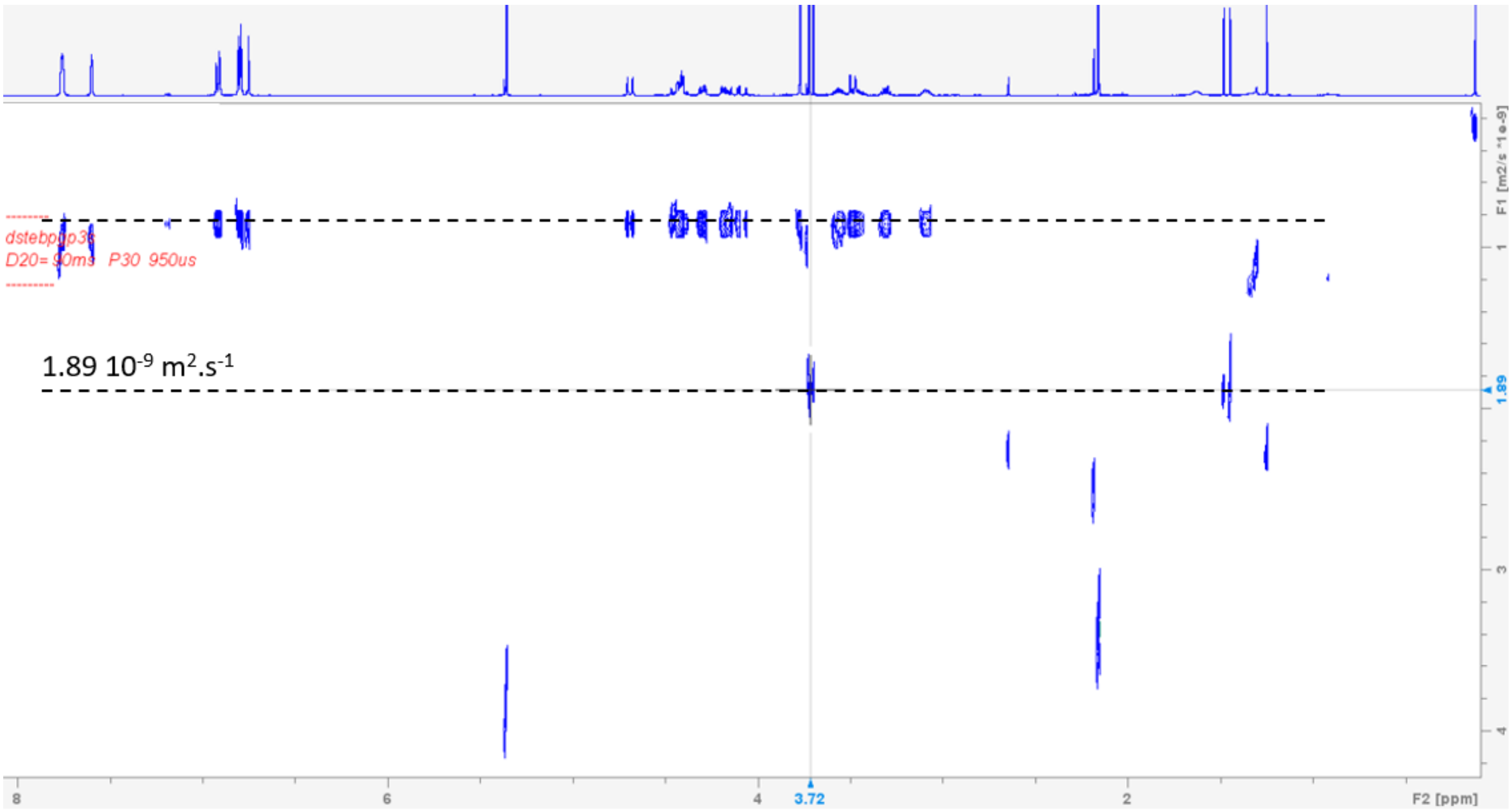

Figure S48 : DOSY experiment (CD_2Cl_2, 500 MHz, 300 K) of the DMMP@**1-Cl⁺.BarF⁻** complex (5mM).

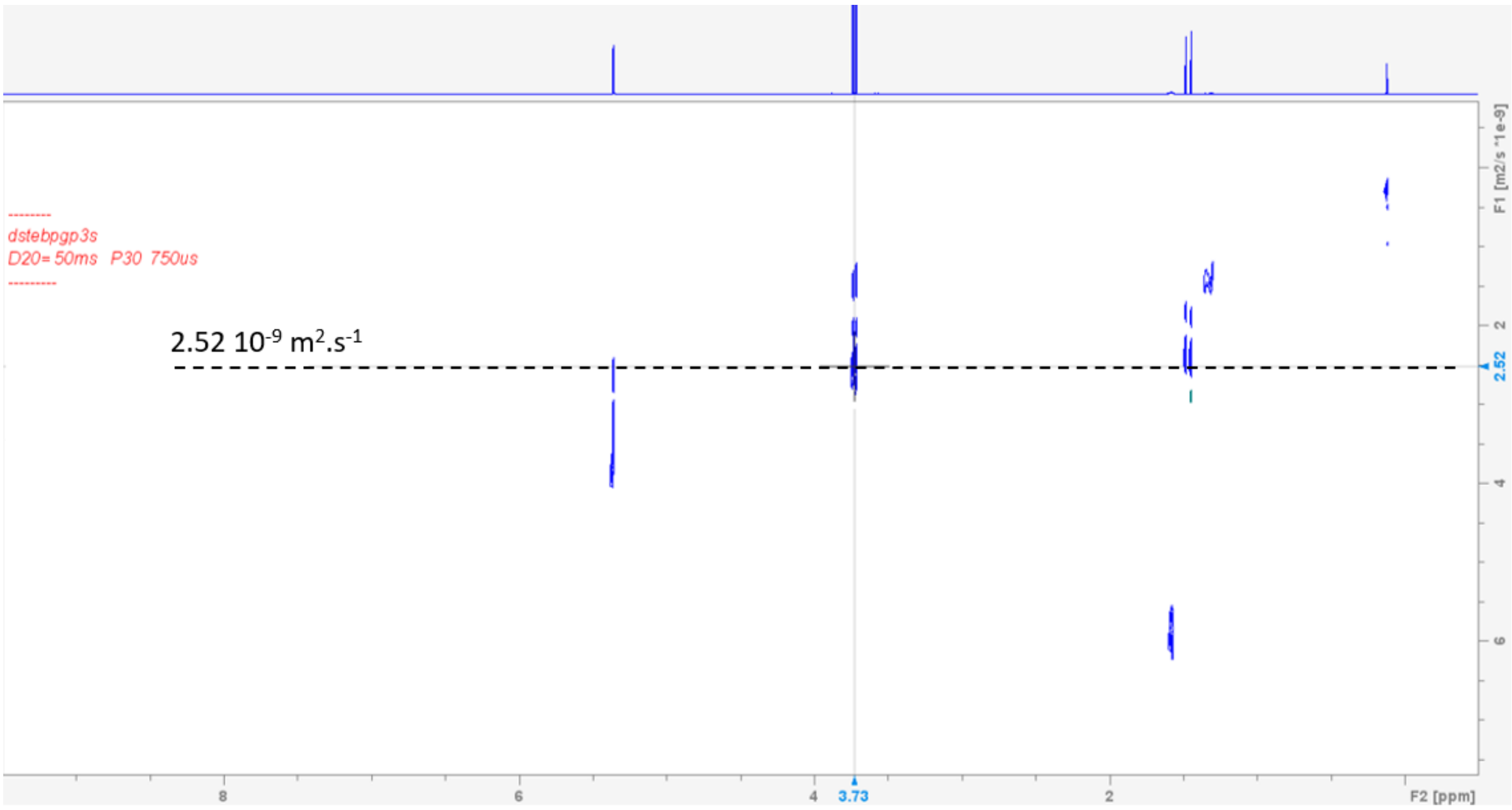

Figure S49 : DOSY experiment (CD_2Cl_2, 500 MHz, 300 K) of DMMP (5mM).

IX/ Stability studies

500 µL of a 5mM solution of **1-Cl⁺.Cl⁻** and **2-Cl⁺.Cl⁻** in CD_2Cl_2 are prepared in 1 mL vials.. Each solution is transferred into a two-compartment NMR tube containing 100 µL of water. The inner compartment contained a 100 mM solution of

TBAPF$_6$ in CHCl$_3$, used as an external reference. The timer was launched after a quick shaking of the NMR tube, and a ^{31}P NMR spectrum (lasting approximately 22 minutes) was recorded every 30 minutes for 15 hours.

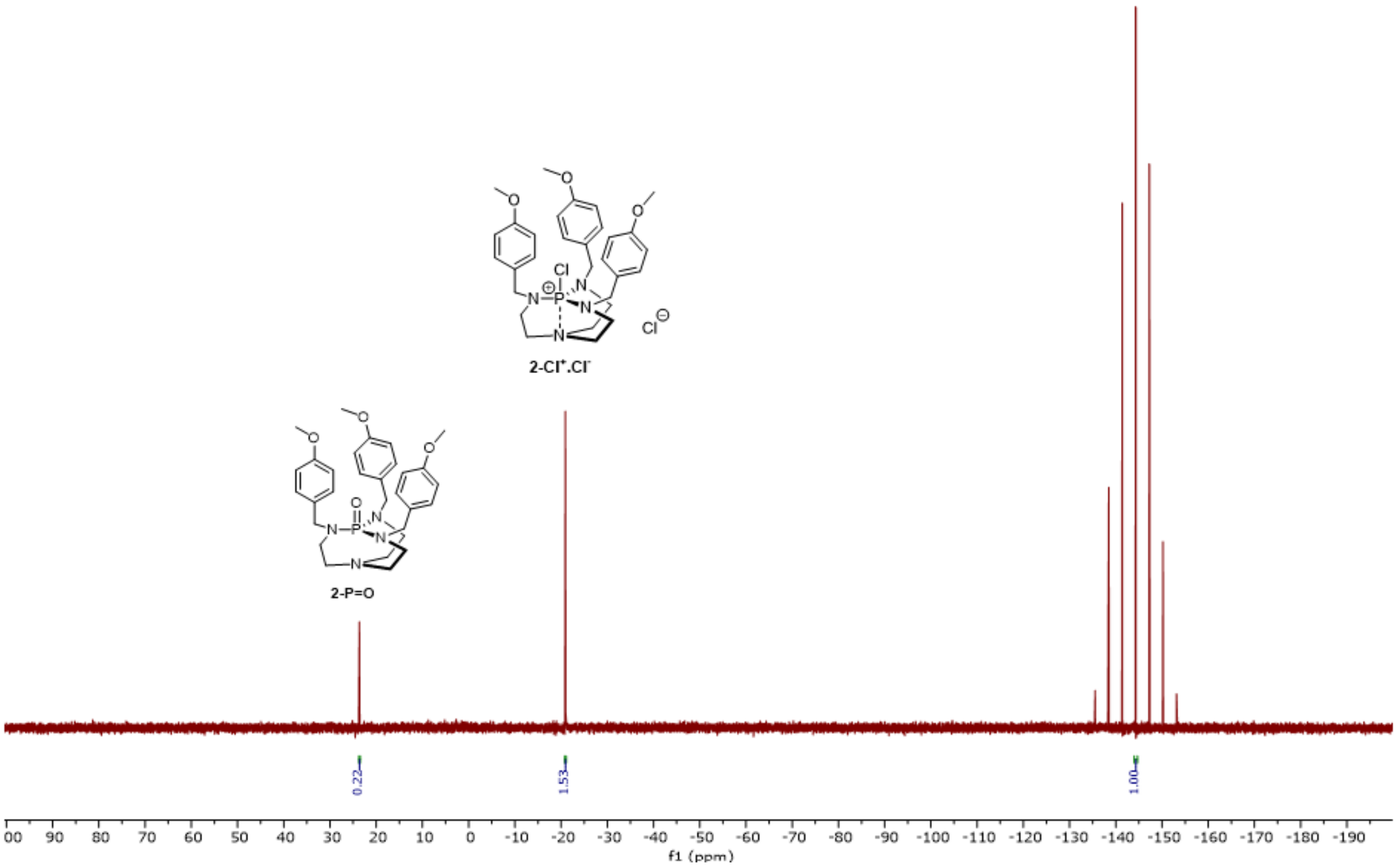

Figure S50 : ^{31}P NMR (CD$_2$Cl$_2$, 200 MHz, 300 K) spectrum of **2-Cl$^+$.Cl$^-$**, 30 minutes after H$_2$O addition.

The area of the highest peak of the PF$_6^-$ anion (integrated between -144.0 and -144.7 ppm) was set to 1.0, and the area of the peak corresponding to **2-Cl$^+$.Cl$^-$** (between -20.80 and -21.1 ppm) was integrated**.** The concentration of **2-Cl$^+$.Cl$^-$**, normalized to one, was plotted.

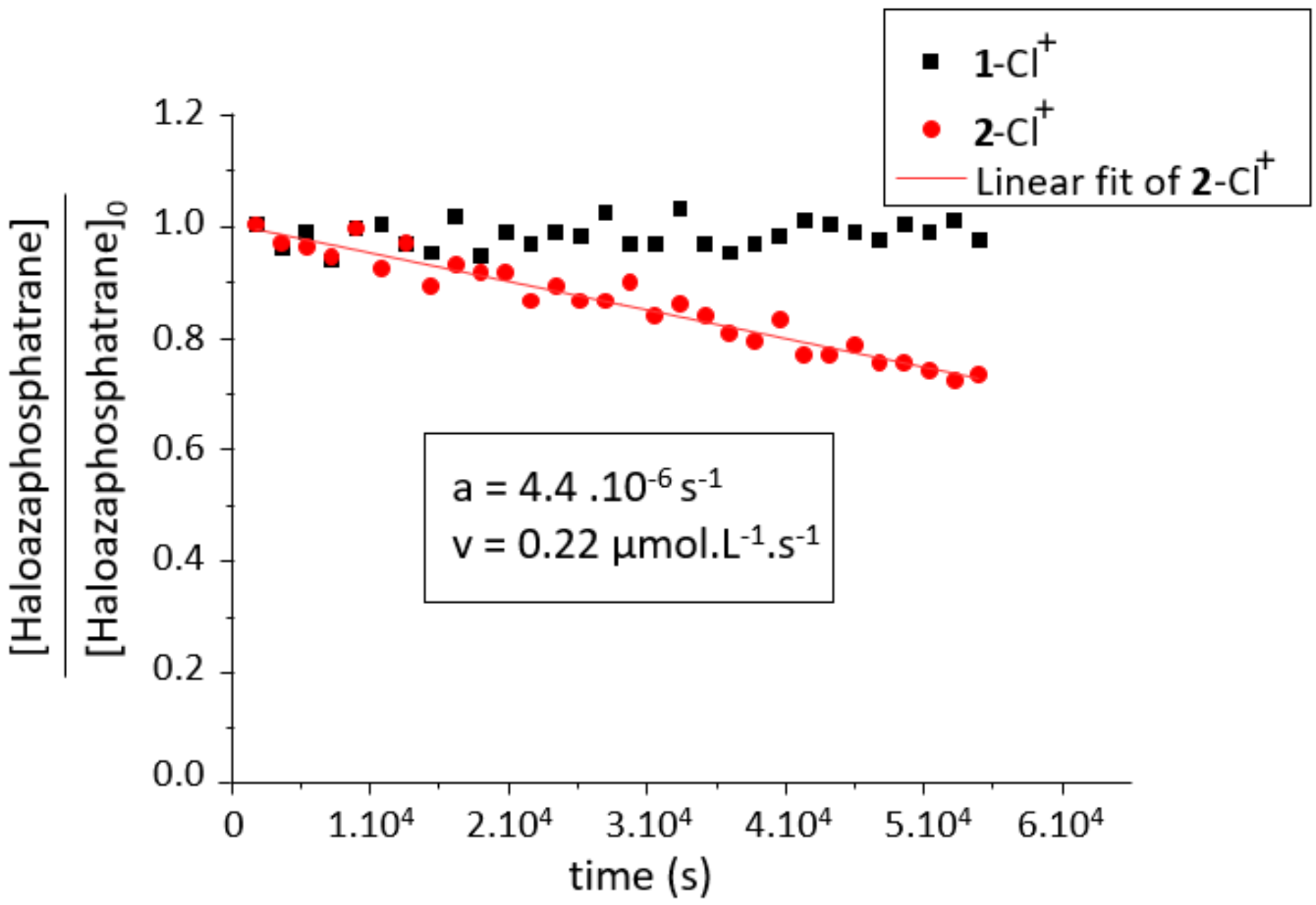

Figure S51 : Concentration of **1-Cl$^+$.Cl$^-$** and **2-Cl$^+$.Cl$^-$** as a function of reaction time

Taking into account the initial proportion of **2-P=O** byproduct, the initial concentration of **2-Cl$^+$.Cl$^-$** is estimated around 4.4 mM. Hence the initial rate of the reaction, estimated around 0.22 µmol.L^{-1}.s^{-1}.

As this experiment did not allow the degradation rate of **1-Cl⁺** to be estimated, an additional experiment was performed: A 500 µL aliquot of a 5 mM solution of **1-Cl⁺·BArF⁻** in CD_2Cl_2 was prepared in a 1 mL vial and transferred to the outer compartment of a two-compartment NMR tube containing 100 µL of water. The inner compartment contained a 100 mM solution of $TBAPF_6$ in $CHCl_3$, used as an external reference. The NMR tube was briefly shaken, after which the timer was started. A ^{31}P NMR spectrum (acquisition time: approximately 22 min) was recorded over several days.

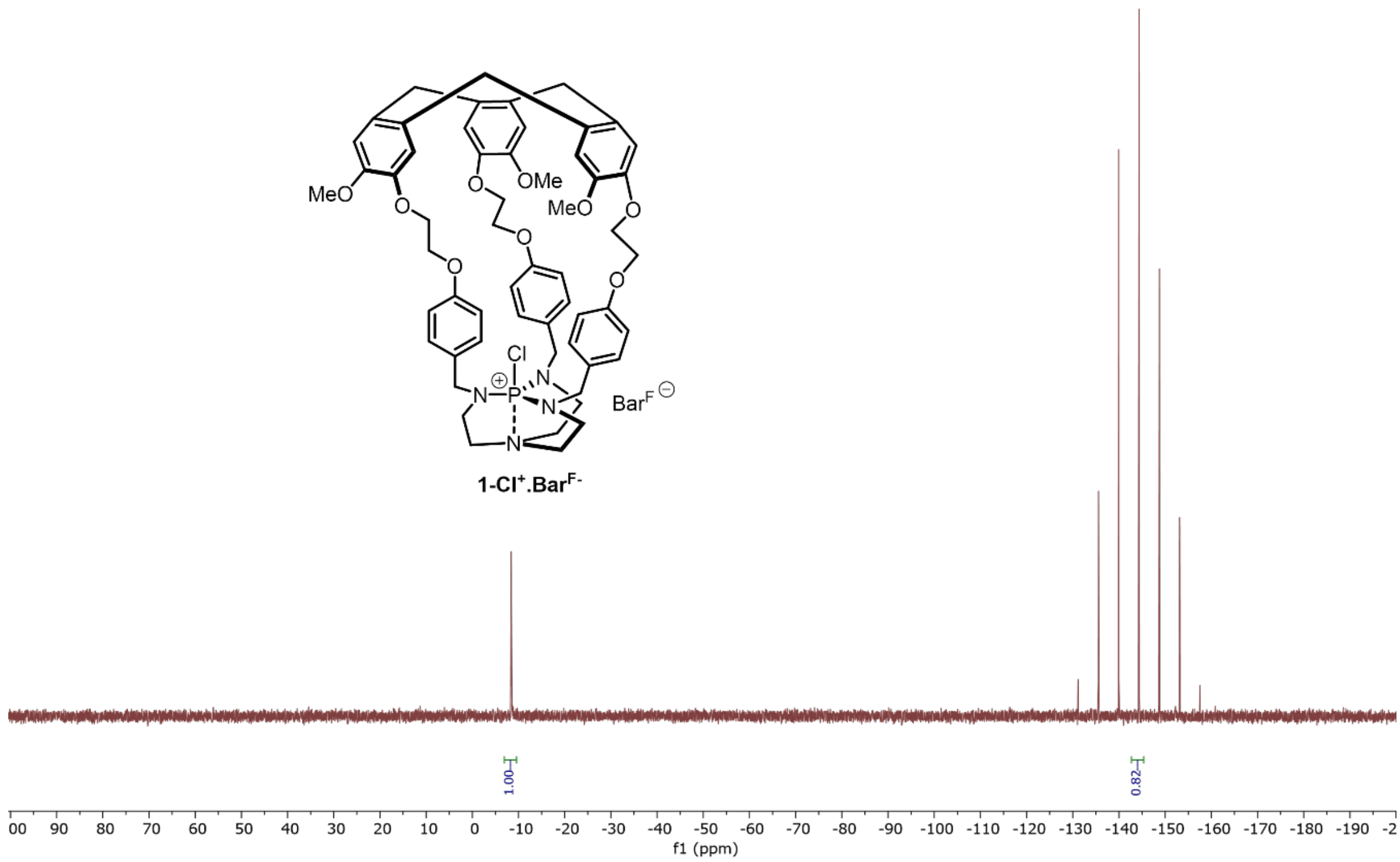

Figure S52 : ^{31}P NMR (CD_2Cl_2, 200 MHz, 300 K) spectrum of **1-Cl⁺.BAr^F-**, 8 days after H_2O addition.

None of the experiments performed provided evidence for the formation of degradation products of cage **1-Cl⁺·BArF⁻**, particularly the ^{31}P NMR analysis performed after 8 days. Assuming that up to 10% of the corresponding phosphine oxide **1-P=O** could have been formed without being detected by ^{31}P NMR spectroscopy, a maximum degradation rate of 7 10^{-4} µmol.L^{-1}.s^{-1} can be estimated.

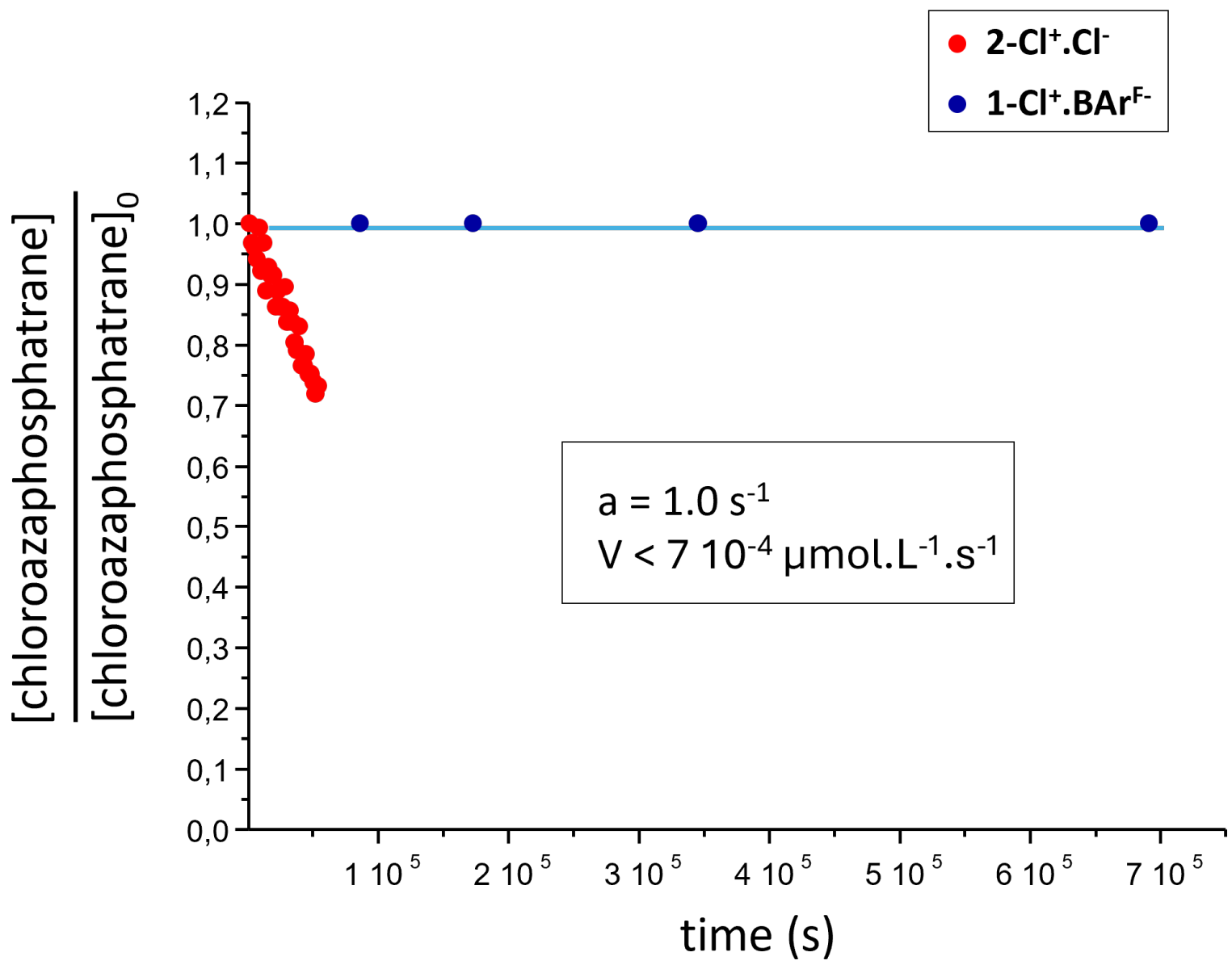

Figure S53 : Concentration of **2-Cl⁺.Cl⁻** and **1-Cl⁺.BArF⁻** versus reaction time

X/ Computational approach

Calculations were performed with the TURBOMOLE program package.[4] Geometry optimizations were performed at the PBE0-D3/def2-SVP level of theory,[5] by using the COSMO implicit solvent model parametrized for CH_2Cl_2.[6] In the following we report the optimized structure coordinates, concerning the encapsulation of DMMP into either the **1-Cl⁺** or the **1-H⁺** hemicryptophane.

Furthermore, volumes were calculated using the MoloVol software in two-probes mode with a grid resolution of 0.1 Å, a small probe radius of 0.9 Å and a large probe radius of 2.2 Å (crystal structures) or 2.6 Å (computed structures).[7] For the computed systems, the cavities were evaluated by considering the optimized structure obtained with the DMMP molecule inside and by removing the guest (estimated volume of the DMMP: 110 $Å^3$). Reports of the calculations are available below. Images were rendered with UCSF ChimeraX, developed by the Resource for Biocomputing, Visualization, and Informatics at the University of California, San Francisco, with support from NIH R01-GM129325.[2][8]

Volume data

Parameters

Crystal unit cell analysis: No

[4] TURBOMOLE V7.5 2020, a development of University of Karlsruhe and Forschungszentrum Karlsruhe GmbH, 1989-2007, TURBOMOLE GmbH, since 2007; available from http://www.turbomole.com.

[5] a) P. A. M. Dirac, *Proc. Royal Soc. Lond. A* **1929**, *123*, 714-733; b) J. C. Slater, *Phys. Rev.* **1951**, *81*, 385-390; c) J. P. Perdew and Y. Wang, *Phys. Rev. B* **1992**, *45*, 13244-13249; d) J. P. Perdew, K. Burke and M. Ernzerhof, *Phys. Rev. Lett.* **1996**, 77, 3865-3868; e) S. Grimme, J. Antony, S. Ehrlich, H. Krieg, J. *Chem. Phys*. **2010**, *132*, 154104. f) F. Weigend, and R. Ahlrichs, *Phys. Chem. Chem. Phys*. **2005**, *7*, 3297-3305.

[6] A. Klamt and G. Schuurmann, *J. Chem. Soc.-Perkin Trans.* **1993**, 2, 799-805.

[7] J. B. Maglic and R. Lavendomme, MoloVol: an easy-to-use program for analyzing cavities, volumes and surface areas of chemical structures. *J. Appl. Cryst*., **2022**, 55, 1033-1044. DOI: 10.1107/S1600576722004988.

[8] E.C. Meng, T. D. Goddard, E. F. Pettersen, G. S. Couch, Z. J. Pearson, J. H. Morris, and al. UCSF ChimeraX: Tools for structure building and analysis. *Protein Science*, **2023**, 32(11):e4792. https://doi.org/10.1002/pro.4792

Probe mode: two probes

Small probe radius: 0.9 Å

Large probe radius: 2.2 or 2.6 Å

Grid resolution: 0.1 Å

Optimization depth: 4

Elements radii (Å):

P	O	N	H	Cl	C
1.9	1.5	1.66	1.2	1.82	1.77

Cavities data

Cavity ID	Occupied Volume ($Å^3$)	Accessible Volume ($Å^3$)	Cavity Type	Cavity center coordinates (Å)		
				x	y	z
1-Cl+ (crystal structure)						
1	171.269	37.126	Tunnel	5.35	12.4	16.95
1-H+ (crystal structure)						
1	117.17	28.774	Pocket	2.7	6.35	3.55
1-Cl+ (DFT structure)						
1	206.059	60.113	Tunnel	-0.5	0.25	-0.3
1-H+ (DFT structure)						
1	219.856	66.198	Tunnel	0.0	0.05	-0.5

Coordinates in xyz format

1-Cl$^+$@DMMP

```
151
Energy = -4587.2407644051
Cl    2.7056962   -0.1585242    0.0919457
P     4.7886683   -0.1911799    0.2213433
O     0.1074028   -4.0584530   -3.4814907
O    -2.4881331   -2.6930376   -3.4651924
O    -2.3465916   -0.2982647   -4.5339805
O     0.4217405    5.1629158   -1.5317603
O    -2.3855786    4.6617408   -0.8270819
O    -2.6992591    4.4773740    1.7681612
O    -2.9508004   -1.2453301    4.1479805
O    -0.3428782   -0.9099761    5.3097717
O    -2.9749461   -3.3737320    2.6327967
N     6.7749954   -0.2152358    0.3602482
N     4.8402244    0.5708612    1.7345833
N     4.9290511   -1.8780300    0.1612199
N     5.0553549    0.7119066   -1.1844990
C     7.1972910   -1.3753693   -0.4385658
H     8.2378763   -1.6440787   -0.2088489
H     7.1389352   -1.0983557   -1.4989049
C     6.2204801   -2.4848412   -0.1109697
H     6.1556683   -3.1872089   -0.9572684
H     6.5566095   -3.0687601    0.7642745
C     3.8856675   -2.8098459    0.5634612
H     3.3051359   -2.3899954    1.3986745
H     4.3995462   -3.6925746    0.9804566
C     2.9320826   -3.2408964   -0.5272886
C     1.7370347   -3.8707054   -0.1804427
```

H	1.5250354	-4.0860645	0.8712299
C	0.7756676	-4.1977996	-1.1368919
H	-0.1541578	-4.6652699	-0.8118070
C	1.0010502	-3.8832779	-2.4815092
C	2.2286018	-3.3154243	-2.8487666
H	2.4017602	-3.0890018	-3.9031579
C	3.1726862	-2.9919297	-1.8821438
H	4.1011510	-2.5008726	-2.1851196
C	-1.1466575	-4.6549100	-3.2325472
H	-1.0361330	-5.5647963	-2.6175664
H	-1.5201948	-4.9656414	-4.2188543
C	-2.1781628	-3.7535145	-2.5908191
H	-3.0622679	-4.3861226	-2.3930345
H	-1.8297211	-3.3335666	-1.6305040
C	-3.3978857	-1.7814283	-3.0458149
C	-3.3245373	-0.4952518	-3.6270066
C	-4.2356454	0.4722982	-3.2230695
H	-4.1835846	1.4646948	-3.6708895
C	-5.1968758	0.2383506	-2.2204510
C	-5.2768649	-1.0381733	-1.6524012
C	-4.3799008	-2.0282196	-2.0963933
H	-4.4457879	-3.0225522	-1.6544365
C	-2.0405517	1.0235709	-4.9030473
H	-1.1455310	0.9745004	-5.5363627
H	-1.8223194	1.6423028	-4.0143668
H	-2.8562477	1.4937417	-5.4787209
C	-6.2039430	-1.3907886	-0.5032098
H	-7.0912895	-0.7463182	-0.5074470
H	-6.5717795	-2.4177731	-0.6516316
C	-5.4870905	-1.3193286	0.8309066
C	-4.6853666	-2.4190547	1.1943534
H	-4.7028584	-3.3063087	0.5613411
C	-3.8331494	-2.3904758	2.2917561
C	-3.8202008	-1.2374752	3.1096277
C	-4.6641690	-0.1819718	2.7929642
H	-4.6667434	0.7010898	3.4327405
C	-5.4860823	-0.1841899	1.6497267
C	-2.8094186	-4.4512354	1.7466116
H	-2.0252631	-5.0912425	2.1703353
H	-2.4911923	-4.1032255	0.7469605
H	-3.7331347	-5.0444838	1.6360119
C	-2.5494648	-0.0017275	4.6723948
H	-3.3904447	0.5255503	5.1584480
H	-2.1813403	0.6370290	3.8490338
C	-1.4984233	-0.2263250	5.7375785
H	-1.2415431	0.7461223	6.1936586
H	-1.9314085	-0.8591676	6.5256285
C	0.6341533	-0.2693152	4.6275934
C	0.5507806	1.0441674	4.1523103
H	-0.3316352	1.6580515	4.3315624
C	1.5952955	1.5738477	3.3985594
H	1.4878876	2.5829610	2.9904209
C	2.7454300	0.8362720	3.1173845
C	2.8367108	-0.4597802	3.6321344
H	3.7249772	-1.0629227	3.4254867
C	1.7979421	-1.0094833	4.3770625
H	1.8573080	-2.0304188	4.7609835
C	3.8109858	1.4639662	2.2505126
H	3.3149222	1.9911493	1.4244263
H	4.3385837	2.2455372	2.8239626
C	6.0717025	0.5526546	2.5047630
H	5.8902706	0.1843333	3.5269605
H	6.4747922	1.5767138	2.6012358

C	7.0516806	-0.3588636	1.7977128
H	8.0990400	-0.1070760	2.0156458
H	6.8849908	-1.4077530	2.0744513
C	7.2127971	1.0729890	-0.1970453
H	8.2880487	1.0503935	-0.4234279
H	7.0368542	1.8491588	0.5588512
C	6.3588912	1.3066236	-1.4246942
H	6.2637635	2.3878621	-1.6137784
H	6.8173294	0.8621274	-2.3259505
C	4.1006607	0.9040215	-2.2670360
H	3.4759722	0.0053232	-2.3762899
H	4.6905470	0.9634455	-3.1977015
C	3.2014219	2.1142549	-2.1410517
C	3.2985061	3.0172475	-1.0800507
H	4.1058671	2.9074089	-0.3519867
C	2.3674710	4.0348275	-0.9129975
H	2.4271236	4.7196202	-0.0640154
C	1.3048023	4.1789277	-1.8125806
C	1.2400195	3.3289738	-2.9224147
H	0.4326287	3.4118471	-3.6502002
C	2.1792761	2.3114022	-3.0708870
H	2.0843239	1.6291384	-3.9214454
C	-0.7048065	5.3773258	-2.3519517
H	-0.4307311	5.3544559	-3.4209390
H	-1.0432941	6.3985003	-2.1218008
C	-1.8482243	4.4183651	-2.1130107
H	-2.6009683	4.6080961	-2.9006090
H	-1.5132221	3.3679375	-2.1611095
C	-3.3671915	3.8457726	-0.3915838
C	-4.2235812	3.1280044	-1.2163125
H	-4.1100760	3.2246223	-2.2962231
C	-5.2093442	2.2538350	-0.7189896
C	-5.3498122	2.1231917	0.6668076
C	-4.5317039	2.9065864	1.5047235
H	-4.6649443	2.8241956	2.5839658
C	-3.5392071	3.7447287	1.0097845
C	-2.7146900	4.2857444	3.1577577
H	-2.5122938	3.2316608	3.4227574
H	-3.6775237	4.5818911	3.6095087
H	-1.9192197	4.9166684	3.5742653
C	-5.9932249	1.4294709	-1.7251373
H	-6.9527763	1.1102275	-1.3009150
H	-6.2386031	2.0711254	-2.5855721
C	-6.2444431	1.0911444	1.3255338
H	-7.1192526	0.8731737	0.7010341
H	-6.6371007	1.5110330	2.2645062
P	-0.9588588	0.4389615	-0.4371578
O	-0.5439042	1.7616816	-0.9699127
O	-0.7989164	-0.7840257	-1.4928504
O	-0.0657789	0.0078631	0.8415860
C	-0.2061935	-1.2703995	1.4346493
H	-0.3708936	-2.0474926	0.6715036
H	0.7320975	-1.4858148	1.9603855
H	-1.0307211	-1.2923928	2.1661637
C	0.1415622	-0.6818089	-2.5528156
H	1.1479975	-0.9446252	-2.1975533
H	-0.1786182	-1.3862307	-3.3287477
H	0.1575251	0.3421082	-2.9516496
C	-2.6336216	0.2548431	0.0912843
H	-2.8226428	0.9544206	0.9140623
H	-3.2968628	0.4988210	-0.7429286
H	-2.8349027	-0.7711309	0.4189207

1-H$^+$@DMMP

```
151
Energy = -4127.9227850159
P     4.3976785   -0.1568781    0.2370971
O     0.2200331   -4.1071232   -3.8033617
O    -2.3709945   -2.8389280   -3.7700755
O    -2.2749242   -0.5041349   -4.9314695
O     0.6522054    5.6122734   -1.8843674
O    -1.8821114    4.7786562   -0.9440033
O    -1.9694734    4.4415010    1.6470178
O    -2.7893054   -1.4784551    4.3748205
O    -0.2815257   -1.1251917    5.6740215
O    -2.9122449   -3.6033635    2.8569197
N     6.4254215   -0.0931201    0.4255950
N     4.3912623    0.7659960    1.6526602
N     4.6040345   -1.8314179    0.3489586
N     4.6720497    0.6138847   -1.2422899
C     6.8973835   -1.3800199   -0.1078893
H     7.9257286   -1.5848973    0.2241760
H     6.8994719   -1.3080447   -1.2039266
C     5.9108654   -2.4409796    0.3474891
H     5.9486707   -3.2967851   -0.3462161
H     6.1700984   -2.8296019    1.3507439
C     3.4558651   -2.6720510    0.6198785
H     2.7226654   -2.1041326    1.2173333
H     3.7806559   -3.5072195    1.2632322
C     2.7128431   -3.1910348   -0.5895872
C     1.6467353   -4.0743609   -0.4061645
H     1.4438169   -4.4748223    0.5913795
C     0.8037937   -4.4320661   -1.4546532
H    -0.0307592   -5.1060793   -1.2576677
C     1.0193988   -3.8955245   -2.7301083
C     2.1340178   -3.0774232   -2.9440114
H     2.3131801   -2.6927045   -3.9505477
C     2.9643109   -2.7292316   -1.8837502
H     3.8168891   -2.0691357   -2.0606586
C    -1.0108588   -4.7798757   -3.6538525
H    -0.8860487   -5.7374768   -3.1199737
H    -1.3407927   -5.0117466   -4.6763934
C    -2.0811935   -3.9610310   -2.9673129
H    -2.9709010   -4.6045842   -2.8412449
H    -1.7535437   -3.6226427   -1.9678254
C    -3.1730335   -1.8780882   -3.2557383
C    -3.1133186   -0.6112278   -3.8799944
C    -3.8856645    0.4233317   -3.3674433
H    -3.8399230    1.4058010   -3.8386571
C    -4.7303185    0.2517328   -2.2551154
C    -4.8210877   -1.0134776   -1.6641526
C    -4.0295668   -2.0566518   -2.1783469
H    -4.1016290   -3.0425201   -1.7173064
C    -2.0763834    0.7637454   -5.5039821
H    -1.3340900    0.6365044   -6.3018383
H    -1.6868755    1.4868241   -4.7641421
H    -3.0051057    1.1697483   -5.9402120
C    -5.7249048   -1.3231668   -0.4874542
H    -6.5830252   -0.6397635   -0.4778330
H    -6.1457917   -2.3295542   -0.6354740
C    -5.0169063   -1.2978637    0.8494644
C    -4.3397695   -2.4637094    1.2548401
H    -4.3968889   -3.3460582    0.6166348
C    -3.6044117   -2.5261900    2.4320539
C    -3.5487670   -1.3806945    3.2596274
C    -4.2509427   -0.2440913    2.8805451
```

H	-4.2395816	0.6311336	3.5304629
C	-4.9767512	-0.1714292	1.6782368
C	-2.9338726	-4.7680944	2.0698589
H	-2.3129341	-5.5094794	2.5878116
H	-2.5126822	-4.5899971	1.0637794
H	-3.9560538	-5.1681350	1.9584804
C	-2.5170501	-0.2897064	5.0780124
H	-3.4238657	0.0996095	5.5757756
H	-2.1690941	0.4826279	4.3712441
C	-1.4838155	-0.5639745	6.1478446
H	-1.3010726	0.3701197	6.7086654
H	-1.8829921	-1.3040044	6.8555301
C	0.5636317	-0.4107316	4.8948906
C	0.4112297	0.9442381	4.5719129
H	-0.4011735	1.5382160	4.9922910
C	1.3067731	1.5489260	3.6901670
H	1.1495869	2.5962267	3.4136984
C	2.3743482	0.8432757	3.1321345
C	2.5603836	-0.4833571	3.5286954
H	3.4063599	-1.0463249	3.1274785
C	1.6702961	-1.1070404	4.3944124
H	1.7982438	-2.1536305	4.6800426
C	3.1898303	1.4802006	2.0330922
H	2.5226878	1.5544686	1.1563271
H	3.4603184	2.5162887	2.3035741
C	5.6027446	1.0303301	2.3879770
H	5.4546990	0.8639734	3.4677748
H	5.9176852	2.0844943	2.2672961
C	6.6601177	0.0710292	1.8689969
H	7.6845174	0.4242672	2.0578284
H	6.5452457	-0.9137927	2.3417278
C	6.8461268	1.0648825	-0.3775825
H	7.9216939	1.0121810	-0.6018044
H	6.6644676	1.9705717	0.2170164
C	5.9829568	1.0761557	-1.6270713
H	5.9327759	2.1017103	-2.0280970
H	6.4110611	0.4402803	-2.4250367
C	3.5911925	0.7895190	-2.1863618
H	2.7859005	0.0879859	-1.9161883
H	3.9361757	0.4947738	-3.1936165
C	2.9608136	2.1605327	-2.2138315
C	3.1290306	3.0728798	-1.1700445
H	3.8500304	2.8553045	-0.3789182
C	2.3576436	4.2247592	-1.0932688
H	2.4577277	4.9192881	-0.2563637
C	1.3938264	4.4948937	-2.0707271
C	1.2732649	3.6322468	-3.1673820
H	0.5682175	3.8402342	-3.9737424
C	2.0510238	2.4767750	-3.2235419
H	1.9169528	1.7910587	-4.0663058
C	-0.5375264	5.8100653	-2.6122224
H	-0.3528285	5.8341732	-3.7012144
H	-0.8888466	6.8082217	-2.3149413
C	-1.6234286	4.7958444	-2.3262468
H	-2.5172702	5.0963975	-2.9036508
H	-1.3328547	3.7834691	-2.6591005
C	-2.7895027	3.8924895	-0.4821859
C	-3.6584429	3.1589209	-1.2800978
H	-3.6214506	3.2849939	-2.3624463
C	-4.6023654	2.2605325	-0.7462587
C	-4.6792596	2.1130410	0.6420260
C	-3.8035349	2.8624527	1.4513654
H	-3.8713107	2.7475553	2.5338576

```
C     -2.8535816    3.7273952    0.9227088
C     -1.8885019    4.2007305    3.0274271
H     -1.6593451    3.1404264    3.2396157
H     -2.8203072    4.4725634    3.5531913
H     -1.0708156    4.8245842    3.4101973
C     -5.4511020    1.4725720   -1.7235750
H     -6.4065801    1.1902982   -1.2646613
H     -5.7048139    2.1270016   -2.5713522
C     -5.6276471    1.1506506    1.3287021
H     -6.5217729    0.9882793    0.7144031
H     -5.9823820    1.6185464    2.2597634
P     -0.0236775   -0.2367572    0.0297695
O      0.9899498    0.8221391   -0.2267024
O     -0.2851391   -1.2002716   -1.2445548
O      0.4855609   -1.2709839    1.1510643
C     -0.3373925   -2.2411975    1.7713185
H     -0.9899264   -2.7413890    1.0395304
H      0.3254800   -2.9894077    2.2244578
H     -0.9459068   -1.7964048    2.5709295
C     -0.1543610   -0.6527129   -2.5495353
H      0.8898582   -0.3822034   -2.7582572
H     -0.4803001   -1.4203681   -3.2588701
H     -0.7853752    0.2415581   -2.6737286
C     -1.6423629    0.3320302    0.5234269
H     -1.5651667    0.8766739    1.4735955
H     -2.0269574    1.0213889   -0.2424176
H     -2.3476322   -0.5035757    0.6286495
H      2.9941834   -0.2003417    0.1034204
```

Calculations were performed with the TURBOMOLE program package.[9] Geometry optimizations were performed at the PBE0-D3/def2-SVP level of theory,[10] by using the COSMO implicit solvent model parametrized for CH_2Cl_2.[11] In the following we report the optimized structure coordinates.

```
151
Energy = -4587.2407644051
Cl     2.7056962   -0.1585242    0.0919457
P      4.7886683   -0.1911799    0.2213433
O      0.1074028   -4.0584530   -3.4814907
O     -2.4881331   -2.6930376   -3.4651924
O     -2.3465916   -0.2982647   -4.5339805
O      0.4217405    5.1629158   -1.5317603
O     -2.3855786    4.6617408   -0.8270819
O     -2.6992591    4.4773740    1.7681612
O     -2.9508004   -1.2453301    4.1479805
O     -0.3428782   -0.9099761    5.3097717
O     -2.9749461   -3.3737320    2.6327967
N      6.7749954   -0.2152358    0.3602482
N      4.8402244    0.5708612    1.7345833
N      4.9290511   -1.8780300    0.1612199
N      5.0553549    0.7119066   -1.1844990
C      7.1972910   -1.3753693   -0.4385658
```

[9] TURBOMOLE V7.5 2020, a development of University of Karlsruhe and Forschungszentrum Karlsruhe GmbH, 1989-2007, TURBOMOLE GmbH, since 2007; available from http://www.turbomole.com.

[10] a) P. A. M. Dirac, *Proc. Royal Soc. Lond. A* **1929**, *123*, 714-733; b) J. C. Slater, *Phys. Rev*. **1951**, *81*, 385-390; c) J. P. Perdew and Y. Wang, *Phys. Rev. B* **1992**, *45*, 13244-13249; d) J. P. Perdew, K. Burke and M. Ernzerhof, *Phys. Rev. Lett.* **1996**, 77, 3865-3868; e) S. Grimme, J. Antony, S. Ehrlich, H. Krieg, J. *Chem. Phys*. **2010**, *132*, 154104. f) F. Weigend, and R. Ahlrichs, *Phys. Chem. Chem. Phys*. **2005**, *7*, 3297-3305.

[11] A. Klamt and G. Schuurmann, *J. Chem. Soc.-Perkin Trans.* **1993**, 2, 799-805.

H	8.2378763	-1.6440787	-0.2088489
H	7.1389352	-1.0983557	-1.4989049
C	6.2204801	-2.4848412	-0.1109697
H	6.1556683	-3.1872089	-0.9572684
H	6.5566095	-3.0687601	0.7642745
C	3.8856675	-2.8098459	0.5634612
H	3.3051359	-2.3899954	1.3986745
H	4.3995462	-3.6925746	0.9804566
C	2.9320826	-3.2408964	-0.5272886
C	1.7370347	-3.8707054	-0.1804427
H	1.5250354	-4.0860645	0.8712299
C	0.7756676	-4.1977996	-1.1368919
H	-0.1541578	-4.6652699	-0.8118070
C	1.0010502	-3.8832779	-2.4815092
C	2.2286018	-3.3154243	-2.8487666
H	2.4017602	-3.0890018	-3.9031579
C	3.1726862	-2.9919297	-1.8821438
H	4.1011510	-2.5008726	-2.1851196
C	-1.1466575	-4.6549100	-3.2325472
H	-1.0361330	-5.5647963	-2.6175664
H	-1.5201948	-4.9656414	-4.2188543
C	-2.1781628	-3.7535145	-2.5908191
H	-3.0622679	-4.3861226	-2.3930345
H	-1.8297211	-3.3335666	-1.6305040
C	-3.3978857	-1.7814283	-3.0458149
C	-3.3245373	-0.4952518	-3.6270066
C	-4.2356454	0.4722982	-3.2230695
H	-4.1835846	1.4646948	-3.6708895
C	-5.1968758	0.2383506	-2.2204510
C	-5.2768649	-1.0381733	-1.6524012
C	-4.3799008	-2.0282196	-2.0963933
H	-4.4457879	-3.0225522	-1.6544365
C	-2.0405517	1.0235709	-4.9030473
H	-1.1455310	0.9745004	-5.5363627
H	-1.8223194	1.6423028	-4.0143668
H	-2.8562477	1.4937417	-5.4787209
C	-6.2039430	-1.3907886	-0.5032098
H	-7.0912895	-0.7463182	-0.5074470
H	-6.5717795	-2.4177731	-0.6516316
C	-5.4870905	-1.3193286	0.8309066
C	-4.6853666	-2.4190547	1.1943534
H	-4.7028584	-3.3063087	0.5613411
C	-3.8331494	-2.3904758	2.2917561
C	-3.8202008	-1.2374752	3.1096277
C	-4.6641690	-0.1819718	2.7929642
H	-4.6667434	0.7010898	3.4327405
C	-5.4860823	-0.1841899	1.6497267
C	-2.8094186	-4.4512354	1.7466116
H	-2.0252631	-5.0912425	2.1703353
H	-2.4911923	-4.1032255	0.7469605
H	-3.7331347	-5.0444838	1.6360119
C	-2.5494648	-0.0017275	4.6723948
H	-3.3904447	0.5255503	5.1584480
H	-2.1813403	0.6370290	3.8490338
C	-1.4984233	-0.2263250	5.7375785
H	-1.2415431	0.7461223	6.1936586
H	-1.9314085	-0.8591676	6.5256285
C	0.6341533	-0.2693152	4.6275934
C	0.5507806	1.0441674	4.1523103
H	-0.3316352	1.6580515	4.3315624
C	1.5952955	1.5738477	3.3985594
H	1.4878876	2.5829610	2.9904209
C	2.7454300	0.8362720	3.1173845

C	2.8367108	-0.4597802	3.6321344
H	3.7249772	-1.0629227	3.4254867
C	1.7979421	-1.0094833	4.3770625
H	1.8573080	-2.0304188	4.7609835
C	3.8109858	1.4639662	2.2505126
H	3.3149222	1.9911493	1.4244263
H	4.3385837	2.2455372	2.8239626
C	6.0717025	0.5526546	2.5047630
H	5.8902706	0.1843333	3.5269605
H	6.4747922	1.5767138	2.6012358
C	7.0516806	-0.3588636	1.7977128
H	8.0990400	-0.1070760	2.0156458
H	6.8849908	-1.4077530	2.0744513
C	7.2127971	1.0729890	-0.1970453
H	8.2880487	1.0503935	-0.4234279
H	7.0368542	1.8491588	0.5588512
C	6.3588912	1.3066236	-1.4246942
H	6.2637635	2.3878621	-1.6137784
H	6.8173294	0.8621274	-2.3259505
C	4.1006607	0.9040215	-2.2670360
H	3.4759722	0.0053232	-2.3762899
H	4.6905470	0.9634455	-3.1977015
C	3.2014219	2.1142549	-2.1410517
C	3.2985061	3.0172475	-1.0800507
H	4.1058671	2.9074089	-0.3519867
C	2.3674710	4.0348275	-0.9129975
H	2.4271236	4.7196202	-0.0640154
C	1.3048023	4.1789277	-1.8125806
C	1.2400195	3.3289738	-2.9224147
H	0.4326287	3.4118471	-3.6502002
C	2.1792761	2.3114022	-3.0708870
H	2.0843239	1.6291384	-3.9214454
C	-0.7048065	5.3773258	-2.3519517
H	-0.4307311	5.3544559	-3.4209390
H	-1.0432941	6.3985003	-2.1218008
C	-1.8482243	4.4183651	-2.1130107
H	-2.6009683	4.6080961	-2.9006090
H	-1.5132221	3.3679375	-2.1611095
C	-3.3671915	3.8457726	-0.3915838
C	-4.2235812	3.1280044	-1.2163125
H	-4.1100760	3.2246223	-2.2962231
C	-5.2093442	2.2538350	-0.7189896
C	-5.3498122	2.1231917	0.6668076
C	-4.5317039	2.9065864	1.5047235
H	-4.6649443	2.8241956	2.5839658
C	-3.5392071	3.7447287	1.0097845
C	-2.7146900	4.2857444	3.1577577
H	-2.5122938	3.2316608	3.4227574
H	-3.6775237	4.5818911	3.6095087
H	-1.9192197	4.9166684	3.5742653
C	-5.9932249	1.4294709	-1.7251373
H	-6.9527763	1.1102275	-1.3009150
H	-6.2386031	2.0711254	-2.5855721
C	-6.2444431	1.0911444	1.3255338
H	-7.1192526	0.8731737	0.7010341
H	-6.6371007	1.5110330	2.2645062
P	-0.9588588	0.4389615	-0.4371578
O	-0.5439042	1.7616816	-0.9699127
O	-0.7989164	-0.7840257	-1.4928504
O	-0.0657789	0.0078631	0.8415860
C	-0.2061935	-1.2703995	1.4346493
H	-0.3708936	-2.0474926	0.6715036
H	0.7320975	-1.4858148	1.9603855

H	-1.0307211	-1.2923928	2.1661637
C	0.1415622	-0.6818089	-2.5528156
H	1.1479975	-0.9446252	-2.1975533
H	-0.1786182	-1.3862307	-3.3287477
H	0.1575251	0.3421082	-2.9516496
C	-2.6336216	0.2548431	0.0912843
H	-2.8226428	0.9544206	0.9140623
H	-3.2968628	0.4988210	-0.7429286
H	-2.8349027	-0.7711309	0.4189207
